# HiFIseek: Gene-specific enrichment of high-impact mutations in associated genomic regions

**DOI:** 10.1371/journal.pcbi.1014842

**Published:** 2026-10-06

**Authors:** Rafael Riudavets Puig, Denise G. O’Mahony, Ina Skaara Brorson, Ksenia Sokolova, Vessela N. Kristensen, Anthony Mathelier

**Affiliations:** 1 Norwegian Center for Molecular Biosciences and Medicine (NCMBM), Nordic EMBL Partnership, University of Oslo, Oslo, Norway; 2 Department of Medical Genetics, Oslo University Hospital, Oslo, Norway; 3 Institute for Clinical Medicine, Faculty of Medicine, University of Oslo, Oslo, Norway; 4 Biostatistics Unit, The Cyprus Institute of Neurology and Genetics, Nicosia, Cyprus; 5 Princeton Precision Health, Princeton University, Princeton, New Jersey, United States of America; 6 Flatiron Institute, Simons Foundation, New York, New York, United States of America; 7 Bioinformatics in Life Science (BiLS) Initiative, Department of Informatics, University of Oslo, Oslo, Norway; CANADA

## Abstract

Transcription is regulated through the sequence-specific binding of transcription factors (TFs) to cis-regulatory regions (CRRs); DNA variants in CRRs can disrupt TF-DNA interactions and gene regulatory networks through a cascading effect. Despite a few cases of reported recurrent cis-regulatory variants (CRVs), another approach for detecting non-coding functional events relies on combining gene-CRR associations capturing the regulatory elements controlling gene transcription, and functional impact (FI) scores quantifying the deleterious effect of variants. While many methods exist for each, how much they agree and how strongly the choice of CRR-FI score combination impacts the prediction of cis-regulatory altered genes has not been systematically assessed. Here, we demonstrate that gene-CRR associations and FI scoring methods exhibit little consensus, highlighting the impact of the choice of a specific CRR-FI score combination in a given analysis. These observations point to the need for a systematic approach to evaluate several gene-CRR and FI scoring methods in a unified framework. To address this gap, we developed HiFIseek, a Snakemake pipeline of two enrichment detection methods exploring the enrichment of high FI variants in CRRs associated with a gene using all region-score combinations. We applied HiFIseek to detect genes exhibiting high functional impact CRVs in eight cancer cohorts and a set of breast cancer risk-associated single-nucleotide polymorphisms (SNPs). We demonstrate that cancer driver genes exhibit a higher frequency of FI CRVs compared to non-cancer genes. In the cancer cohorts, HiFIseek detected genes pointing to cancer-related pathways with enrichment for high FI CRVs despite a small degree of agreement across CRR-FI score combinations. In the SNP cohort, HiFIseek found again small consensus across CRR-score combinations, one of which returned 18 known cancer-related genes, including *BRCA1*, showing an enrichment of high-impact CRVs.

## Author summary

While most of our genome does not code for proteins, it contains DNA segments that control the activation of genes. When mutations occur in these segments, they can disrupt how genes are regulated and may contribute to diseases such as cancer. However, identifying which mutations in these segments are important is an ongoing challenge. In this study, we show that computational methods largely disagree when assessing the functional impact of a mutation and which gene will likely be impacted. To provide a broader perspective on the prediction of genes associated with cancer through mutations in regulatory DNA segments, we developed HiFIseek, a flexible computational pipeline that systematically tests every combination of methods to find genes whose regulatory segments are unusually enriched for high-impact mutations. Applying HiFIseek to cancer datasets and breast cancer risk variants, we recovered known cancer genes and highlighted candidates worth further investigation.

## Introduction

Transcriptional regulation is a highly complex mechanism involving several biological processes. One of its most essential elements consists of the interplay between transcription factors (TFs) and cis-regulatory regions (CRRs) [1]. TFs are DNA-binding proteins with a selective affinity for short DNA sequences known as their TF binding sites (TFBSs). CRRs are genomic regions that play a crucial role in transcriptional regulation. Depending on their effect and location relative to the transcription start sites of their transcriptional targets, CRRs are classified into promoters, enhancers, and silencers. While promoters are located directly upstream of their transcriptional targets, enhancers and silencers can be found hundreds of kilobases away and in any orientation. Enhancers and silencers are involved in transcriptional activation and repression, respectively. The interplay between TFs and CRRs is achieved through the presence of TFBSs in CRRs, enabling their context-specific interaction. Because this interaction is sequence-dependent, some sequence variants may alter transcriptional regulation.

Although many somatic cancer driver mutations are mapped to protein-coding regions, the vast majority of somatic mutations falls within the non-protein-coding space of the genome [2], where most of them are thought to be passenger events. Similarly, most disease-associated single-nucleotide polymorphisms (SNPs) are found in non-protein-coding regions [3]. These observations suggest there is potential for driver events outside the protein-coding space. However, understanding which of these non-coding variants are drivers and their underlying mechanisms is an ongoing challenge, even more challenging than it is for their protein-coding counterpart. This is mainly due to the large genomic distance that non-coding variants may have to a gene, the complexity of gene regulation and the combinatorics of TF-CRR-gene interactions leading to many possible ways to disrupt a gene’s cis-regulatory space. Additionally, the impact of the same variant can be larger or lower depending on factors such as cell type and state [4].

Variant recurrence is commonly used to identify variants that show signs of positive selection in disease. Currently, only a handful of recurring events have been attributed to cis-regulatory variants (CRVs) in the context of cancer [5]. The most well-known example observed across different cancer types involves the recurring mutations of the TERT promoter, which were found to introduce new binding sites for TFs of the ETS family and lead to the reactivation of the TERT gene’s transcription [6,7]. Besides recurrence, alternative ways to detect candidate non-protein-coding driver events have been explored. The effect of CRVs on TF binding revealed cases where the detected genes showed a significant change in gene expression [8]. CRRs bound by known prostate-cancer-related TFs such as AR, FOXA1, HOXB13, and SOX9 were enriched for CRVs and these non-protein-coding mutations led to the overexpression of MYC [9]. Outside the context of cancer, in human primary hematopoietic cells, promoter capture HiC (pc-HiC) revealed genomic regions near gene promoters (pc-HiC derived CRRs) with enrichment for expression quantitative trait loci (eQTLs) [10]. The single SNP rs1421085 was shown to disrupt the binding of ARID5B, resulting in a derepression of IRX3 and IRX5 during adipocyte differentiation, impacting obesity [11].

Another strategy for identifying candidate driver events involves analysing the enrichment of functional mutations in sets of associated genomic regions. An example is OncodriveFML [12], which assesses whether a set of genomic regions exhibits a higher-than-expected average functional impact score by comparing the observed values to a permutation-based expectation. However, permutation-based methods can be computationally intensive. Regardless of the specific methodology used to assess enrichment, two key elements are the annotations used to group genomic regions and the scoring of the functional impact (FI) of mutations. For example, exploring gene CRRs for high-impact mutations requires knowing all relevant CRRs, their transcriptional target genes, and the effects of mutations within them.

Dissecting which CRRs are relevant for the transcriptional regulation of each gene remains a challenging task. Although a broad range of methods attempt to tackle this problem, gold-standard methods and resources are still lacking in the field. The Genehancer [13] resource consists of a collection of known and predicted gene-CRR associations. Other methods use specific types of data to produce gene-CRR links. For example, epiregio gene-CRR associations are inferred by STITCHIT [14], which predicts gene expression using DNase I hypersensitivity sites (DHS). Another method is the activity-by-contact (ABC) model [15,16], which uses DHS, H3K27Ac, and chromatin conformation data to determine each gene’s CRRs while accounting for the activity level of each CRR. Other experimental methods such as pc-Hi-C [17] are specifically designed to obtain the genomic regions in close spatial proximity to a selected set of gene promoters.

Similarly to the case of gene-CRR associations, a gold-standard methodology scoring the functional impact of non-protein-coding variants is still lacking, despite the development of many different approaches in recent years. Some of these utilize available annotations to predict the effect of variants in a supervised manner. For example, the CADD [18] and DANN [19] methods classify a variant as deleterious or neutral based on 60 different annotations using logistic regression and a deep learning framework, respectively. Similarly, LINSIGHT [20] combines a generalized linear model with a probabilistic graphical model to integrate signals from molecular evolution and genomic data. While such approaches leverage existing biological knowledge to infer variant impact, the FI of poorly annotated variants may be underestimated. Likewise, the FI of non-protein-coding variants may be overlooked when analysed together with protein-coding variants. Other methods, such as EIGEN and EIGEN-PC [21], employ an unsupervised approach that combines different annotations. Common to these methods is their aim to provide a single score for each possible variant in the genome, regardless of cellular context. However, the same variant may have different functional consequences depending on the cell type or condition. This limitation is addressed by the Sei framework [22], which utilizes a deep learning model to predict TF binding and histone modifications from DNA sequences across multiple cellular contexts. The predicted chromatin profiles are then classified into sequence classes, some of which have been shown to represent specific cell types. By performing *in silico* mutagenesis while focusing on selected sequence classes could be used to better estimate the impact of variants in a cell type of interest.

Studies exploring variants in the cis-regulatory space of a gene typically rely on a single combination of gene-CRR association and variant scoring approaches. However, given the complexity and context-specificity of gene regulation, it is unclear if and when to prefer one combination over another; and the extent to which results depend on this choice remains largely unexplored. This provides a methodological gap to fill. Exploring all CRR-score combinations can therefore be used to explore a larger search space and possibly identify instances where multiple combinations agree.

In this work, we present evidence that gene-CRR association and FI scoring methods show a small level of consensus. Based on this observation, we further searched for the enrichment of high FI variants in each gene’s cis-regulatory space. To address the methodological gap, we developed HiFIseek. The central contribution of HiFIseek is a deliberately agnostic design: rather than committing to any single gene-CRR association or FI scoring method, HiFIseek systematically runs all user-provided combinations and provides an overview of the identified genes. HiFIseek is implemented as a Snakemake [23] pipeline that scales to any number of cohorts, associations, and FI scoring methods. For each combination, HiFIseek computes the enrichment of high-impact variants using two different methods: (i) OncodriveFML, and (ii) a new analytical approach orthogonal to the OncodriveFML methodology. We apply this workflow to study each gene’s CRRs in seven cancer cohorts from The Cancer Genome Atlas (TCGA) [24], a breast cancer cohort from the International Cancer Genome Consortium (ICGC) [25], and a set of germline SNPs associated with increased risk of developing breast cancer from the Norwegian Breast Cancer Study (NBCS) [26]. We employed four distinct gene-CRR association methods and eight variant scoring methods. We observed that cancer-related driver genes contain higher FI variants in their CRRs compared to non-cancer-related genes. In the cancer cohorts, results varied across CRR-score combinations, between 1726 and 4979 distinct genes across all cohorts exhibited an enrichment of high-impact CRVs (mean: 2935.75, median: 2826.5). Gene ontology functional enrichment analyses across the cohorts revealed the enrichment of biological processes associated with cancer, despite a small intersection of predictions across combinations of CRR-gene associations and FI scoring methods. At the germline level, HiFIseek identified genes enriched for high-impact regulatory variation, including cancer driver genes such as *BRCA1,* demonstrating that context-specific combinations reveal distinct and biologically relevant signals.

## Results

### Gene-CRR association methods show small overlap

First, we conducted a series of exploratory analyses to gain a deeper understanding of the differences between all the gene-CRR association methods evaluated here. We started by exploring the distributions of the sizes of each gene’s associated CRRs. In most approaches, the average size of identified regions was approximately 1000 bp, except the ones found by Genehancer, which were 1–2 orders of magnitude larger (Fig A in S1 Appendix). We then explored agreement across gene-CRR association methods. For each gene, we calculated the proportion of overlap between its CRRs identified by different approaches. In many cases, we observed a small consensus across gene-CRR associations. Genehancer regions produced the largest overlap with other region sets, which can be explained by the fact that GeneHancer regions tend to be larger, increasing the likelihood of overlap with regions identified by alternative approaches. Among all other approaches, ABC-derived gene CRRs showed the strongest consensus with all other methods except epiregio, whose regions did not overlap with any of the other gene-CRR association methods (Fig 1). We observed a strong agreement between ABC regions derived from different cell types (0.11 - 0.37 median overlap proportion). ABC regions also showed some overlap with intronic and promoter regions.

**Fig 1:**
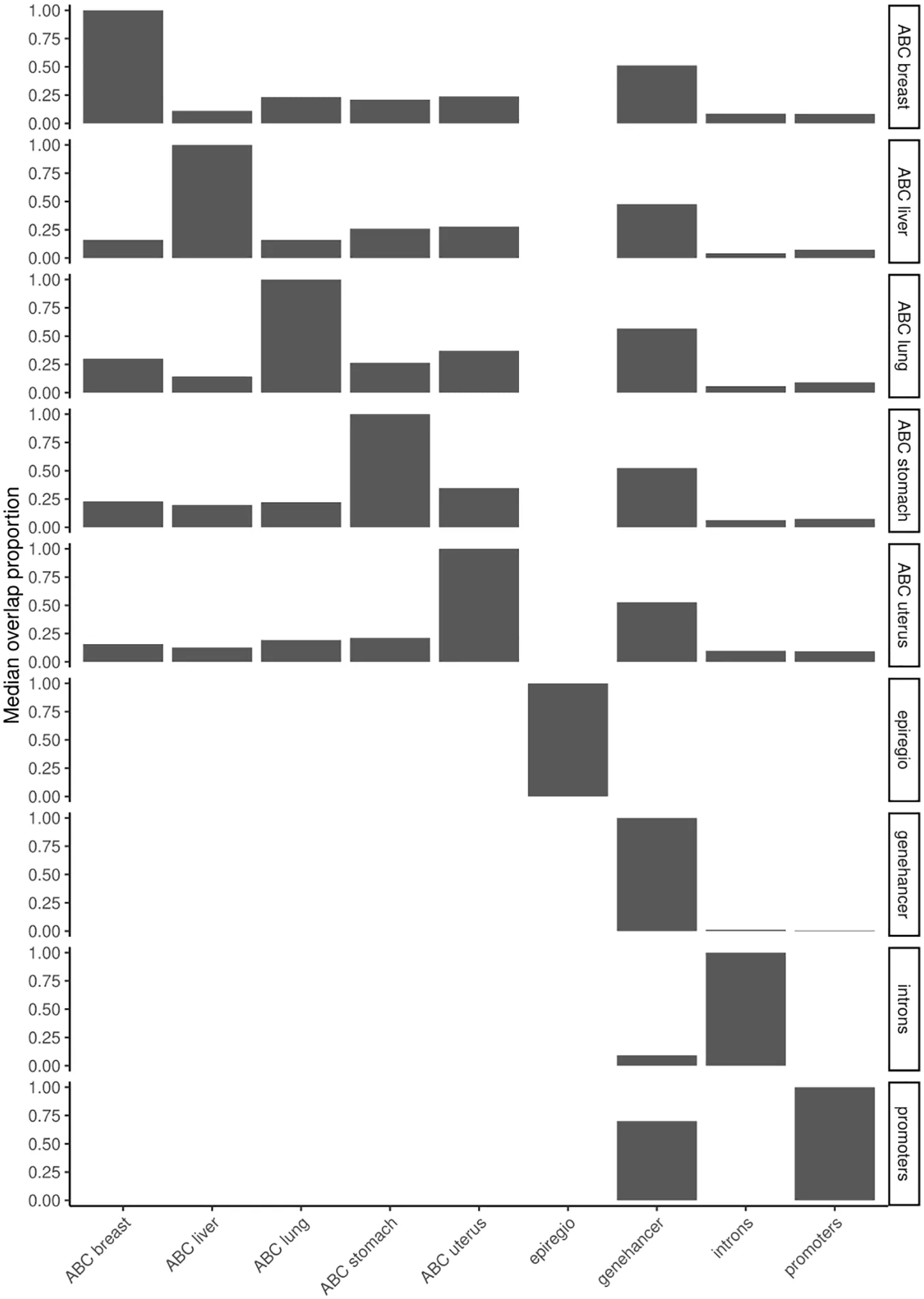
Gene-CRR association methods show small consensus. The plot provides pairwise analyses of the degree of intersections as median overlap proportion (y-axis) between CRR-gene associations using different methods (listed in rows and columns). The row facet labels indicate the reference CRR-gene association method while the columns on the x-axis indicate the CRR-gene association methods compared against. The median overlap proportion (y-axis of the plots) indicates the fraction of the reference association showing an overlap with the method being compared against.

In summary, these results point to gaps in the agreement across the existing gene-CRR association methods, underscoring the significant impact of the method of choice on the established gene-CRR associations.

### Functional impact scores show little consensus

We next compared the distributions of variant impact scores across scoring methods. We observed substantial differences in the range and distribution of score values between the different scoring approaches, as shown in Fig B in S1 Appendix. For example, CADD and DANN scores utilize different models to infer the impact of variants and their score distributions are vastly different when analyzing the same data.

We explored the correlation between methods when comparing the impact scores for the same set of variants. We randomly selected 10,000 distinct non-protein-coding mutations from all TCGA cohorts and compared their impact scores across all methods. As shown above for gene-CRR associations, the impact scoring approaches showed little consensus on the impact of the selected mutations (Fig 2).

**Fig 2:**
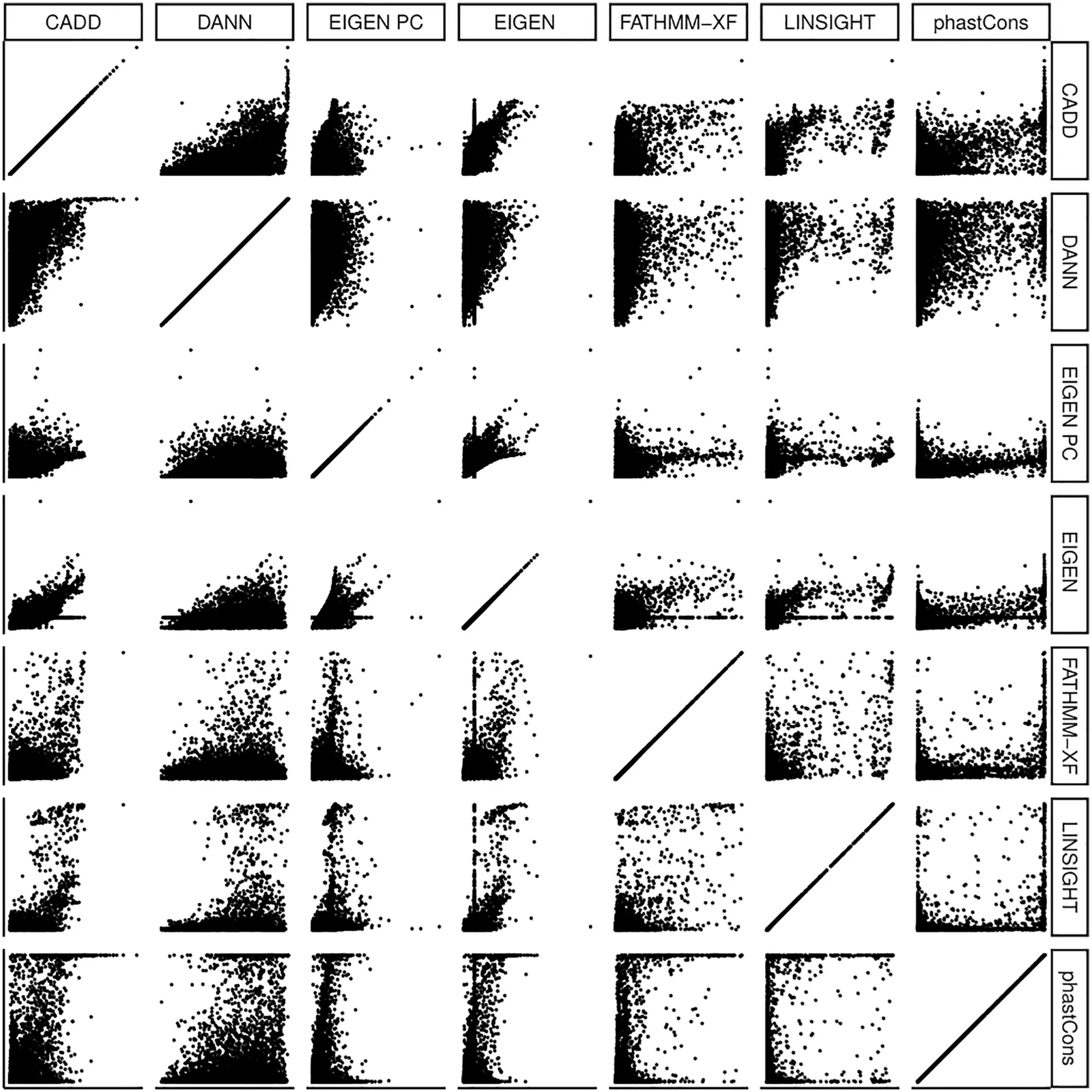
Correlation of variant impact scores between FI scoring methods shows little agreement. The plot provides pairwise comparisons of the FI scores obtained for 10,000 randomly selected variants from several FI scoring methods. Each scatter plot provides the FI score of a method listed as column against a method listed as row.

These results highlight disagreement among FI scoring approaches when examining the potential impact of variants. Such differences are likely to affect the results of methodologies using these scores to detect enrichment of FI variants in a set of associated genomic regions.

### Cancer gene CRRs show significantly higher functional impact scores

We used the different methods evaluated here to study the functional impact patterns of somatic variants from seven TCGA cancer cohorts and the ICGC breast cancer cohort (Fig C in S1 Appendix). The TCGA cancer cohorts studied include: BRCA-US (breast invasive carcinoma), HNSC-US (head and neck squamous cell carcinoma), LIHC-US (liver hepatocellular carcinoma), LUAD-US (lung adenocarcinoma), LUSC-US (lung squamous cell carcinoma), STAD-US (stomach adenocarcinoma), and UCEC-US (uterine corpus endometrial carcinoma). For each cancer type, the number of distinct mutations located in protein-coding or non-protein-coding regions were counted. Using RefSeq annotations, we investigated the number of distinct mutations located in gene promoters, 5’ UTRs, coding exons, introns, and 3’ UTRs. As expected, the majority of mutations occurred within the non-protein-coding genome, with intronic regions showing similar numbers of mutations to other non-protein-coding regions (Fig D in S1 Appendix).

We explored the following properties for cancer driver versus non-cancer genes: (i) the distribution of gene region mutation rates stratified by gene-CRR association method; and (ii) the distribution of FI scores for the observed mutations stratified by association and FI scoring methods.

*Distribution of gene CRR mutation rates stratified by association method.* We studied the distributions of mutation rates in the CRRs, promoters, 5’ UTRs, coding exons, introns, and 3’ UTRs of cancer driver and non-cancer genes. We used the following annotation methods to define gene-CRR associations: epiregio, Genehancer, and ABC when data for a relevant cell type were available. We observed significantly higher mutation rates in 5’ UTRs in all cancer cohorts, epiregio CRRs in the ICGC cohort, and ABC-derived CRRs in the TCGA LIHC cohort (Figs E-L in S1 Appendix). This contrasted with the remaining of the studied regions, where cancer driver genes did not show significantly higher mutation rates in many of the studied regions.

*Distribution of FI scores for the observed mutations stratified by association and FI scoring methods.* We compared the FI scores of mutations in cancer driver versus non-cancer genes for each cohort across various CRR-gene association and scoring methods. We used the following FI scoring methods: CADD, DANN, EIGEN, EIGEN PC, FATHMM-XF, LINSIGHT, and phastCons across 100 vertebrate species. This analysis resulted in many region-score combinations where cancer driver genes showed significantly higher impact CRVs than non-cancer genes (Fig 3 and Figs M-T in S1 Appendix). The strongest agreement across all methods’ scores was seen in promoter regions. Cancer driver genes showed significantly higher FI scores than non-cancer genes in virtually all scores and cohorts. ABC and Genehancer regions showed more variation across scores and cohorts and epiregio-derived CRRs regions did not show higher FI CRVs in cancer genes except in the HNSC cohort. Based on these results, we decided to search for gene CRRs with enrichment for high FI CRVs, using all combinations of CRR-scores.

**Fig 3:**
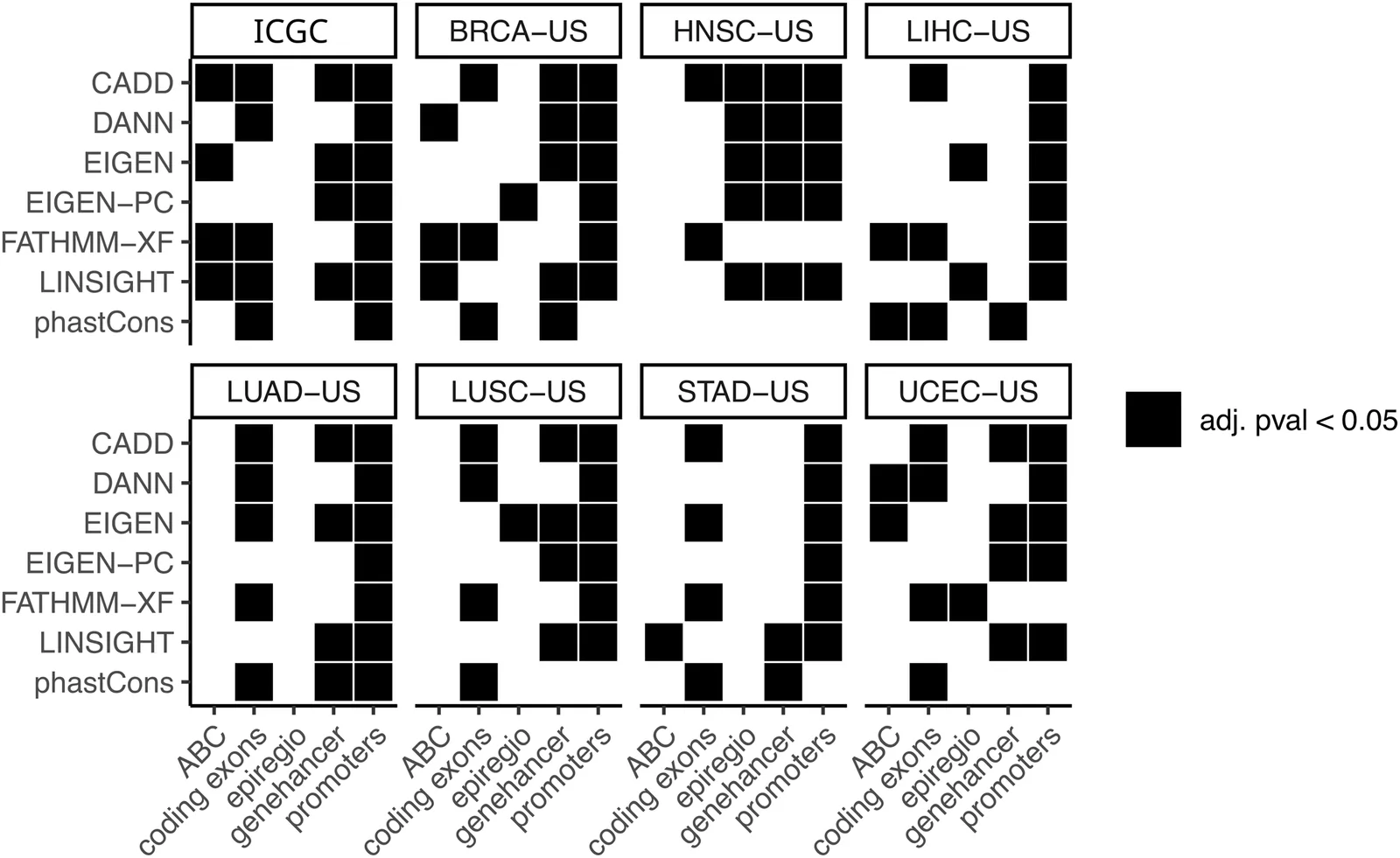
Mutations in cancer driver genes show significantly higher FI scores compared to non-cancer genes. Comparison of cancer driver versus non-cancer FI scores using different CRR-gene associations (x-axis) and FI scoring (y-axis) methods. Black squares represent those combinations for which cancer genes show significantly higher (adjusted p-value ≤ 0.05) FI scores than non-cancer genes. Each panel provides the results for a given cancer type. Note that ABC associations were not available for the HNSC-US cohort.

### HiFIseek: A high-performance method to detect the enrichment of high-impact variants in a set of associated genomic regions

The lack of consensus across gene-CRR association and FI scoring methods prompted us to develop a tool to systematically explore all possible CRR-score combinations in an automated way compared to previous methods (Table A in S1 Appendix). To this end, we developed HiFIseek to study the enrichment of high impact FI variants in sets of associated genomic regions. HiFIseek is a Snakemake pipeline that explores all combinations of user-provided association and scoring methods across any number of cohorts. HiFIseek employs two distinct enrichment detection methods to identify genes exhibiting an enrichment of high FI variants. The first one is OncodriveFML, which compares the average impact score of a set of observed variants to a permutation-based expected average. The second one, which we refer to as OncodriveFML-analytic, is a new analytical approach to the OncodriveFML framework that utilizes the sum of observed FI scores rather than their mean, and computes significance analytically rather than by permutation. First, the sum can reflect the accumulation of high-impact variants in CRRs. Second, the distribution of a sum of independent scores is well approximated by a normal distribution, which allows p-values to be computed in closed form from the variant scores distribution with the cohort’s mutational signature, removing the need for permutations (see Methods). HiFIseek used the following modules for further exploration of genes passing a determined enrichment significance threshold: (i) differential expression analysis when expression data is available, and (ii) ontology enrichment analyses using gene ontology (GO) [27,28].

We first assessed our new analytical framework on protein-coding somatic mutations to determine whether it could recover well-known cancer-related genes. In all cohorts, OncodriveFML-analytic successfully detected established cancer genes (Fig 4 and Figs U-AB in S1 Appendix). For example, the top 10 genes when studying the ICGC breast cancer cohort with CADD scores were: *TP53*, *TMC6*, *PIK3CA*, *RB1*, *MAP3K1*, *NF1*, *ARID1A*, *APBA2*, *PTEN*, and *CDKN2A*, eight of which are well-known cancer-related genes. In general, HiFIseek tended to detect more genes than OncodriveFML. Overall, we observed a small agreement across scoring approaches, except in well-studied cancer-related genes such as *TP53* or *PIK3CA*. These results highlight not only the differences across FI scoring approaches but also their convergence in only the most well-known cancer-related genes.

**Fig 4:**
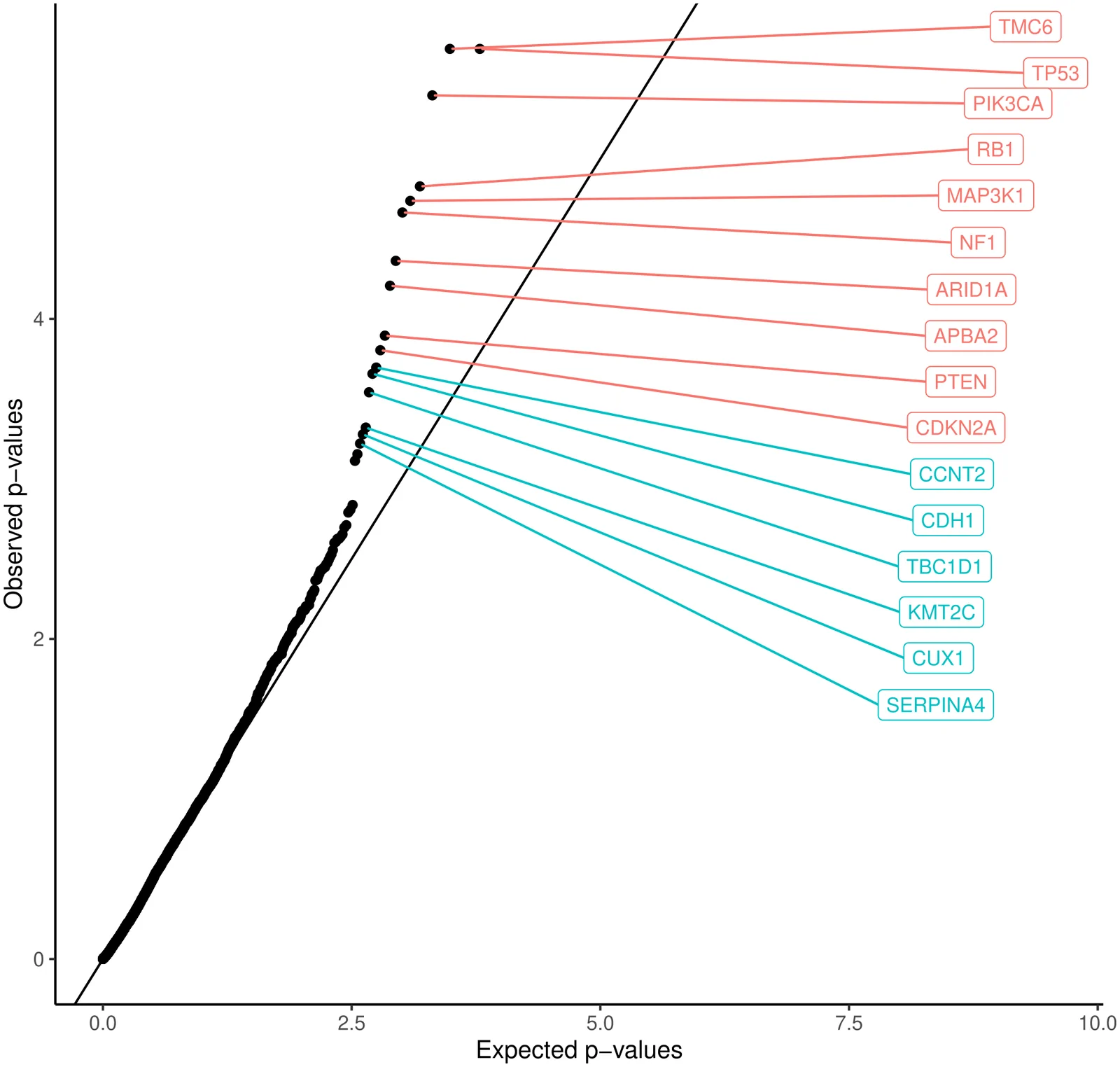
Enrichment of high functional impact mutations in the ICGC breast cancer cohort detected by OncodriveFML-analytic using CADD scores. Quantile-quantile plot showing the expected (x-axis) versus observed (y-axis) distribution of the log10(p-values) obtained by OncodriveFML-analytic. The diagonal line represents the expected distribution of log10 (p-values) if observed p-values follow a uniform distribution. Points deviating from the diagonal towards the upper-left corner indicate significant hits. Genes showing an adjusted p-value < 0.1 are labelled in red, while genes with an adjusted p-value between 0.1 and 0.25 are labelled in green.

We next benchmarked OncodriveFML-analytic against OncodriveFML to evaluate their performance in terms of computational requirements. Specifically, we aimed to investigate the required time and memory usage when running OncodriveFML-analytic or OncodriveFML with different numbers of cores, along with gene-CRR associations and cohorts of increasing size. The dataset used in the benchmarking consisted of: (i) mutations from the ICGC breast cancer (2,277,413 mutations), BRCA-US (1,062,470 mutations), and LIHC-US (717,885 mutations) cohorts; (ii) CADD scores; and (iii) promoter (1000 bp median size), coding exons (1341 bp median size) and Genehancer (17,890 bp median size) as gene-region associations, which were chosen based on the distribution of region sizes (Fig A in S1 Appendix). We ran the analyses using 5, 10, 20, 50, or 100 available cores for parallelization and recorded the elapsed time and maximal RAM usage for each analysis. In all analyses, OncodriveFML was run using 1,000,000 permutations. In all cases, OncodriveFML-analytic showed better performance than OncodriveFML both in terms of computation time and required resources (Fig AC panels A-B in S1 Appendix). Comparison of the results showed a general agreement between the enrichment results of OncodriveFML-analytic and OncodriveFML (Fig AC panel C in S1 Appendix), with OncodriveFML-analytic tending to yield a more conservative significance estimation than OncodriveFML, particularly for increasing gene-region sizes.

### Exploring the enrichment for high functional impact cis-regulatory variants in eight cancer cohorts with HiFIseek

We explored the enrichment of high FI somatic mutations in gene CRRs using HiFIseek on all combinations of 8 FI scores (CADD, DANN, EIGEN, EIGEN-PC, FATHMM-XF, LINSIGHT, Sei, and phastCons) and 4 gene-CRR associations (promoter regions, ABC, epiregio, and Genehancer). In general, we observed little consensus when comparing all gene-CRR-score combinations within a cohort, showing again the influence of a given association and scoring combination in the analysis (Fig 5 and Figs AD-AJ in S1 Appendix). Genehancer regions showed the highest number of genes with a significant enrichment of FI CRVs across all scores and most cohorts. With some exceptions, the majority of genes showing an enrichment of FI CRVs have not been reported to be related to cancer drivers. Recognizing the disparity in results, we performed, for each cohort, a gene set enrichment analysis using gene ontology biological process (GOBP) terms considering all genes predicted. We observed recurring enrichment of several GOBP terms across cohorts (Fig 6). For instance, from the most common enriched terms, several can be linked to cancer, such as signaling, apoptotic process, cell cycle and cell proliferation-related terms.

**Fig 5:**
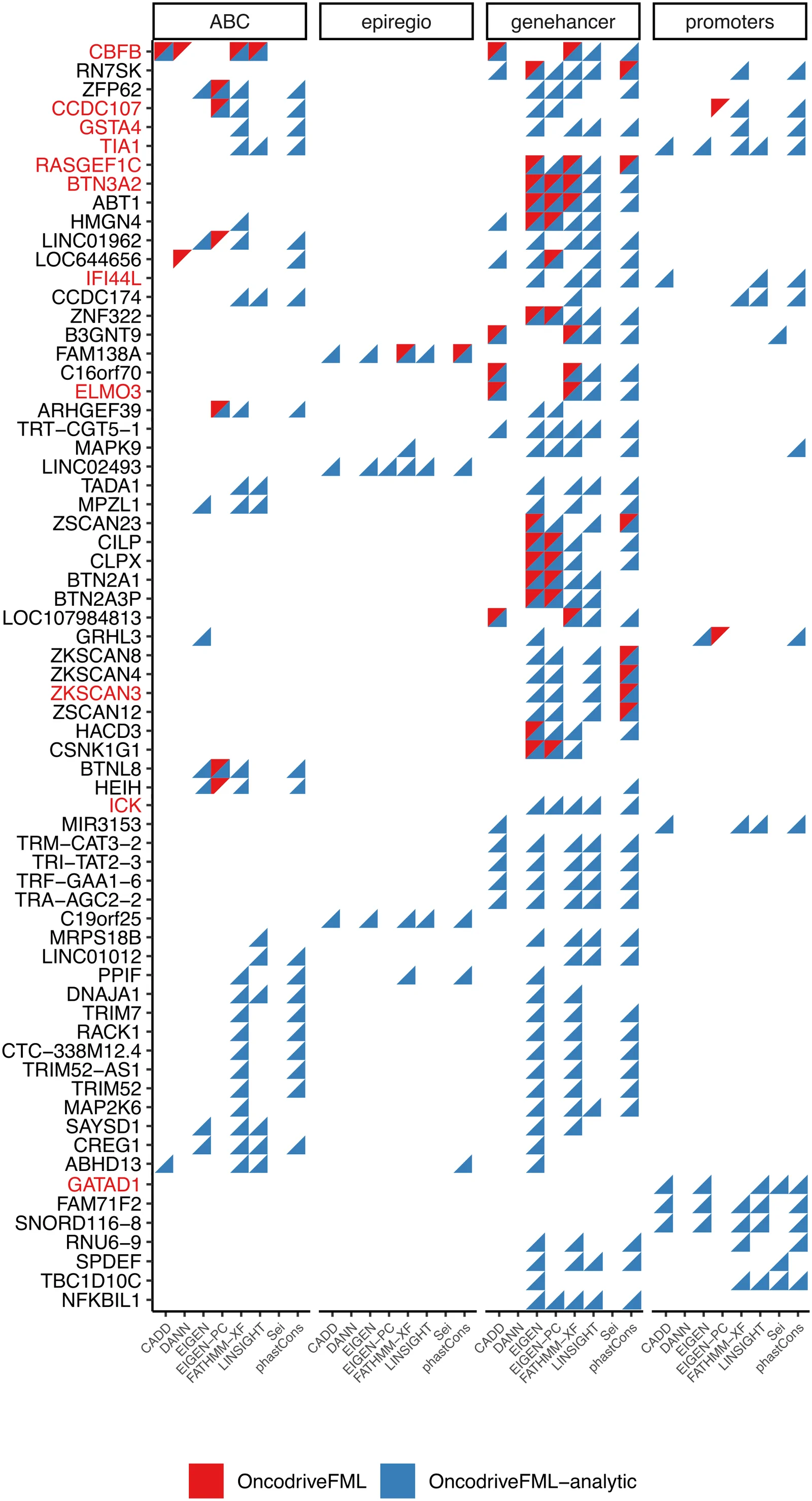
Detected genes across CRR-score combinations in the ICGC breast cancer cohort. Genes (y-axis) showing an enrichment (adjusted-pval ≤ 0.05) of FI CRVs in different score (x-axis) and association (facet) combinations detected by either OncodriveFML (red triangles) or OncodriveFML-analytic (blue triangles). Only genes detected in at least five or more CRR-score combinations and/or enrichment methods are shown. Gene names in red are listed as cancer driver genes.

**Fig 6:**
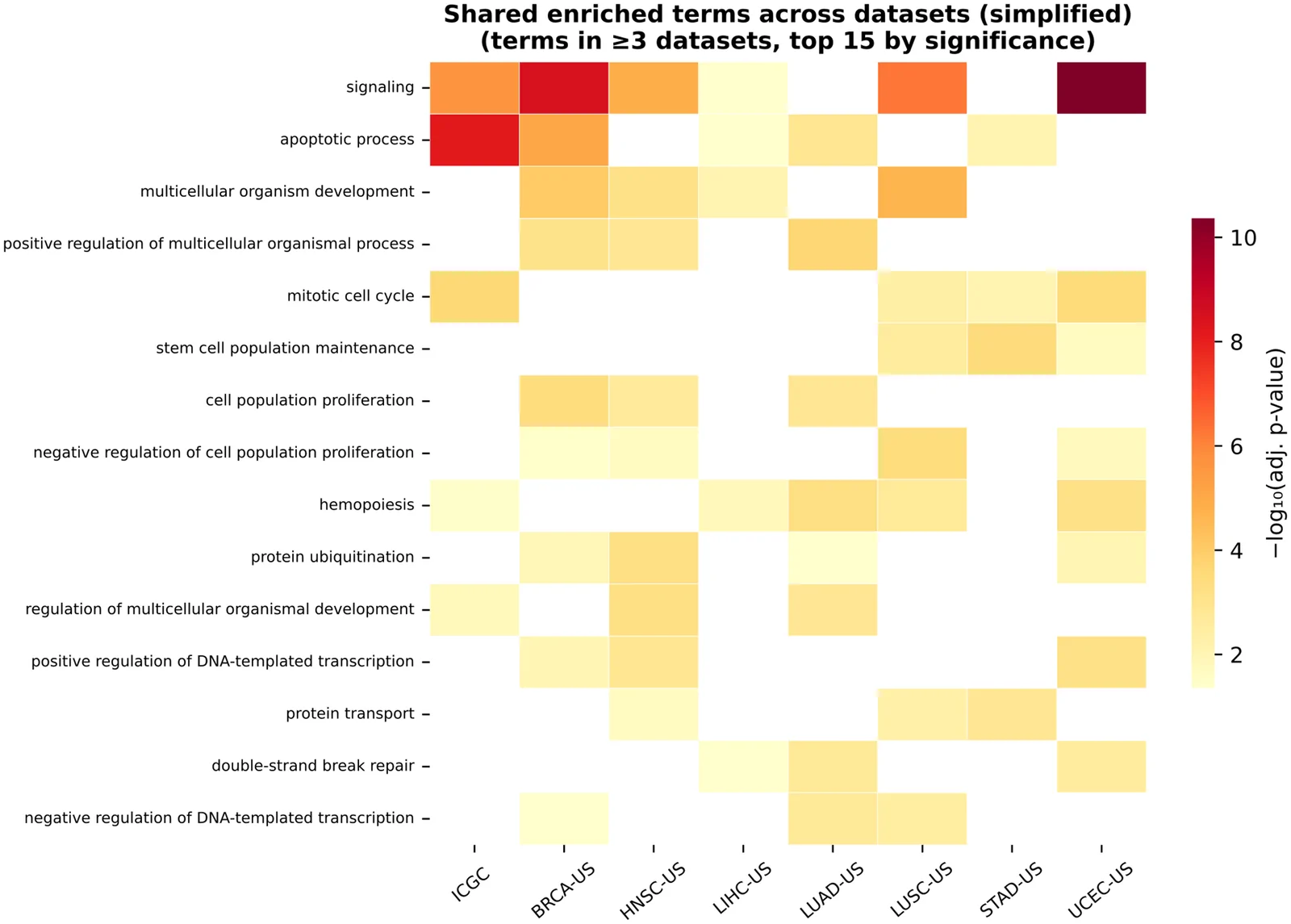
Functional Gene Ontology enrichment. The heatmap provides the list of most recurrent Gene Ontology biological processes (GOBP) that were identified in at least three cohorts. The cohorts are provided as columns and the GOBP terms as rows. The color intensity reflects the -log10 adjusted p-value of the term in the cohort (white indicates it was not found significant in the corresponding cohort).

Studying the ICGC breast cancer cohort with HiFIseek resulted in 67 genes showing an enrichment of FI CRVs in at least five combinations, 11 of which have been previously reported to be related to cancer (Fig 5). Among the detected genes, CBFB showed the strongest agreement across FI scores, associations, and enrichment detection methods. This gene has been previously reported to have a tumor suppressor role in breast cancer [29]. Another example emerged when comparing results across the ICGC and TCGA breast cancer cohorts; we observed that RASGEF1C showed enrichment of FI CRVs in both. This protein is a guanine nucleotide exchange factor, an important molecular function in the activation of Ras signaling, previously associated with lung cancer [30,31].

Given the role of CRRs in transcriptional regulation, we hypothesized that genes with an enrichment of FI CRVs may show changes in their transcriptional levels. To test this, we performed a differential expression analysis for all genes detected by HiFIseek, grouping patients according to the mutation status of each gene’s CRRs. Overall, many genes with an enrichment of FI CRVs did not show changes in their expression. For example, we did not observe significant changes in the expression levels of *CBFB* or *RASGEF1C*, although the latter showed a higher, but not significant level of expression (Fig AK in S1 Appendix).

### Breast cancer risk SNPs analysed by HiFIseek show an enrichment for high impact non-protein-coding variants in known breast cancer-related genes

We next applied HiFIseek on a set of germline SNPs from the Norwegian Breast Cancer Study from the Breast Cancer Association Consortium associated with an increased risk of developing breast cancer to search for genes with an enrichment of FI SNPs in their CRRs. We ran HiFIseek using promoter and ABC CRRs together with CADD, FATHMM-XF, and Sei scores. Focusing on the genes showing enrichment of FI CRVs in at least two CRR-score combinations resulted in 23 genes, 3 of which have been previously associated with cancer. Given the limited overlap, we examined each combination in more detail. We observed that FATHMM-XF scores combined with ABC CRRs resulted in an enrichment of genes associated with pan-gynecological and breast cancer using the network of cancer genes ontology (Fig AL in S1 Appendix). Based on this, we decided further to explore this combination for potential SNPs of interest. We identified 99 genes exhibiting enrichment of FI SNPs (adjusted p-value ≤ 0.05), including 18 that have been previously reported to be associated with cancer. We identified *BRCA1* among the detected cancer-related genes, a tumor suppressor gene whose loss of function is strongly associated with an increased risk of breast cancer. Given this established role, we next explored the SNPs falling within the CRRs of *BRCA1*. First, we plotted the observed SNP scores in the context of all possible changes in the considered regions to pinpoint those contributing the most to the enrichment (Fig AM in S1 Appendix). In the case of *BRCA1*, SNPs were observed to be at both extremes of the score distribution. Based on this, we further explored the *BRCA1* SNPs showing a FATHMM-XF score ≥ 0.75. We used the UCSC genome browser [32] to study the genomic context surrounding the selected SNPs (Fig 7). Most high-scoring SNPs were located at a genomic region showing active histone marks in HMEC and MCF7 cells. The inclusion of MCF7 cap analysis of gene expression (CAGE) data from FANTOM5 [33] revealed the presence of a lncRNA overlapping with the selected SNPs. Additionally, some of the detected SNPs have been previously reported by FANTOM5 as eQTLs for *BRCA1*.

**Fig 7:**
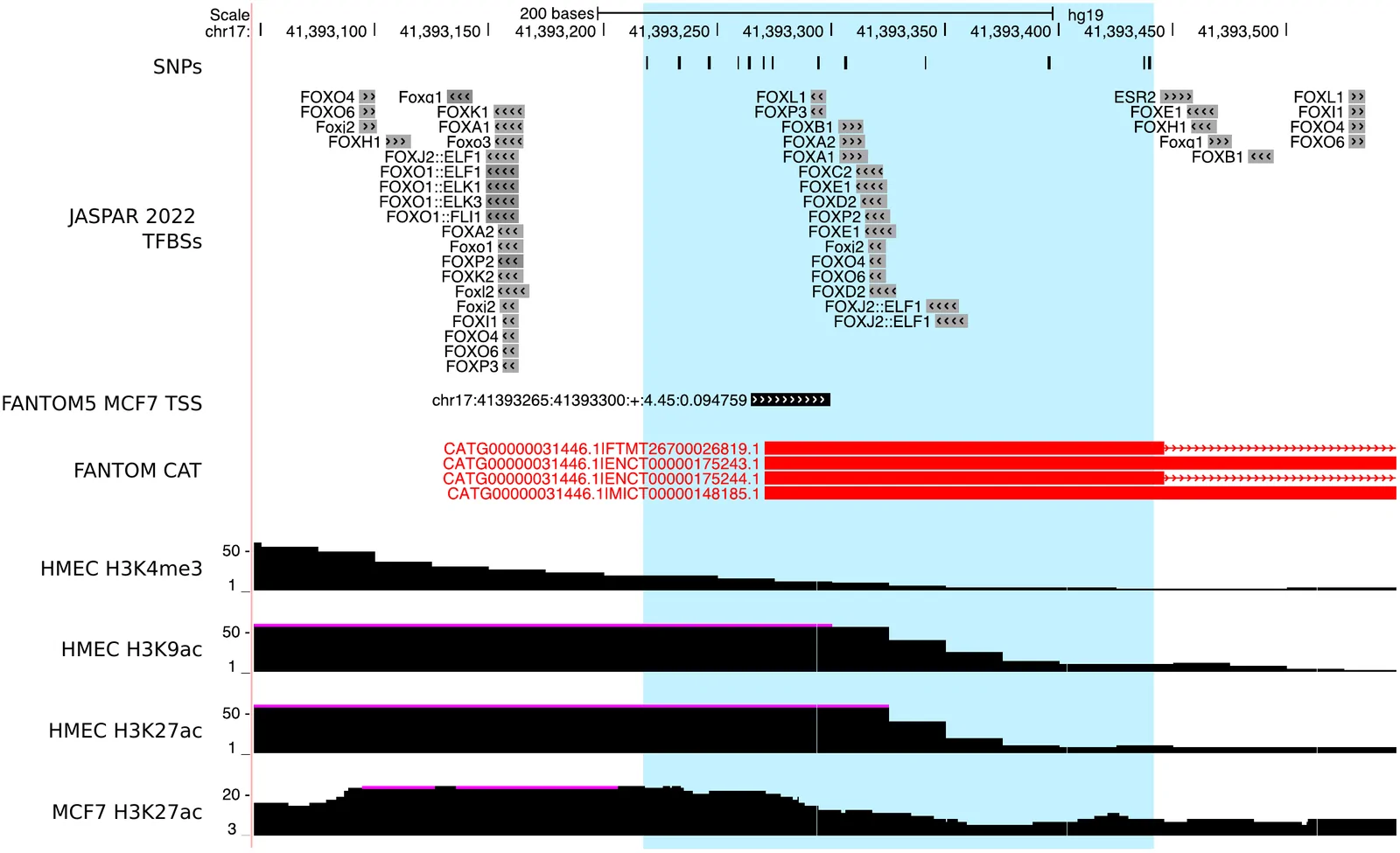
BRCA1 shows high functional impact SNPs in ABC-derived CRRs. Genomic context around the high FI score SNPs in one of the *BRCA1* CRRs. From top to bottom, tracks show observed SNPs, JASPAR 2022 TFBSs with a score higher than 300, FANTOM5 TSS predictions in MCF7 cells, FANTOM CAT transcript predictions, HMEC H3K4me3, H3K9ac and H3K27ac, and MCF7 H3K27Ac.

To further characterise the potential mechanisms of action of the 1,026 SNPs associated with the 99 genes identified by HiFIseek, we performed a multi-layered functional annotation. eQTL overlaps with GTEx breast tissue data identified 4 variants matching known eQTLs for the *ENOSF1* gene. In the TCGA breast tumour data (n = 653; White and non-Hispanic Latino ancestry) further 10 cis-eQTLs and 422 trans-eQTL hits were validated after Benjamini-Hochberg adjustment. Genes identified at the bulk level did not include known cancer drivers, with the exception of *NMBR* identified in trans. Cell type specific transcriptional effects were predicted using ExPectoSC, identifying potential function for 347 of the 1,026 SNPs (|z-score| > 1) with predicted effects on the expression of 103 genes across breast epithelial, immune, endothelial, and stromal cell types. Of these, 205 variants showed effects across all four cell types, while the remainder showed cell-type-specific or partially shared patterns. Among the 103 predicted target genes, 25 were known cancer drivers, including *PIK3CA, NF1, TRIM27, EIF2AK3*, and *MYCBP2* (Fig AN in S1 Appendix). These results suggest that the identified SNPs may act through transcriptional effects that are in part cell-type restricted. Regulatory region annotation via VEP found 69.8% of variants (n = 717) in at least one known regulatory region. Because variants could be assigned to multiple regulatory categories, annotations were not mutually exclusive. The majority of annotated variants overlapped enhancers (n = 521), followed by promoters (n = 193), with a smaller proportion of variants overlapping CTCF binding sites (n = 9) and open chromatin regions (n = 2), suggesting that a subset of the identified risk SNPs may affect transcriptional regulation. Splicing effects were predicted for 9 variants using SpliceAI, MaxEntScan, and dbscSNV, mapping to 8 genes, including the drivers *MUTYH* and *PPIG*. Together, the identified prediction results provide multi-layered regulatory evidence supporting the potential functional relevance of identified variants, even in the absence of direct overlaps between layers.

## Discussion

Searching for potential cis-regulatory driver events is a challenging endeavor due to the high complexity of transcriptional regulation. While the basic underlying mechanism is understood, fine-grained, context-specific knowledge of transcriptional regulation remains largely unknown. For example, the same gene may use very different cis-regulatory spaces depending on its cell type and/or cell state and condition. The same variant may have very different effects in different cellular contexts. This leads to heterogeneity in the input data as well as the underlying assumptions of the applied methods. Because of this, the existing broad range of gene-CRR associations and variant impact scoring methods, albeit representing similar mechanisms, yield very little consensus across association or scoring methods. The lack of high-quality annotations makes the task of identifying candidate non-protein-coding driver events even more challenging.

In this work, we describe HiFIseek, a Snakemake pipeline studying the enrichment of high-impact variants in sets of related genomic regions using any number of cohorts, gene-region associations, and FI scoring methods. We introduce OncodriveFML-analytic, an analytic approach to the OncodriveFML framework. The main differences between OncodriveFML and our analytic counterpart reside in how the functional impact scores are aggregated and the use of an analytical approach to compute significance. OncodriveFML-analytic demonstrated higher computational performance compared to OncodriveFML, particularly when applied to region associations of increasing sizes. Comparison of OncodriveFML and OncodriveFML-analytic showed similar results across different analyses, with increasing discrepancy when using larger region associations. We hypothesize that these discrepancies are mainly due to the minimal number of required permutations as the regions studied become larger.

A challenge in detecting gene regions with a higher-than-expected aggregated impact score is striking a balance between a method’s sensitivity and specificity. An important factor in this is the ratio between low and high-scoring variants in the studied regions. For example, a region set showing many low-impact variants and a few high-scoring ones may not be detected, decreasing the method’s sensitivity. While this could be tackled by pre-filtering very low-scoring mutations, doing so is likely to introduce many false positives and therefore decrease the method’s specificity.

While OncodriveFML-analytic detected many known cancer-related genes when applied to somatic mutations in protein-coding regions, most genes with an enrichment of high-impact CRVs have not been previously recorded to be cancer drivers. In CRRs, the amount of disagreement across association and scoring approaches was larger than in the protein-coding space. These results agree with the observed differences between association and FI scoring methods. Additionally, we did not observe significant changes in the transcription levels of the detected genes. This could be a consequence of cell-type specificity, which we investigated using ExPectoSC for the breast cancer SNPs, and incomplete coverage of current CRR and FI annotations discussed above. Importantly, variants may act only in cell subpopulations diluted in bulk RNA-seq data or may affect regulatory mechanisms not reflected in overall expression levels, such as alternative splicing, isoform usage, or chromatin state. Future work integrating cell-type-resolved CRR-gene associations with FI scoring, possibly combined with information from single-cell expression and chromatin opening, perturbation-based assays such as CRISPR screens, and tissue- or cell-type-matched QTL data will be critical for refining inputs and validating the predictions for computational models.

We used HiFIseek to explore gene CRRs using a set of SNPs associated with increased breast cancer risk. While we did not observe a strong consensus across CRR-score combinations, the use of FATHMM-XF scores with ABC CRRs resulted in 18 cancer-related genes with FI SNPs in their CRRs. Among these genes, we identified *BRCA1*, whose role in breast cancer is well-established. Nevertheless, these results were only supported by one of the explored CRR-score combinations. Given the disagreement across association and scoring approaches, the actual significance of these results is unclear. Likewise, we did not investigate the influence of linkage disequilibrium in our analysis. We further investigated the SNPs in *BRCA1* CRRs due to their known involvement in breast cancer. Manual exploration revealed a subset of SNPs that clustered in a genomic region with signs of activity in both normal and breast cancer cell lines. Given that some of the detected SNPs have been reported as eQTLs for *BRCA1*, a potential hypothesis is that they increase breast cancer risk by altering *BRCA1* transcription in normal cells. However, the alignment of most high-impact SNPs with an actively transcribed lncRNA in MCF7 cells raises the question of how directly they may influence *BRCA1* transcription. Further work is required to disentangle the involvement of these SNPs in increasing breast cancer risk. Similarly, fine-mapping studies can help identify the most likely causal SNPs.

The functional annotation of risk SNPs identified by HiFIseek revealed multi-layered regulatory mechanisms of action. Although cis-eQTL overlap was limited, trans-eQTL analysis implicated potential long-range regulatory roles, revealing the implication of many novel genes, *NMBR* being the only cancer driver identified at bulk. Cell-type-resolved predictions from ExPectoSC provided broader functional evidence, implicating 25 known cancer drivers, including *PIK3CA, NF1*, and *MYCBP2*, across breast epithelial, immune, endothelial, and stromal cell types. Splicing effects predicted for 9 variants, mapping to drivers including *MUTYH* and *PPIG*, represent an additional regulatory layer. Together, these findings demonstrate the complexity of non-coding risk variant mechanisms and the value of integrating cell-type-resolved and chromatin-level evidence for interpretation. Limitations of current CRR and FI scoring annotations, including incomplete cell-type coverage and the absence of isoform-level and chromatin accessibility data such as ATAC-seq are likely to contribute to the incomplete functional picture and represent important aspects for future investigation.

Overall, these results illustrate the challenge in characterising non-coding variant function and highlight the multi-faceted mechanisms underlying risk SNP activity that are unlikely to be captured by any single annotation approach. This is consistent with the broader disagreement across association and scoring methods observed throughout this work and underscores the potential of multi-layered computational frameworks such as HiFIseek in addressing this challenge.

## Materials and methods

### Cancer data

We used the icgc-get client from the International Cancer Genome Consortium (ICGC) [25] to download mutation, transcription, and copy number alteration data from different TCGA cohorts [24]. We used those cohorts for which we could find at least 30 samples with whole genome sequencing (WGS) and RNA-seq data. We downloaded small single nucleotide variants (SNVs) called by MuSE, resulting in 344 samples in total for the following cohorts: BRCA-US (breast invasive carcinoma - 89 donors), LIHC-US (liver hepatocellular carcinoma - 50 donors), UCEC-US (uterine corpus endometrial carcinoma - 48 donors), HNSC-US (head and neck squamous cell carcinoma - 43 donors), LUSC-US (lung squamous cell carcinoma - 42 donors), LUAD-US (lung adenocarcinoma - 37 donors), and STAD-US (stomach adenocarcinoma - 35 donors)(Fig AO in S1 Appendix). Additionally, we obtained 560 WGS samples from the ICGC breast cancer cohort.

We filtered out hypermutated samples following the approach in [34]. Very briefly, we removed samples showing at least 1000 mutations over 1.5 times the interquartile range above the 75th percentile. After filtering, we ended up with the following sample numbers per cohort: ICGC breast cancer - 520, BRCA-US - 85, LIHC-US - 48, UCEC-US - 43, HNSC-US - 40, LUSC-US - 40, LUAD-US - 33, and STAD-US - 32 (Fig AO in S1 Appendix).

RNA-seq raw counts were pre-filtered to remove genes showing 0 reads in 50% of the samples in a cohort. We then normalised raw counts to counts per million and applied a log2 conversion.

### SNPs data

We used observed and imputed genotypes from the Norwegian Breast Cancer Study (NBCS), based on 2,955 individuals, including 2,679 sporadic female breast cancer cases and 276 healthy controls. Samples were genotyped using the iCOGs [26] and OncoArray [35] custom SNP arrays. Imputation was performed as part of the Breast Cancer Association Consortium (BCAC) [26]. Participants were recruited between 1975 and 2017 from Oslo University Hospital, Norwegian Radium Hospital, Rikshospitalet, Vestre Viken Hospital Trust, and Akershus University Hospital.

### Gene-region associations

We utilized the following gene-region associations: RefSeq curated annotations from the UCSC Table Browser, ABC-derived associations, Epiregio, GeneHancer, and superenhancers from SEdb.

The RefSeq curated set of annotations was downloaded on March 28 2022 from the UCSC Table Browser (ncbiRefSeqCurated table) and was used to obtain the following annotations for each gene: (1) 1000 bp sequences upstream of their TSS, which we refer to as promoter regions; (2) 5’ UTRs; (3) coding sequences; (4) intronic regions; and (5) 3’ UTRs. Additionally, we used *bedtools subtract* (version 2.30) to remove any promoter sequences overlapping with neighboring gene bodies, thereby eliminating potential confounding effects. Finally, we translated RefSeq transcript IDs to Entrez gene IDs and used the *bedtools merge* function to merge overlapping genomic regions corresponding to the same gene, thereby avoiding duplicated genomic windows.

ABC associations were downloaded on Feb 9 2022 from ftp://ftp.broadinstitute.org/outgoing/lincRNA/ABC/AllPredictions.AvgHiC.ABC0.015.minus150.ForABCPaperV3.txt.gz and were further processed to fit the required format for OncodriveFML and HiFIseek. We converted gene symbols to Entrez IDs and subtracted regions overlapping with exonic sequences. Finally, we created cohort-specific ABC region subsets by selecting gene-region associations derived from cell types similar to the affected types in a given cohort. Briefly, breast cancer samples used regions annotated as mammary_epithelial_cell-Roadmap, liver hepatocellular carcinoma samples used regions annotated as liver-ENCODE, lung carcinomas used regions annotated as IMR90-Roadmap, stomach adenocarcinoma used regions annotated under stomach-Roadmap, and uterine corpus endometrial carcinoma used regions annotated as uterus-ENCODE.

Epiregio regions were obtained from https://zenodo.org/record/3758494/files/REMAnnotationModelScore_1.csv.gz on July 12 2022. We selected those regions showing a p-value ≤ 0.05, converted Ensembl IDs to Entrez IDs, lifted them to hg19 using the UCSC liftOver tool [32], merged overlapping intervals associated to the same gene, and subtracted regions overlapping with exonic sequences.

GeneHancer associations (version 4.9) were obtained on February 21, 2019. Processing of these regions included selection of elite enhancers, conversion of gene symbols to Entrez IDs, lifting to hg19, merging overlapping intervals for regions associated with the same gene, and subtracting exonic regions.

### Scoring methods

The following functional impact scoring methods were used in this work: CADD (v1.6, obtained on Mar 5 2021) [18], DANN (obtained on Feb 1 2018) [19], EIGEN (obtained on Feb 2 2018) [21], EIGEN-PC (obtained on Feb 4 2018) [21], FATHMM-XF (coding and non-coding) (obtained on May 30 2022 and Feb 23 2018, respectively) [36], LINSIGHT (obtained on Feb 3 2018) [20], Sei (obtained on Aug 3 2022) [22], and phastCons100way (obtained on Jul 18 2022) [37].

### Defining cancer driver genes

We built a list of cancer genes by including all unique instances of genes being in any of the three following resources: (i) intogen [38]; (ii) network of cancer genes [39]; and (iii) cancer gene census [40]. Intogen genes were obtained from https://www.intogen.org/download on the 24th of Aug 2021. Cancer genes from the network of cancer genes were obtained from http://network-cancer-genes.org/download.php on 18th Oct 2023. Finally, tier 1 and 2 cancer genes from the Cancer Gene Census (v98) were obtained from https://cancer.sanger.ac.uk/cosmic/download on October 18, 2023. The resulting list of cancer genes is used throughout this work to study the number of cancer genes in the list of genes showing an enrichment of FI mutations in their associated regions.

### Descriptive statistics

#### Number of mutations by region type.

We intersected the RefSeq annotations (see the Gene-region associations section in Methods) with the filtered mutation sets to determine the number of mutations mapping to each type of region. For each cohort, we used *bedtools intersect* and the *uniq* command to determine the number of unique mutations that intersected with each region set in the RefSeq annotations. Additionally, we annotated as “Other non-coding” all mutations that did not overlap with any of the RefSeq-annotated regions.

#### Mutation rates in cancer versus non-cancer genes.

For each cohort and gene-CRR association, we computed mutation rates as the number of nucleotides showing at least one mutation in a gene’s CRRs divided by the total number of nucleotides in the gene’s CRRs. Genes were separated into cancer and non-cancer genes using annotations from the Cancer Gene Census. Plots comparing the mutation rates were generated with R [41] using the ggplot2 [42] and ggpubr [43] packages.

#### Distribution of functional impact scores in cancer versus non-cancer genes.

We obtained scores for the mutations falling within each gene’s CRRs for each cohort, gene-region association, and mutation impact scoring method. Genes were then categorised as cancer and non-cancer genes using annotations from the Cancer Gene Census. Plots comparing the distribution of impact scores in cancer versus non-cancer genes were generated with R using the ggplot2 and ggpubr packages.

#### Gene-CRR sizes and overlap.

For each gene, we obtained its respective CRRs according to ABC, epiregio, Genehancer, and promoters. We also introduced intronic regions to explore the number of gene CRRs predicted to fall within introns. We next computed the number of nucleotides in the entire space of a gene’s CRRs for each association type and plotted the distribution of gene-CRR sizes using the R ggplot2 and ggridges [44] packages.

Gene-CRR overlaps across association methods were computed using bedtools intersect. More specifically, we computed for each gene the number of intersecting nucleotides between all possible CRR pairwise comparisons. Due to the asymmetry of genomic region intersection, the proportion of intersecting nucleotides was computed in relation to one of the CRR methods in the comparison. For example, the overlap proportion of nucleotides in association method *A* intersecting with association method *B* for gene *g* was computed as:


$$\begin{array}{c} \frac{A_{g}\cap B_{g}}{N_{nucleotides\in A_{g}}} \end{array}$$

where $A_{g}\cap B_{g}$ is the number of nucleotides in association method *A* intersecting with association method *B* for gene *g* and $N_{nucleotides\in A_{g}}$ is the number of nucleotides in association method *A* for gene *g*. Finally, we computed for each pairwise comparison the median overlap across all genes and plotted them using the R *gg*plot2 package.

#### Distribution of impact scores and computation of score agreement.

For each scoring method, we recorded the number of times each value was observed and plotted them using the R ggplot2 package. Agreement across scoring approaches was done by randomly sampling 10,000 distinct mutations from all TCGA cohorts and obtaining their functional impact scores for all considered scoring methods. Finally, we plotted all possible scoring method pairwise comparisons using the R ggplot2 and GGally [45] packages.

### HiFIseek

HiFIseek is a Snakemake-based pipeline computing the enrichment of functional mutations in any number of cohorts, region association, and score combinations. Enrichment is computed through two different enrichment methods: (i) OncodriveFML, and (ii) an analytical approach to the OncodriveFML framework using the sum of the observed impact scores.

HiFIseek takes as inputs one or more sets of associated genomic regions (e.g. CRRs for each gene, or coding sequences for a gene), precomputed functional impact scores, and cohorts. The results of the pipeline are output in a Markdown document to facilitate the exploration of all combinations.

The HiFIseek code can be found at https://bitbucket.org/CBGR/hifiseek.

### Enrichment computation with OncodriveFML-analytic

As a first step, OncodriveFML-analytic takes for each region the mutations intersecting with it and sums their functional impact scores. As a parameter, a user can specify the minimal number of mutations a set of regions must show to be considered in the analysis. Next, it computes the distribution of all possible sums of scores for mutation sets of the same size while taking into consideration all possible mutations in the studied region set and the mutational signature of the studied samples. First, the probability of observing each score is modeled through a multinomial distribution where each class has a functional impact score and the probability of observing it is computed considering: (i) the number of times it is observed, and (ii) the mutational signature of a given cohort. We use the *bgsignature* python package to compute the signature for a given cancer cohort. For each region set, we next obtain the signature probability and normalise it by dividing it by the sum of probabilities for all mutations in the studied region. Normalised probabilities are then aggregated by score value. We then model the distribution of score sums for an observed mutation set of size *M* as a normal distribution N(μ,σ), where:


$$\begin{array}{c} \mu =M\times E(X) \end{array}$$

$$\begin{array}{c} \sigma =\sqrt{M}\times SD(X) \end{array}$$

where E(X) and SD(X) are the mean and standard deviation of the multinomial distribution for the studied region set. Significance for the observed combined score is then calculated by computing the probability of observing an equal or higher aggregated score than the observed score. Finally, multiple testing correction is applied to all the region sets studied using the Benjamini-Hochberg method.

### Computational benchmark

We analysed the set of filtered somatic mutations in the BRCA-US cohort using CADD scores and the following gene-region associations: promoters, coding exons, and introns. The regions were selected based on the distribution of region sizes to explore the computational impact of exploring larger genomic windows. We ran each analysis using different numbers of cores to observe how the method scales as more resources can be used in parallel. All OncodriveFML analyses were run using 1,000,000 permutations. For each analysis, we recorded the total elapsed time and aggregated RAM usage across all processes. Plots comparing the results between OncodriveFML and OncodriveFML-analytic show the raw p-values reported by either OncodriveFML or OncodriveFML-analytic. Plots were generated using the ggplot2 R package.

### Gene set enrichment analysis

For each cancer cohort, we performed a gene set enrichment analysis on the genes predicted by HiFIseek (adjusted p-value < 0.05) across all combinations of CRR-gene associations and FI scoring method. The enrichment analyses were performed using Gene Ontology Biological Process (GOBP) terms with g:Profiler [46]. We considered terms with a false discovery rate (FDR) < 0.05 as enriched.

To reduce redundancy, we first performed ancestor pruning using the GO DAG parsed with goatools [47]. Specifically, when two significant terms were related and one was an ancestor of another, we retained only the descendant. Next, terms were ordered by ascending p-values and processed sequentially; a term was retained only if the Jaccard similarity between its set of intersecting genes and that of every already retained term was below 0.5, otherwise it was discarded as redundant with a more significant representative. We used Anthropic Claude to help implementing the code for this gene set enrichment analysis and for generating Fig 6; we reviewed the corresponding code.

### Functional annotation of breast cancer risk SNPs

Risk variants identified by HiFIseek, showing enrichment for high-impact non-protein-coding variants were explored for alternative mechanisms of action using several complementary datasets and *in silico* tools.

Variant-eQTL overlap was first assessed using GTEx (v7) [48] breast mammary tissue data. For broader validation, cis- and trans-eQTL analyses were performed using TCGA breast tumour data (n = 653 individuals of White and non-Hispanic Latino ancestry; excluding metastatic and solid tissue normal samples), with significance assessed after Benjamini-Hochberg correction.

Transcriptional cell-type-specific effects were further assessed using ExPectoSC [49], a deep-learning pipeline that predicts cell-type-specific gene expression from sequence. Here, the pipeline was trained on single-cell RNA-seq data from a breast tissue model [50], with predictions evaluated across four breast-relevant cell types: epithelial, immune, endothelial, and stromal. Variants with a predicted |z-score| > 1 were considered to have a transcriptional effect.

Variant consequences were annotated using the Ensembl Variant Effect Predictor (VEP) [51]. Regulatory region overlap was assessed for enhancers, promoters, CTCF binding sites and open chromatin regions.

Splicing effects were also assessed by three in silico tools: SpliceAI [52] (delta score ≥0.5 for any of the four splice site features: acceptor gain, acceptor loss, donor gain, and donor loss), dbscSV [53] (ada_score ≥0.9 and rf_score ≥0.9) and MaxEntScan [54,55] (|MaxEntScan_diff| ≥ 2).

## Supporting information

S1 Appendix Figs A–Z, AA–AO and Table A. (PDF)
