# Supplementary material for: HiFIseek: Gene-specific enrichment of high-impact mutations in associated genomic regions
Source: PLoS Comput Biol. 2026 Oct 6;22(10):e1014842. doi: 10.1371/journal.pcbi.1014842 (PMC13657309; doi:10.1371/journal.pcbi.1014842)
Supplement: Supplementary file 1 [file pcbi.1014842.s001.pdf]

Supplementary Material

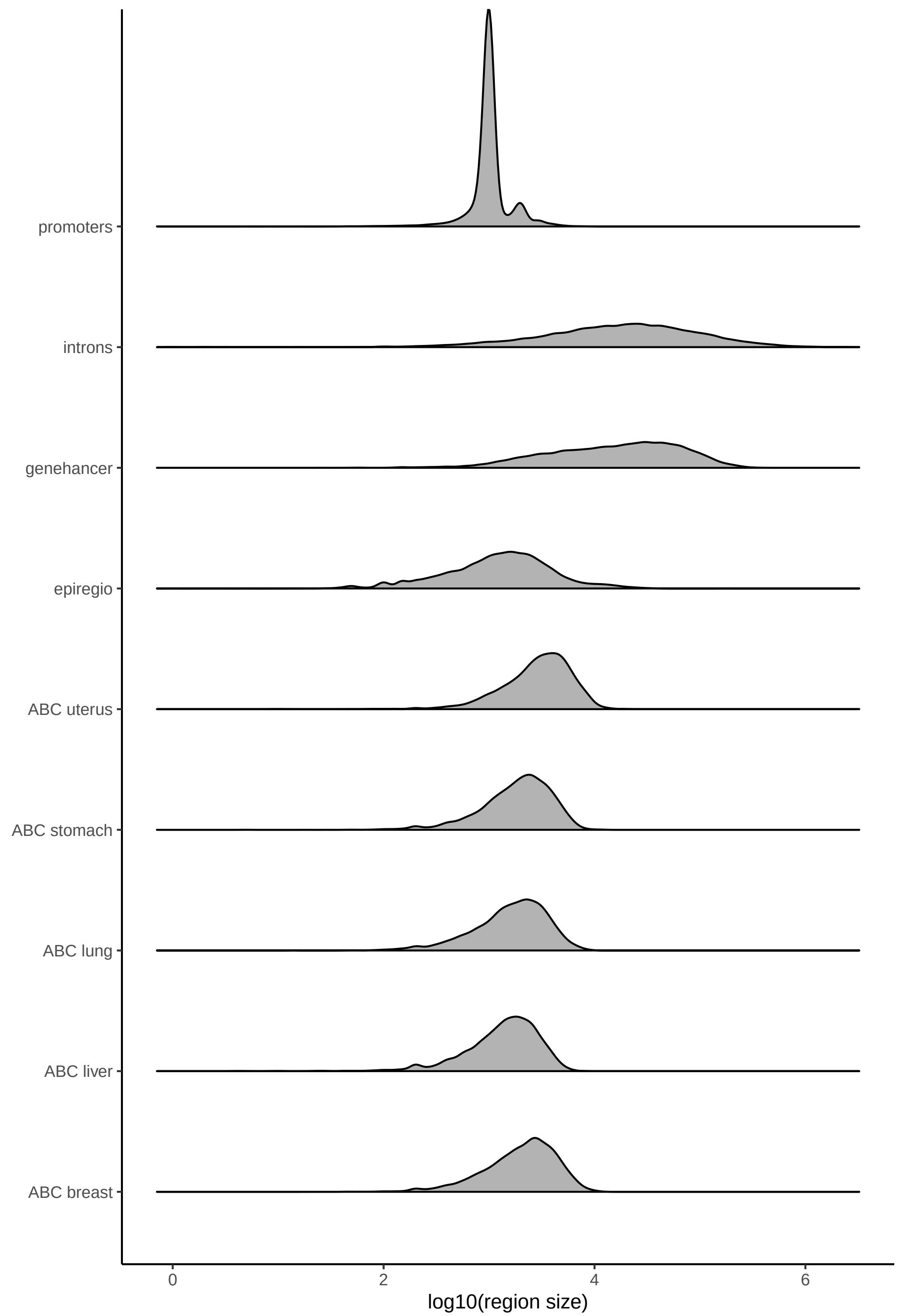

Fig A: Distribution of gene-region sizes across association methods. Density plots showing the $\log_{10}$(aggregated region size) for each gene (x-axis) according to different gene-CRR association methods (y-axis).

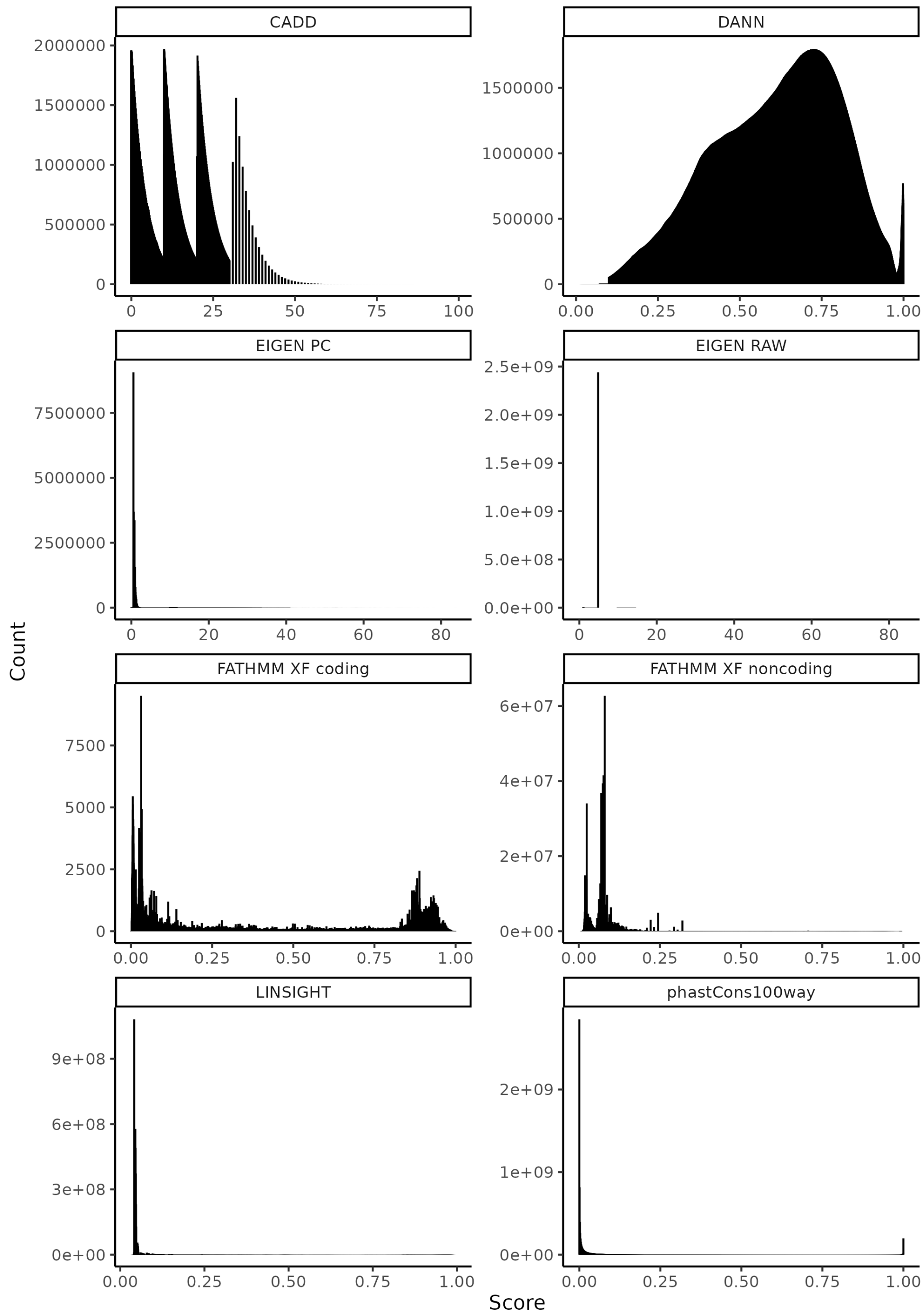

Fig B: Mutation impact scores show different distributions. Barplots showing the frequency (y-axis) of each impact score (x-axis) separated by scoring methods.

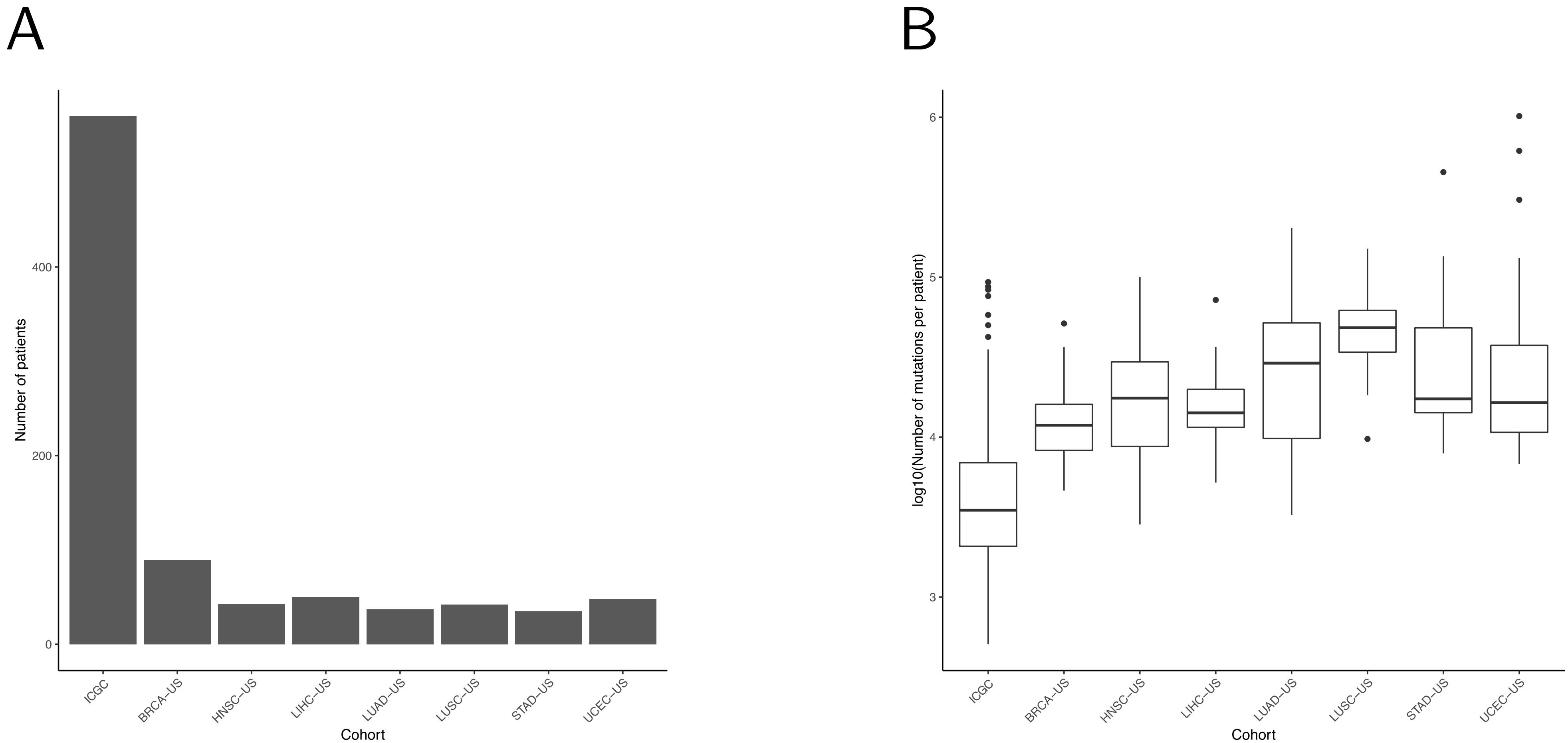

Fig C: Descriptive statistics of the used cohorts. (A) Number of patients (y-axis) per cohort (x-axis). (B) $\log_{10}$(Number of mutations per patient) (y-axis) per cohort (x-axis).

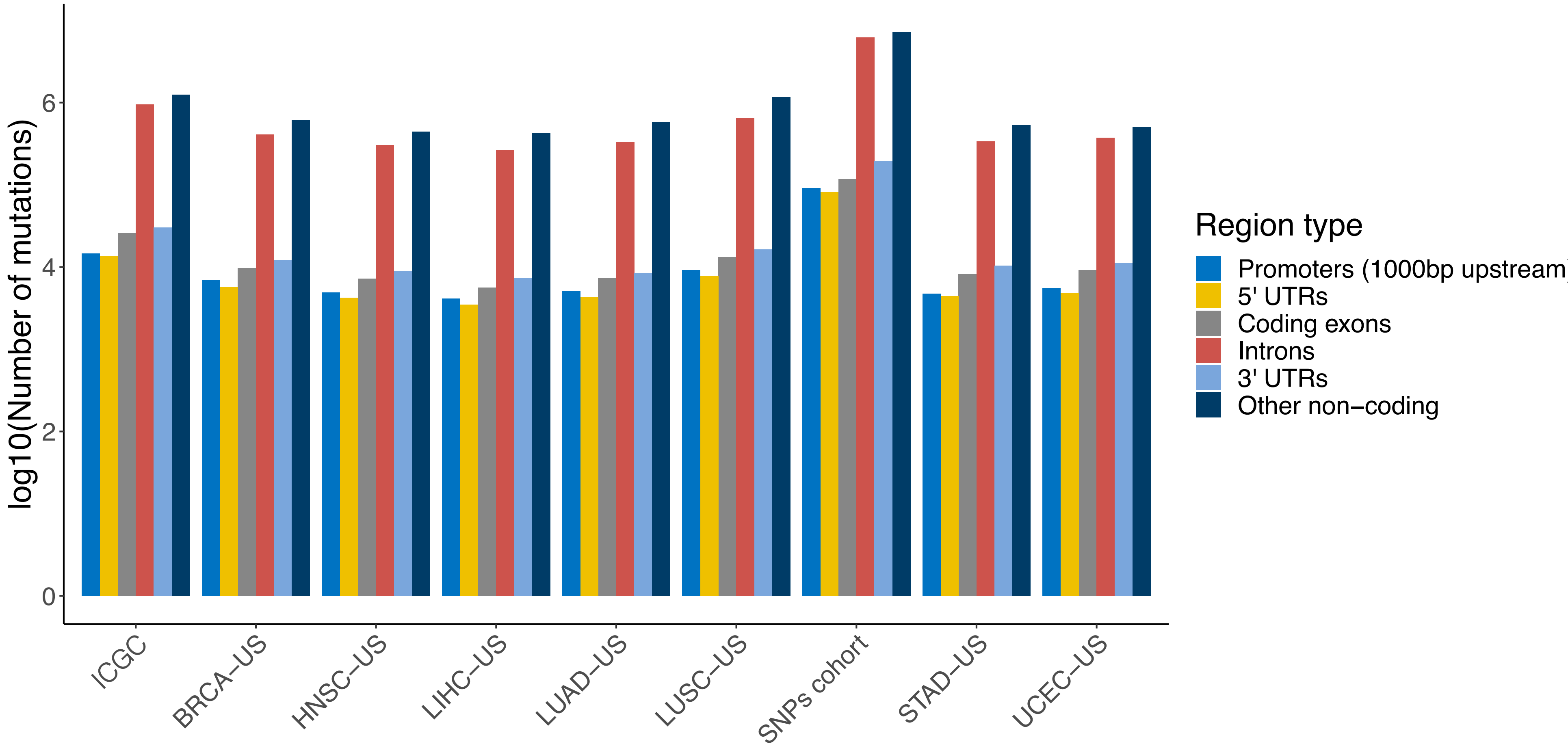

Fig D: Number of unique mutations in different types of genomic regions. Log_{10}(number of unique mutations) (y-axis) falling in promoters, 5’ UTRs, coding exons, introns, 3’ UTRs, and other non-coding regions by cancer cohort (x-axis).

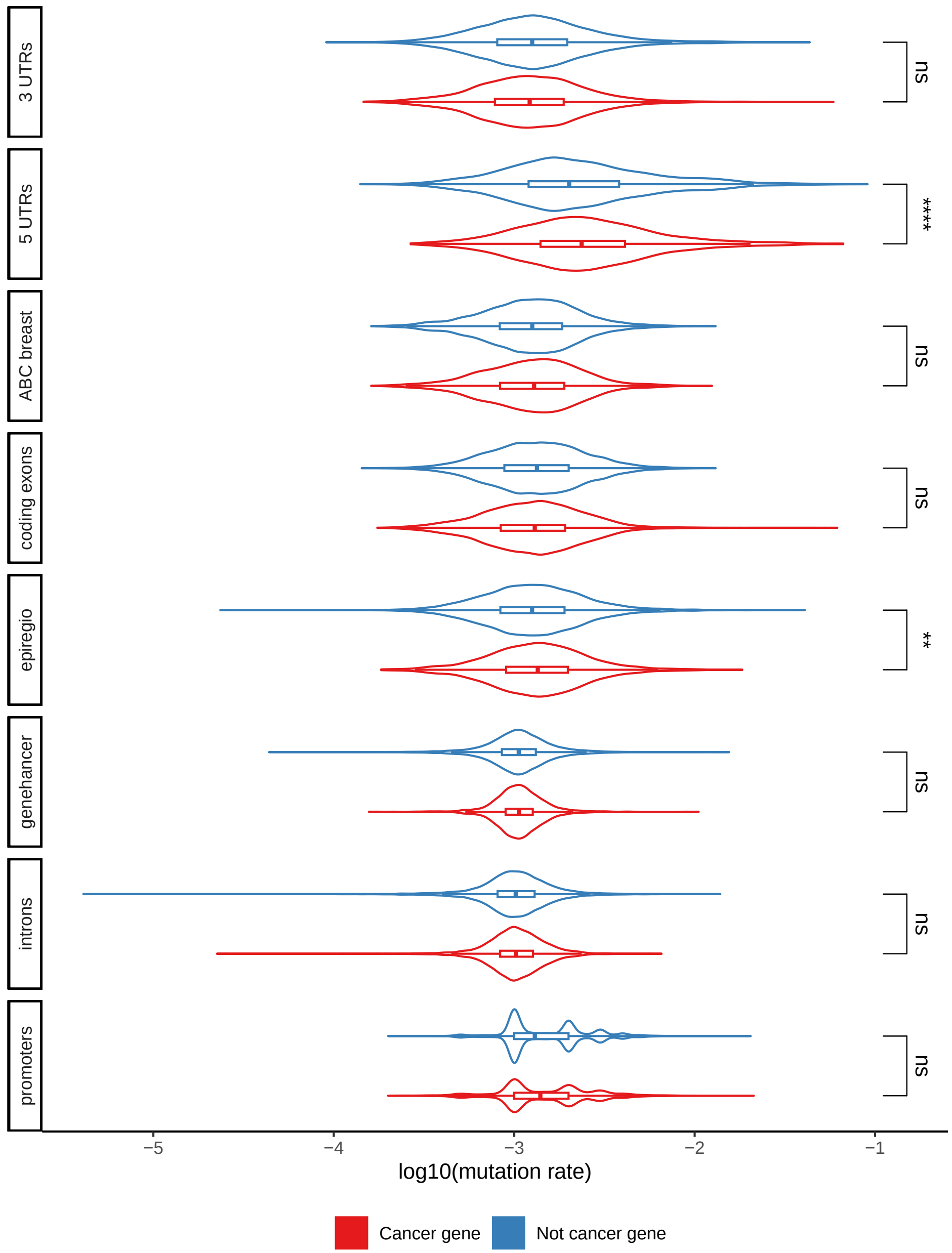

Fig E: Mutation rates per region type for the ICGC breast cancer cohort. Log_{10}(mutation rates) for cancer (red) and non-cancer (blue) genes stratified by gene-region association type. Significance labels indicate the p-value for the one-sided Wilcoxon test checking whether the $\log_{10}$(mutation rate) for the cancer genes $>$ $\log_{10}$(mutation rate) for the non-cancer genes. Significance labels indicate: ns - $p > 0.05$; $* : p \leq 0.05$; $** : p \leq 0.01$; $*** : p \leq 0.001$; $**** : p \leq 0.0001$.

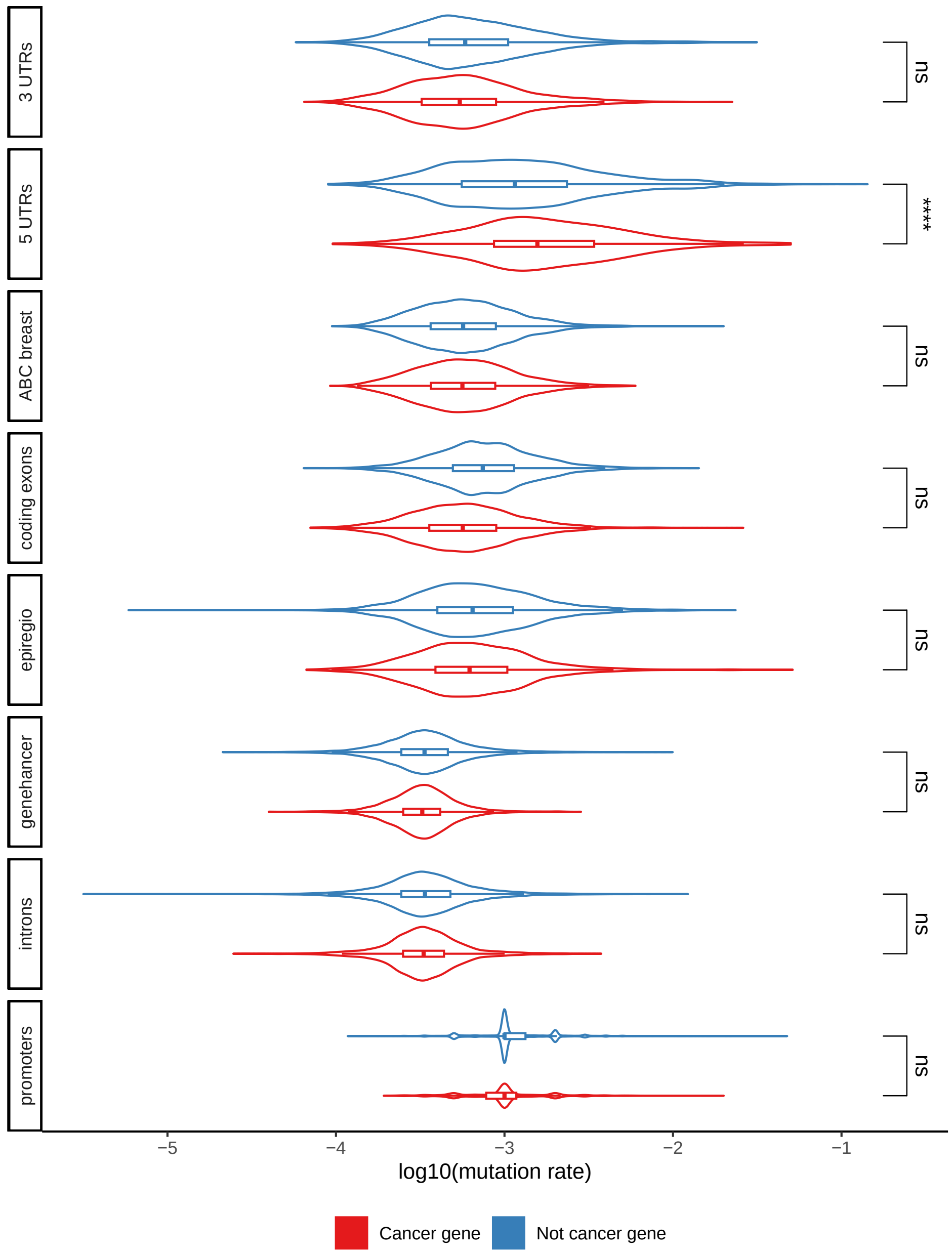

Fig F: Mutation rates per region type for the BRCA-US cohort. Log_{10}(mutation rates) for cancer (red) and non-cancer (blue) genes stratified by gene-region association type. Significance labels indicate the p-value for the one-sided Wilcoxon test checking whether the $\log_{10}$(mutation rate) for the cancer genes $>$ $\log_{10}$(mutation rate) for the non-cancer genes. Significance labels indicate: ns - $p > 0.05$; $* : p \leq 0.05$; $** : p \leq 0.01$; $*** : p \leq 0.001$; $**** : p \leq 0.0001$.

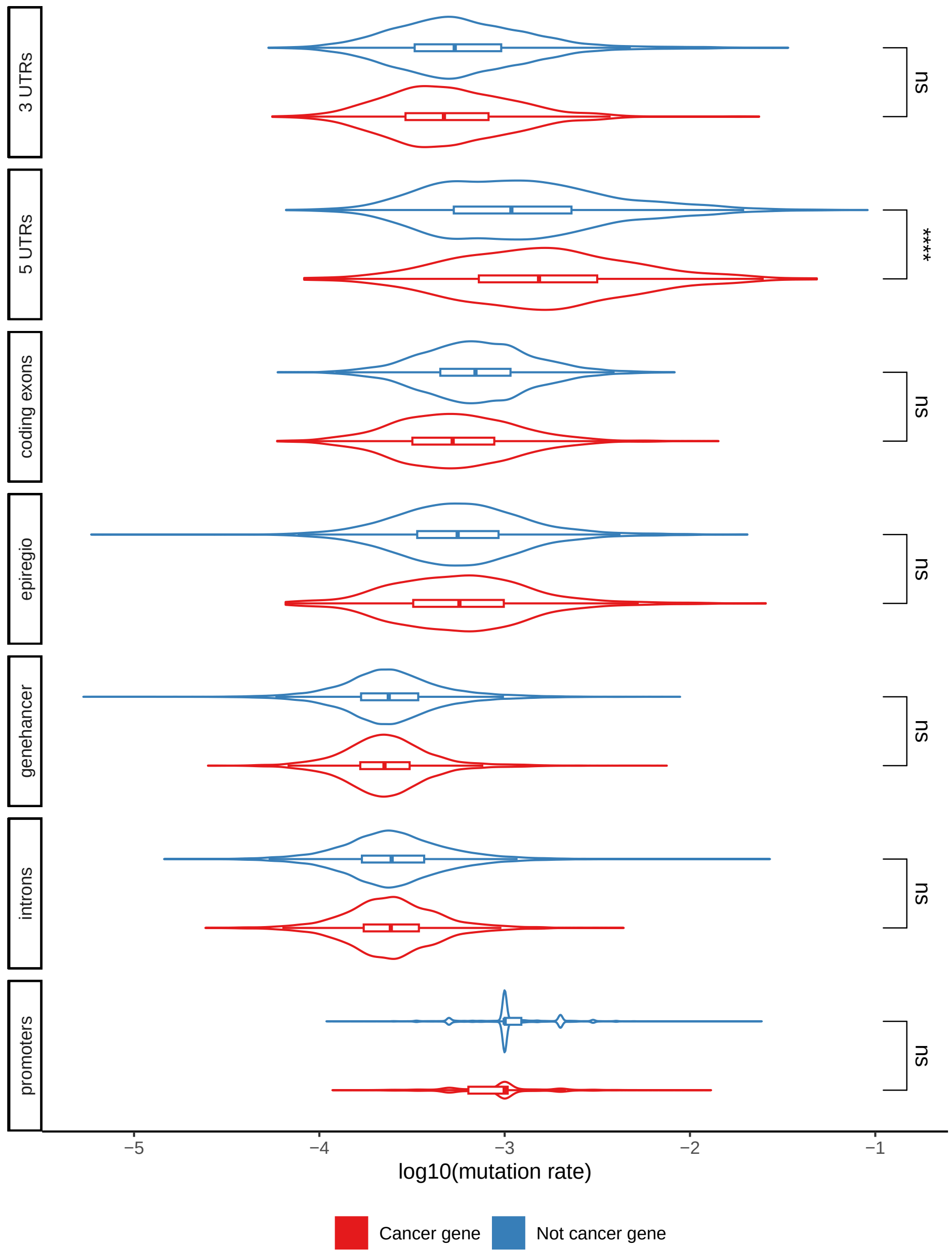

Fig G: Mutation rates per region type for the HNSC-US cohort. Log_{10}(mutation rates) for cancer (red) and non-cancer (blue) genes stratified by gene-region association type. Significance labels indicate the p-value for the one-sided Wilcoxon test checking whether the $\log_{10}$(mutation rate) for the cancer genes $>$ $\log_{10}$(mutation rate) for the non-cancer genes. Significance labels indicate: ns - $p > 0.05$; $* : p \leq 0.05$; $** : p \leq 0.01$; $*** : p \leq 0.001$; $**** : p \leq 0.0001$.

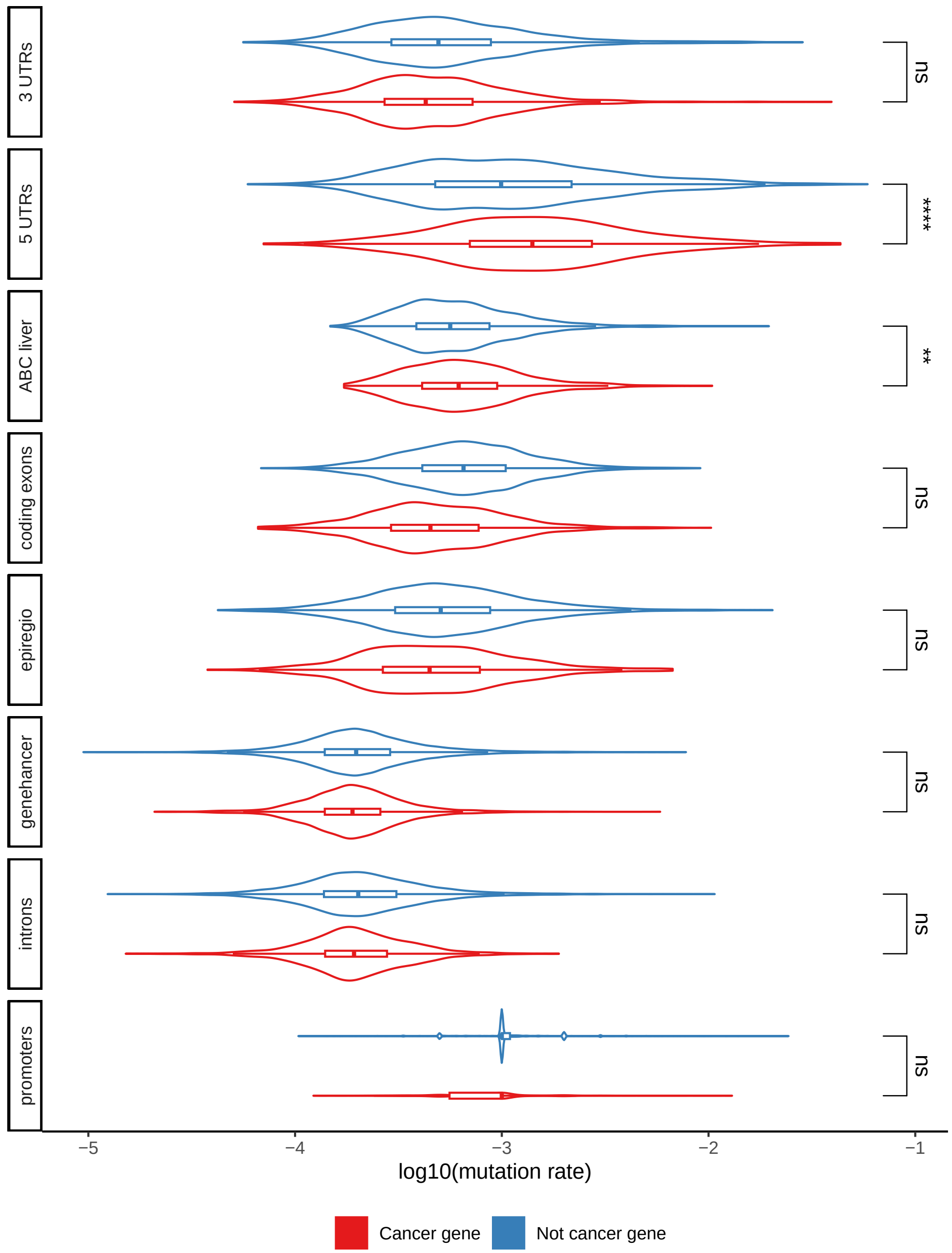

Fig H: Mutation rates per region type for the LIHC-US cohort. Log_{10}(mutation rates) for cancer (red) and non-cancer (blue) genes stratified by gene-region association type. Significance labels indicate the p-value for the one-sided Wilcoxon test checking whether the $\log_{10}$(mutation rate) for the cancer genes $>$ $\log_{10}$(mutation rate) for the non-cancer genes. Significance labels indicate: ns - $p > 0.05$; $* : p \leq 0.05$; $** : p \leq 0.01$; $*** : p \leq 0.001$; $**** : p \leq 0.0001$.

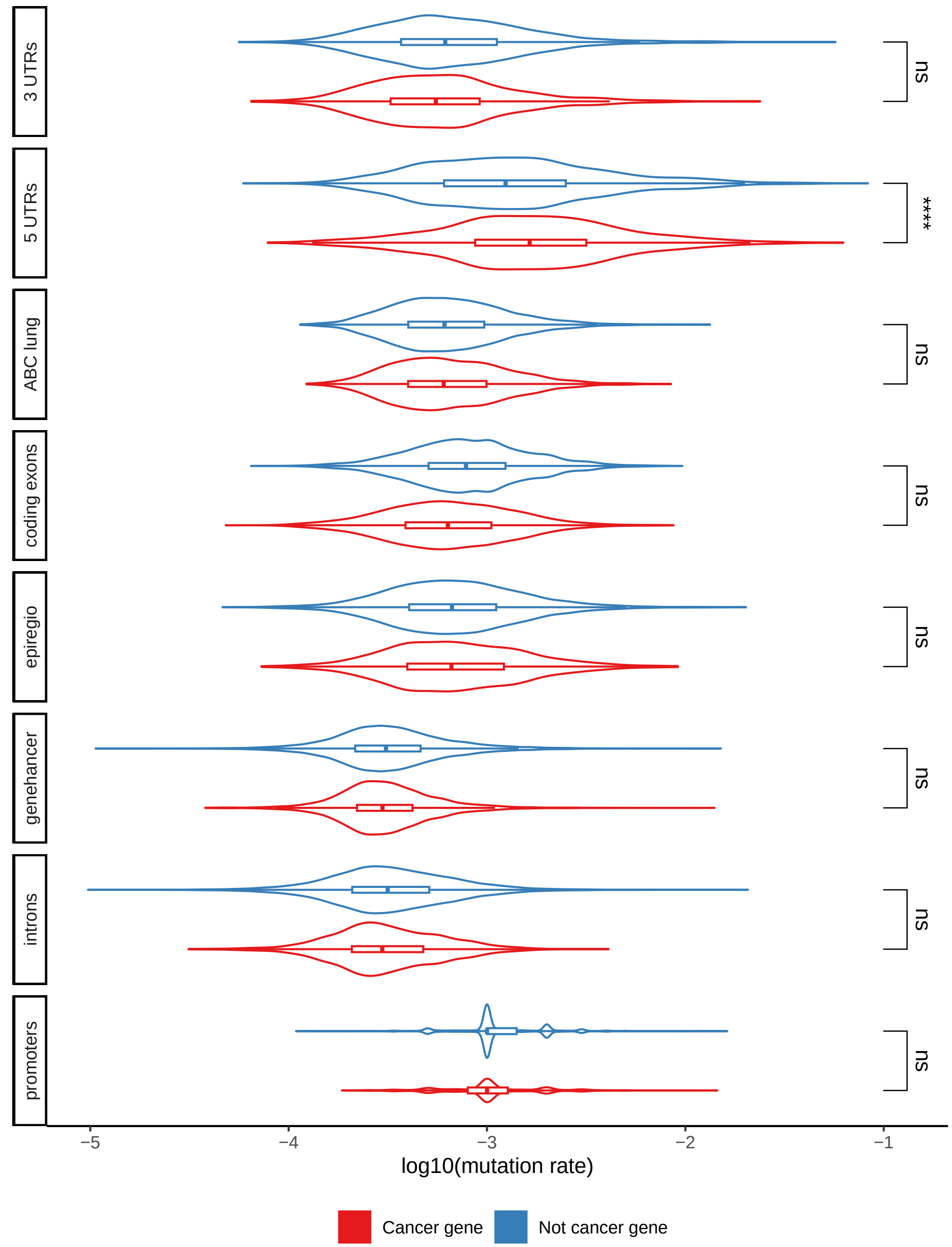

Fig I: Mutation rates per region type for the LUAD-US cohort. Log_{10}(mutation rates) for cancer (red) and non-cancer (blue) genes stratified by gene-region association type. Significance labels indicate the p-value for the one-sided Wilcoxon test checking whether the $\log_{10}$(mutation rate) for the cancer genes $>$ $\log_{10}$(mutation rate) for the non-cancer genes. Significance labels indicate: ns - $p > 0.05$; $* : p \leq 0.05$; $** : p \leq 0.01$; $*** : p \leq 0.001$; $**** : p \leq 0.0001$.

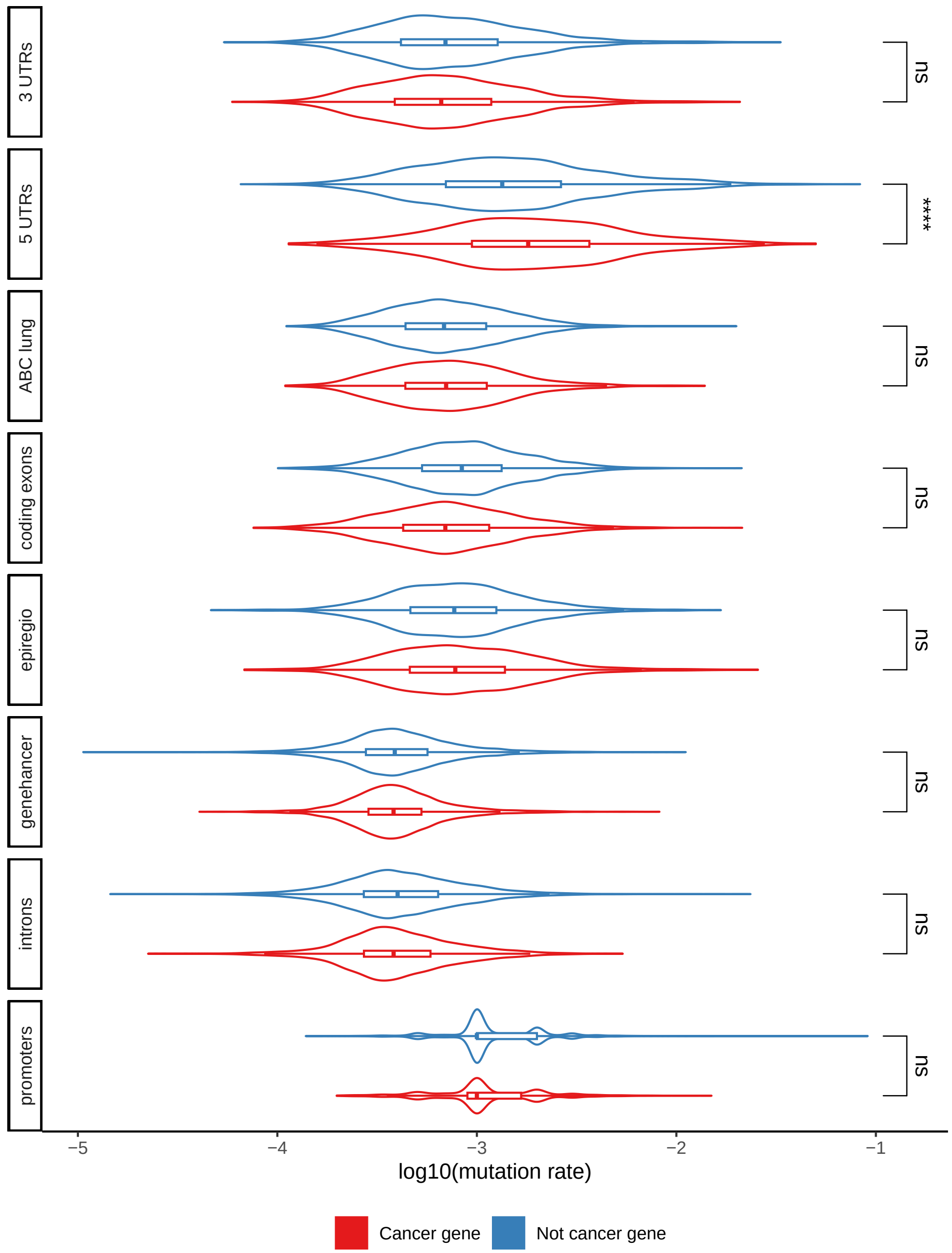

Fig J: Mutation rates per region type for the LUSC-US cohort. Log_{10}(mutation rates) for cancer (red) and non-cancer (blue) genes stratified by gene-region association type. Significance labels indicate the p-value for the one-sided Wilcoxon test checking whether the $\log_{10}$(mutation rate) for the cancer genes $>$ $\log_{10}$(mutation rate) for the non-cancer genes. Significance labels indicate: ns - $p > 0.05$; $*: p \leq 0.05$; $**: p \leq 0.01$; $***: p \leq 0.001$; $****: p \leq 0.0001$.

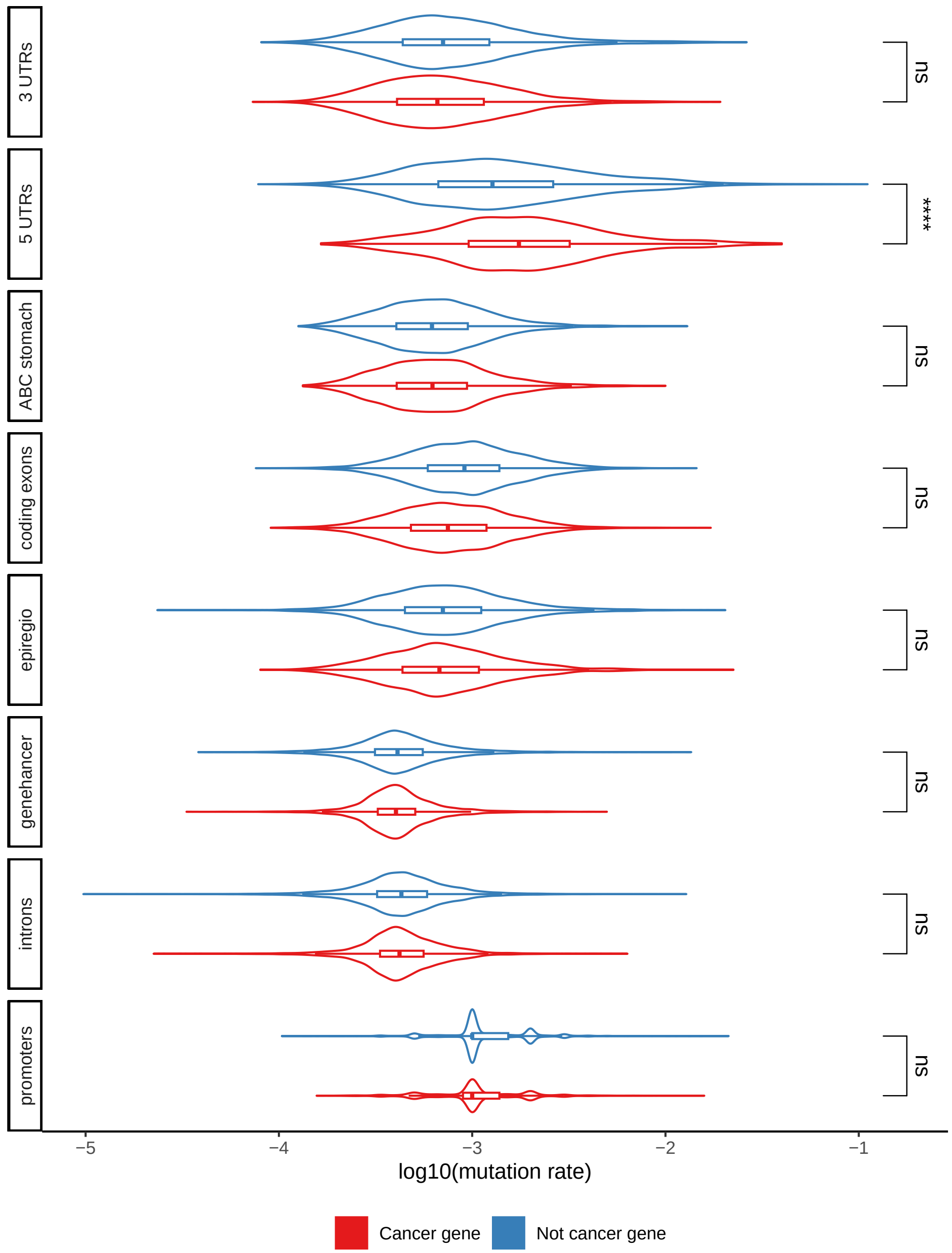

Fig K: Mutation rates per region type for the STAD-US cohort. Log_{10}(mutation rates) for cancer (red) and non-cancer (blue) genes stratified by gene-region association type. Significance labels indicate the p-value for the one-sided Wilcoxon test checking whether the $\log_{10}$(mutation rate) for the cancer genes $>$ $\log_{10}$(mutation rate) for the non-cancer genes. Significance labels indicate: ns - $p > 0.05$; $* : p \leq 0.05$; $** : p \leq 0.01$; $*** : p \leq 0.001$; $**** : p \leq 0.0001$.

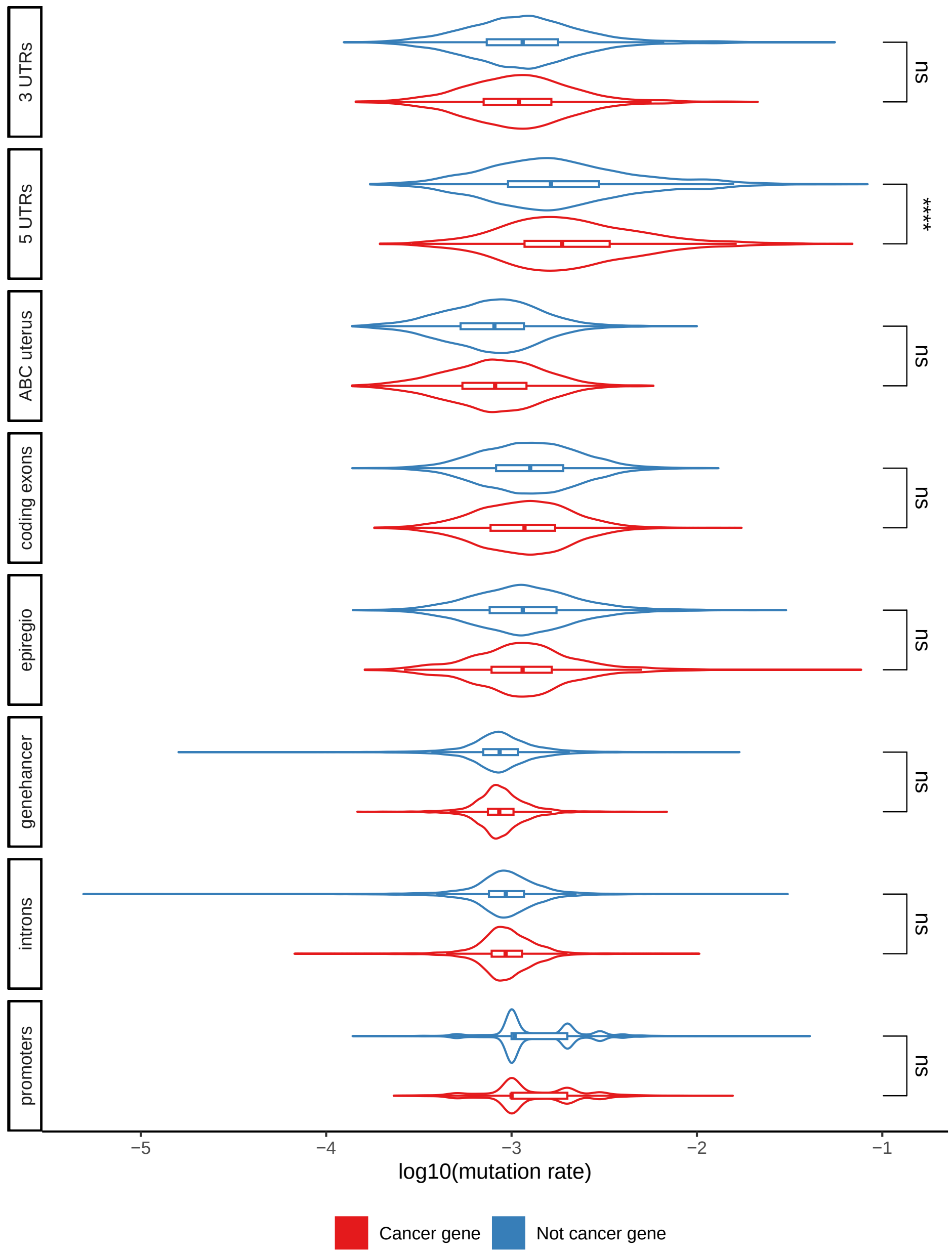

Fig L: Mutation rates per region type for the UCEC-US cohort. Log_{10}(mutation rates) for cancer (red) and non-cancer (blue) genes stratified by gene-region association type. Significance labels indicate the p-value for the one-sided Wilcoxon test checking whether the $\log_{10}$(mutation rate) for the cancer genes $>$ $\log_{10}$(mutation rate) for the non-cancer genes. Significance labels indicate: ns - $p > 0.05$; $* : p \leq 0.05$; $** : p \leq 0.01$; $*** : p \leq 0.001$; $**** : p \leq 0.0001$.

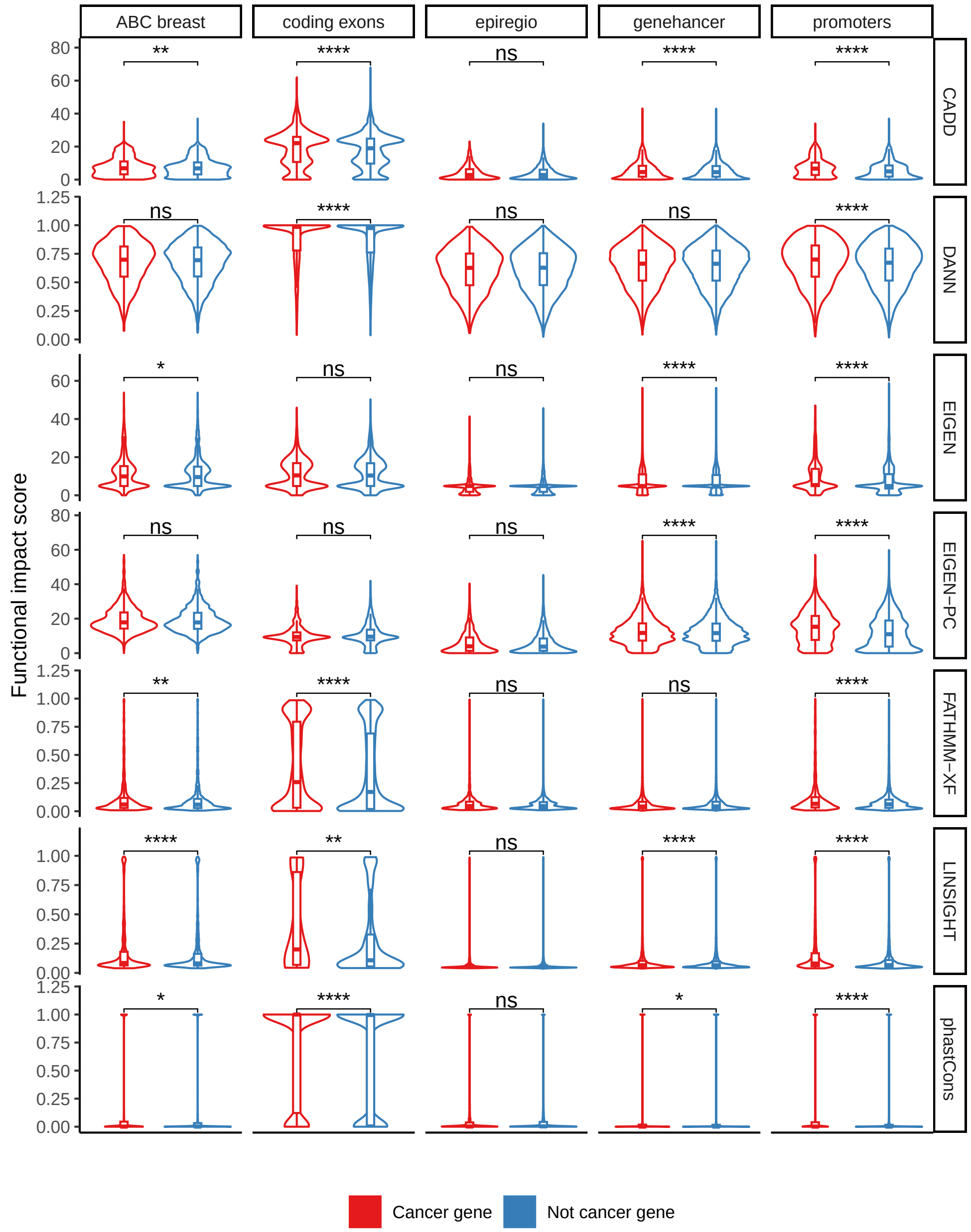

Fig M: Impact score distributions per region and score type for the observed mutations in the ICGC breast cancer cohort. Distribution of functional impact scores for cancer (red) and non-cancer (blue) genes separated by association and scoring type. Significance labels indicate the p-value for the one-sided Wilcoxon test checking whether the $\log_{10}$(mutation rate) for the cancer genes $>$ $\log_{10}$(mutation rate) for the non-cancer genes. Significance labels indicate: ns - $p > 0.05$; $*: p \leq 0.05$; $**: p \leq 0.01$; $***: p \leq 0.001$; $****: p \leq 0.0001$.

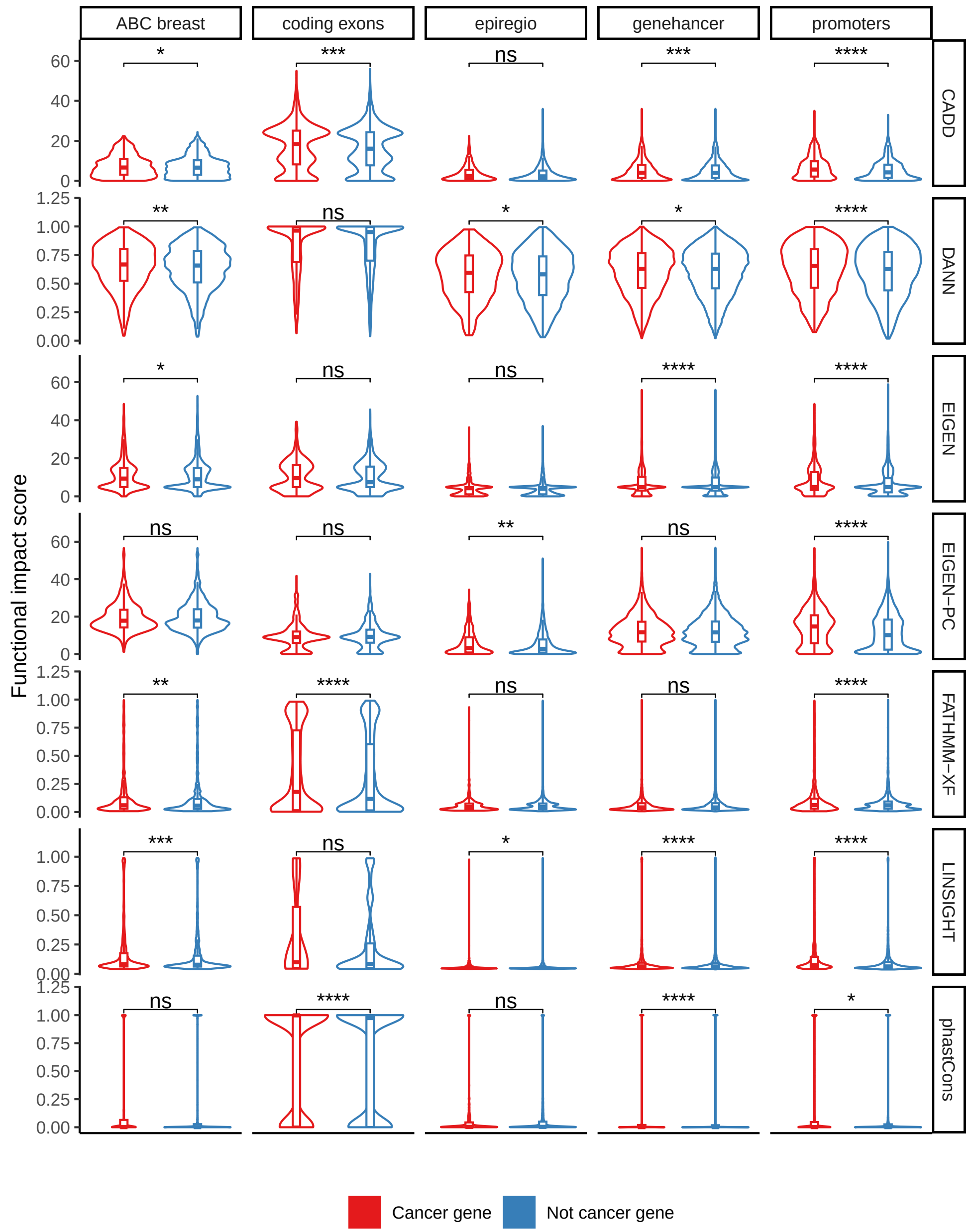

Fig N: Impact score distributions per region and score type for the observed mutations in the BRCA-US cohort. Distribution of functional impact scores for cancer (red) and non-cancer (blue) genes separated by association and scoring type. Significance labels indicate the p-value for the one-sided Wilcoxon test checking whether the $\log_{10}$(mutation rate) for the cancer genes $>$ $\log_{10}$(mutation rate) for the non-cancer genes. Significance labels indicate: ns - $p > 0.05$; $* : p \leq 0.05$; $** : p \leq 0.01$; $*** : p \leq 0.001$; $**** : p \leq 0.0001$.

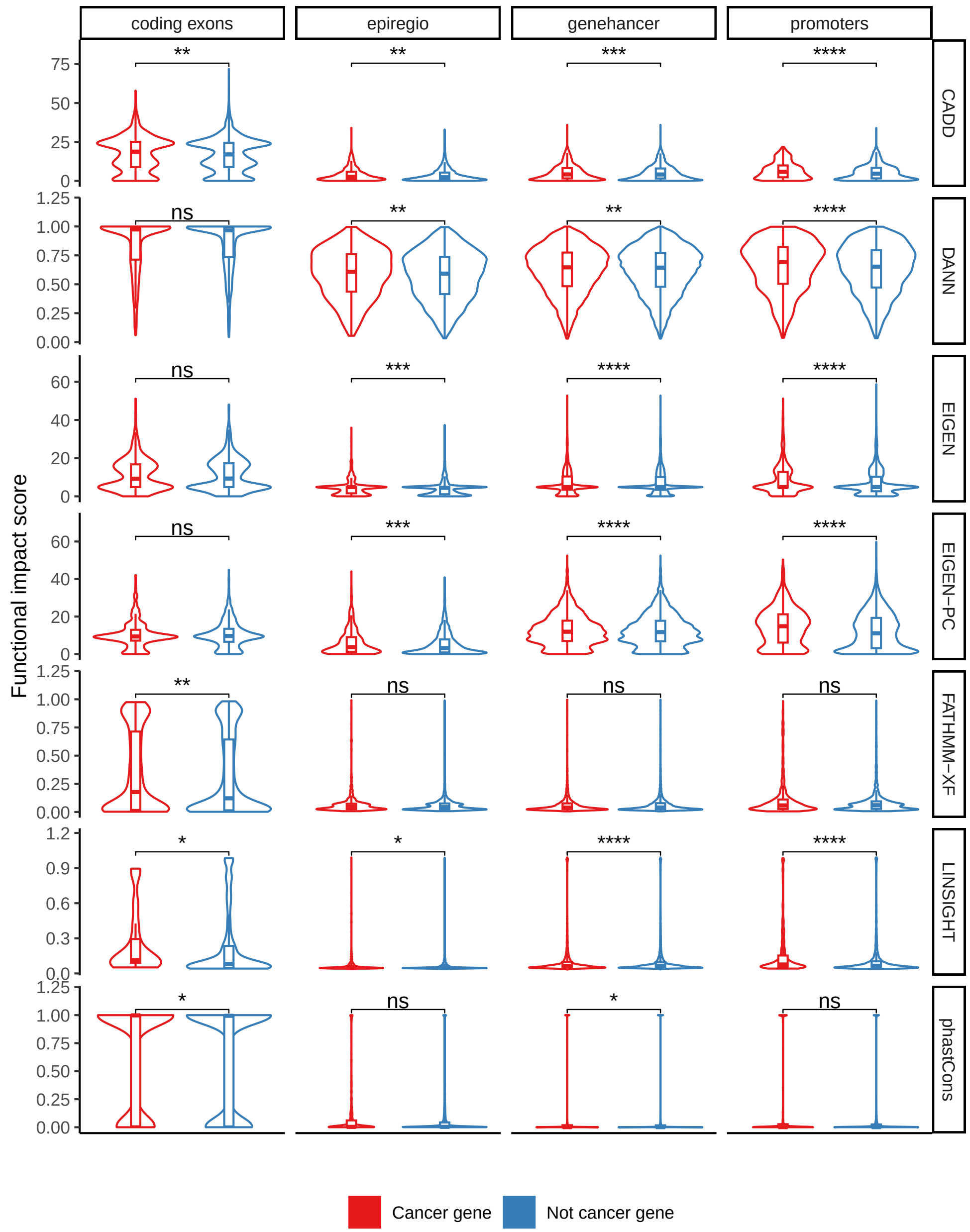

Fig O: Impact score distributions per region and score type for the observed mutations in the HNSC-US cohort. Distribution of functional impact scores for cancer (red) and non-cancer (blue) genes separated by association and scoring type. Significance labels indicate the p-value for the one-sided Wilcoxon test checking whether the $\log_{10}$(mutation rate) for the cancer genes $>$ $\log_{10}$(mutation rate) for the non-cancer genes. Significance labels indicate: ns - $p > 0.05$; $* : p \leq 0.05$; $** : p \leq 0.01$; $*** : p \leq 0.001$; $**** : p \leq 0.0001$.

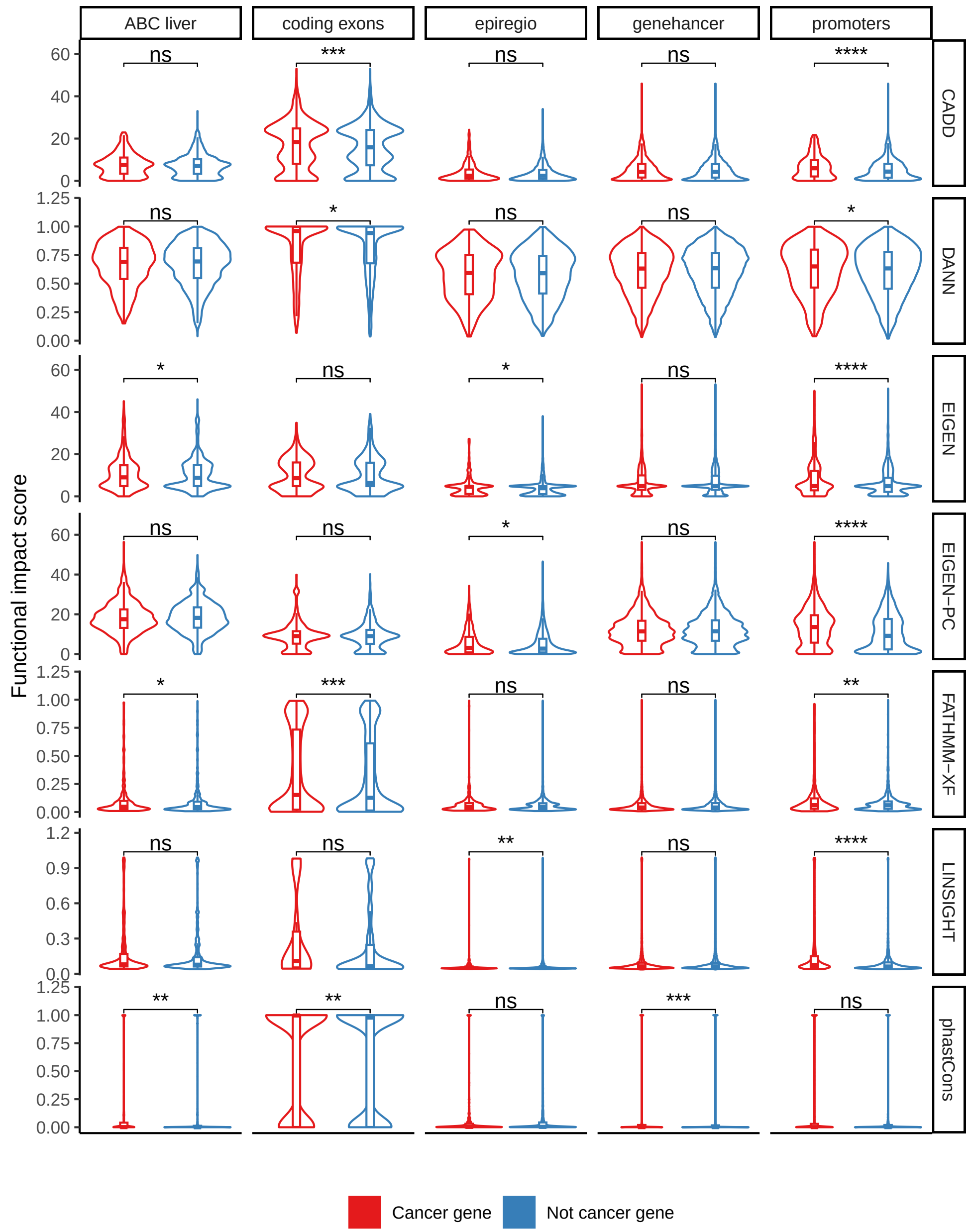

Fig P: Impact score distributions per region and score type for the observed mutations in the LIHC-US cohort. Distribution of functional impact scores for cancer (red) and non-cancer (blue) genes separated by association and scoring type. Significance labels indicate the p-value for the one-sided Wilcoxon test checking whether the $\log_{10}$(mutation rate) for the cancer genes $>$ $\log_{10}$(mutation rate) for the non-cancer genes. Significance labels indicate: ns - $p > 0.05$; $* : p \leq 0.05$; $** : p \leq 0.01$; $*** : p \leq 0.001$; $**** : p \leq 0.0001$.

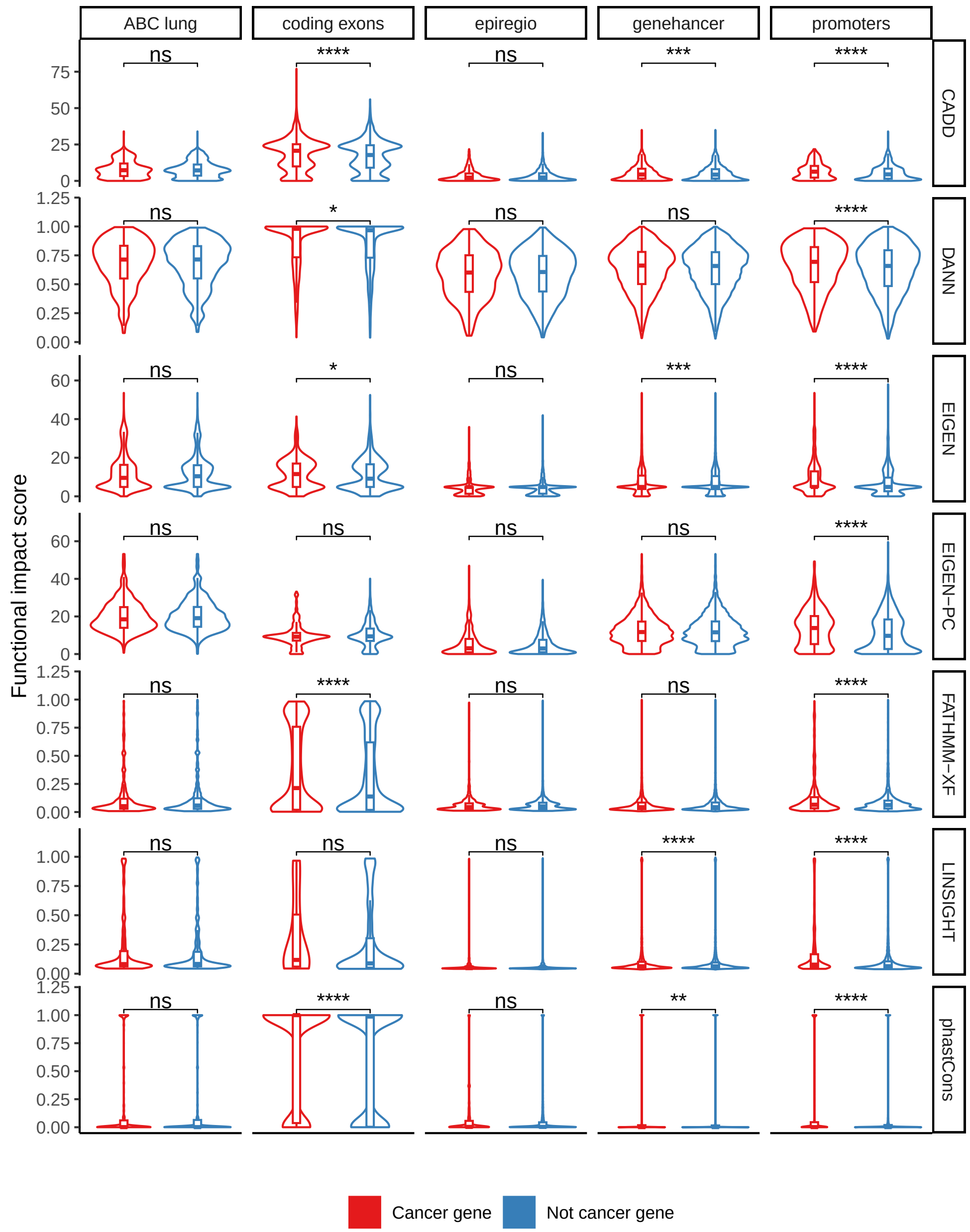

Fig Q: Impact score distributions per region and score type for the observed mutations in the LUAD-US cohort. Distribution of functional impact scores for cancer (red) and non-cancer (blue) genes separated by association and scoring type. Significance labels indicate the p-value for the one-sided Wilcoxon test checking whether the $\log_{10}$(mutation rate) for the cancer genes $>$ $\log_{10}$(mutation rate) for the non-cancer genes. Significance labels indicate: ns - $p > 0.05$; $*: p \leq 0.05$; $**: p \leq 0.01$; $***: p \leq 0.001$; $****: p \leq 0.0001$.

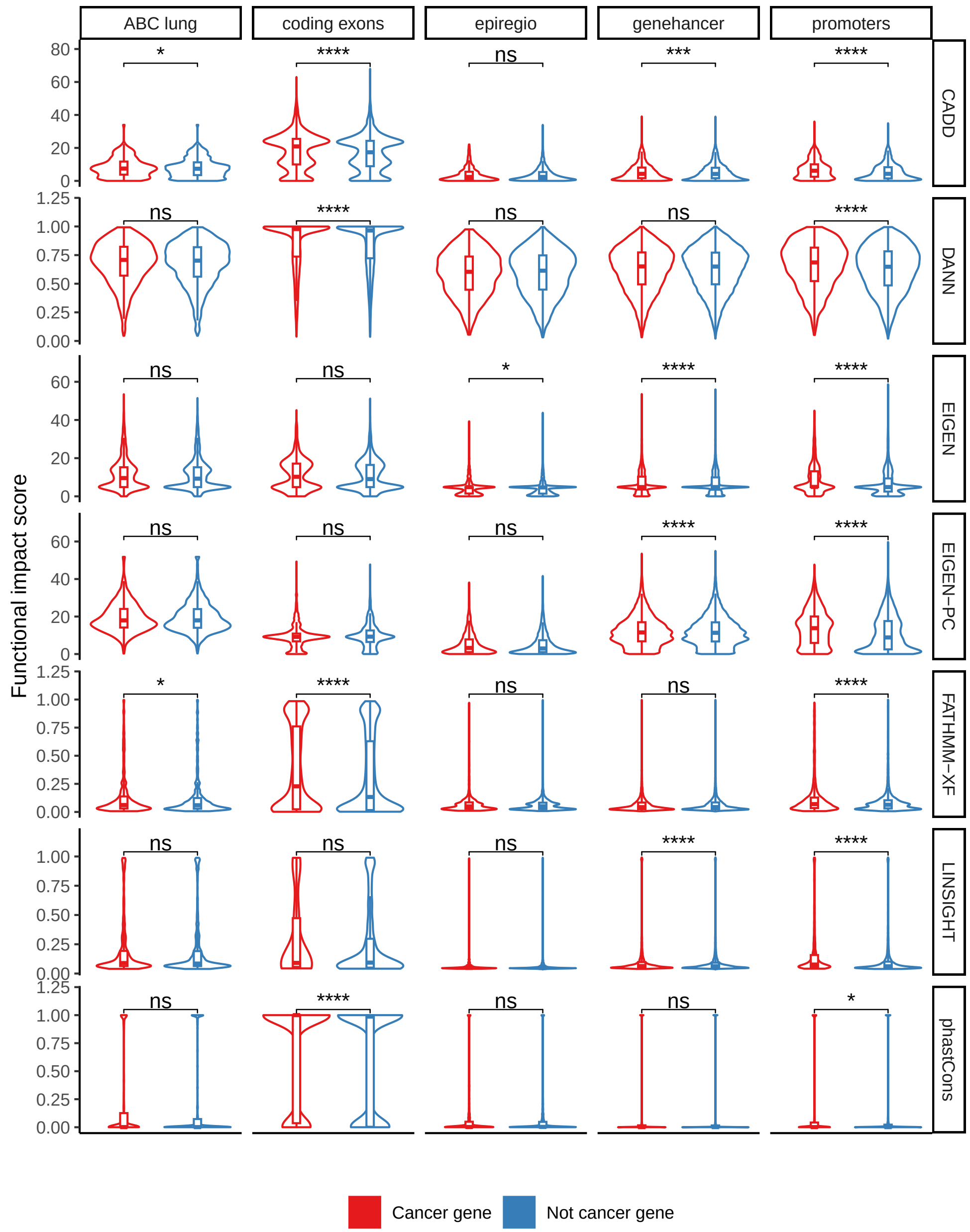

Fig R: Impact score distributions per region and score type for the observed mutations in the LUSC-US cohort. Distribution of functional impact scores for cancer (red) and non-cancer (blue) genes separated by association and scoring type. Significance labels indicate the p-value for the one-sided Wilcoxon test checking whether the $\log_{10}$(mutation rate) for the cancer genes $>$ $\log_{10}$(mutation rate) for the non-cancer genes. Significance labels indicate: ns - $p > 0.05$; $* : p \leq 0.05$; $** : p \leq 0.01$; $*** : p \leq 0.001$; $**** : p \leq 0.0001$.

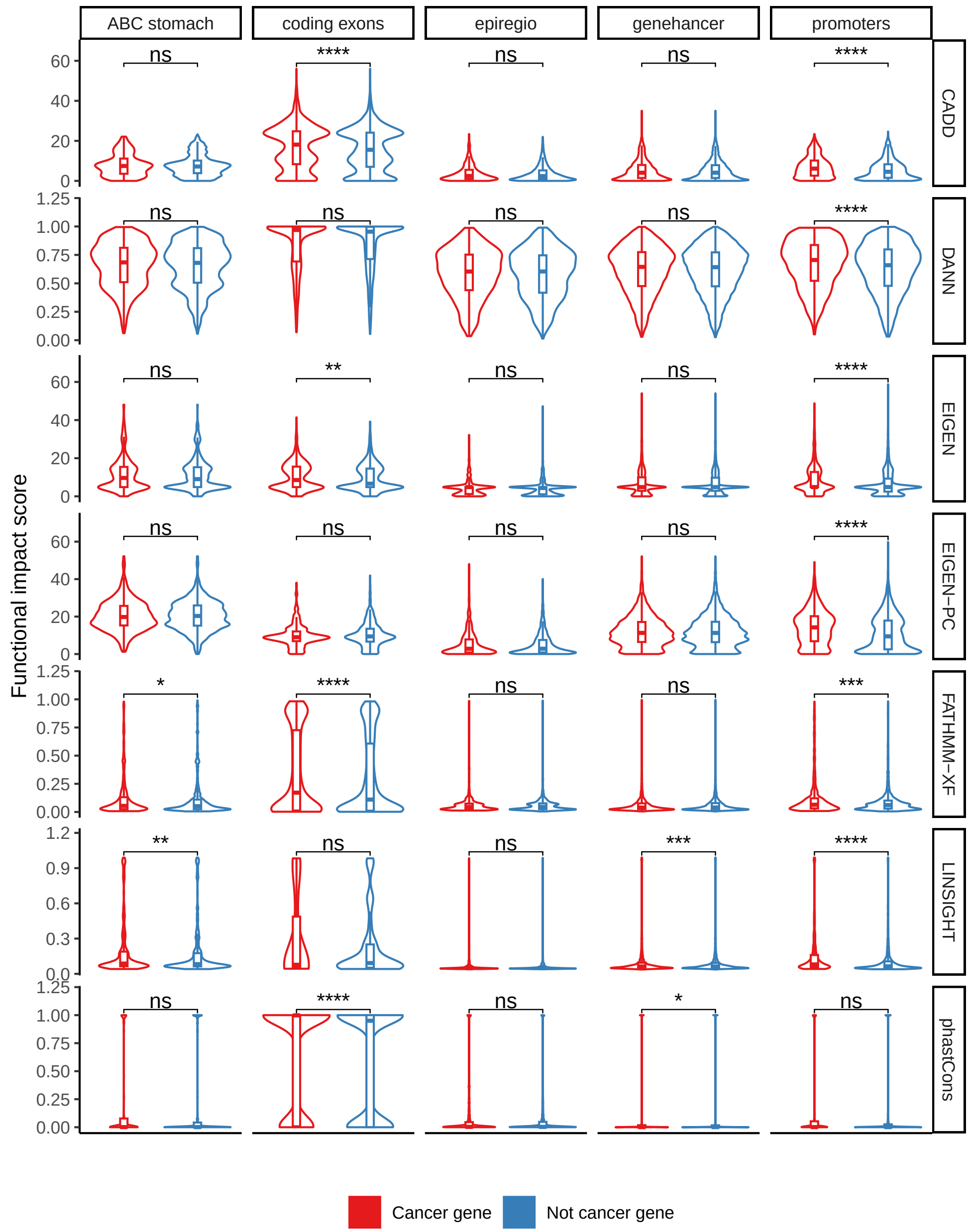

Fig S: Impact score distributions per region and score type for the observed mutations in the STAD-US cohort. Distribution of functional impact scores for cancer (red) and non-cancer (blue) genes separated by association and scoring type. Significance labels indicate the p-value for the one-sided Wilcoxon test checking whether the $\log_{10}$(mutation rate) for the cancer genes $>$ $\log_{10}$(mutation rate) for the non-cancer genes. Significance labels indicate: ns - $p > 0.05$; $* : p \leq 0.05$; $** : p \leq 0.01$; $*** : p \leq 0.001$; $**** : p \leq 0.0001$.

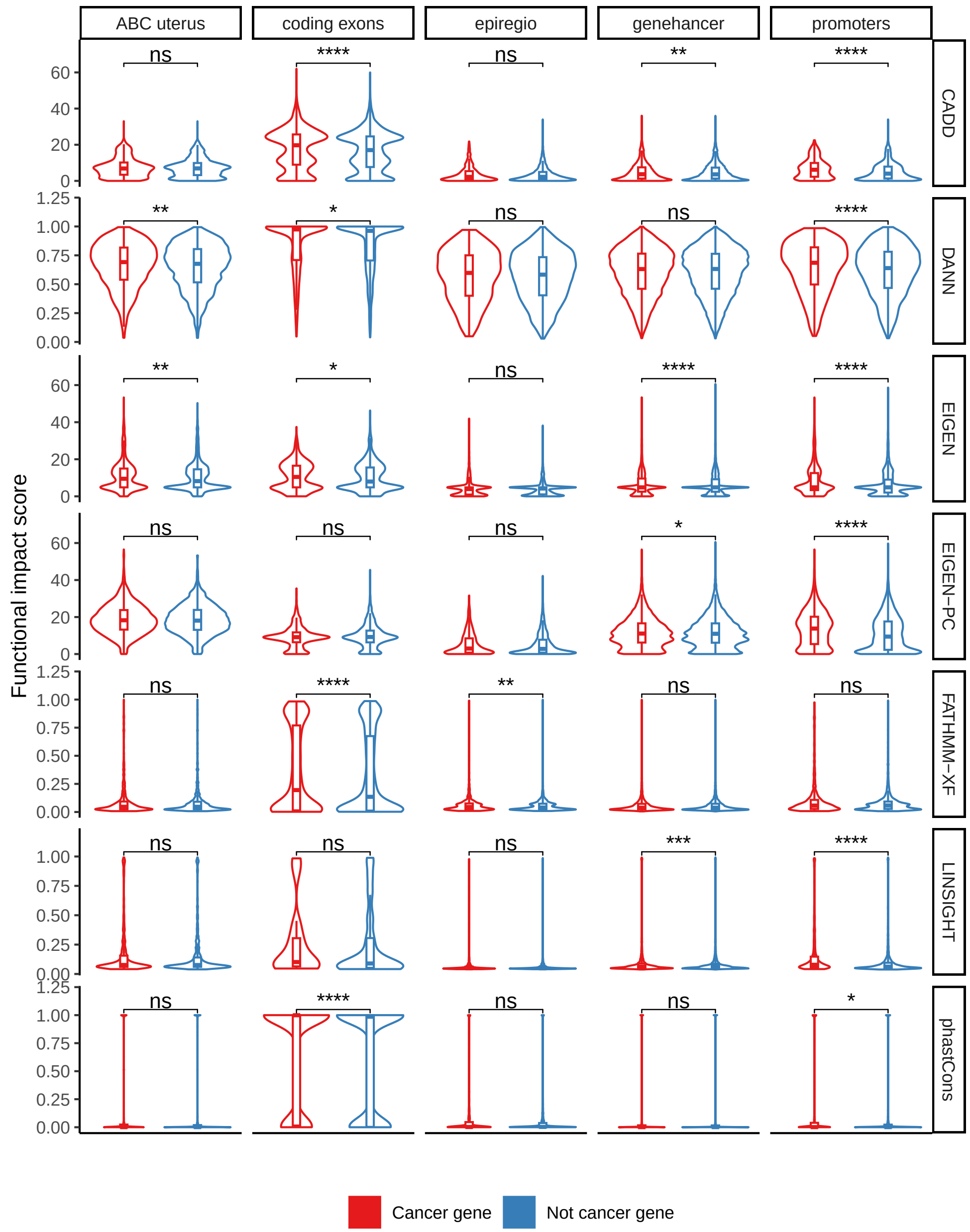

Fig T: Impact score distributions per region and score type for the observed mutations in the UCEC-US cohort. Distribution of functional impact scores for cancer (red) and non-cancer (blue) genes separated by association and scoring type. Significance labels indicate the p-value for the one-sided Wilcoxon test checking whether the $\log_{10}$(mutation rate) for the cancer genes $>$ $\log_{10}$(mutation rate) for the non-cancer genes. Significance labels indicate: ns - $p > 0.05$; $* : p \leq 0.05$; $** : p \leq 0.01$; $*** : p \leq 0.001$; $**** : p \leq 0.0001$.

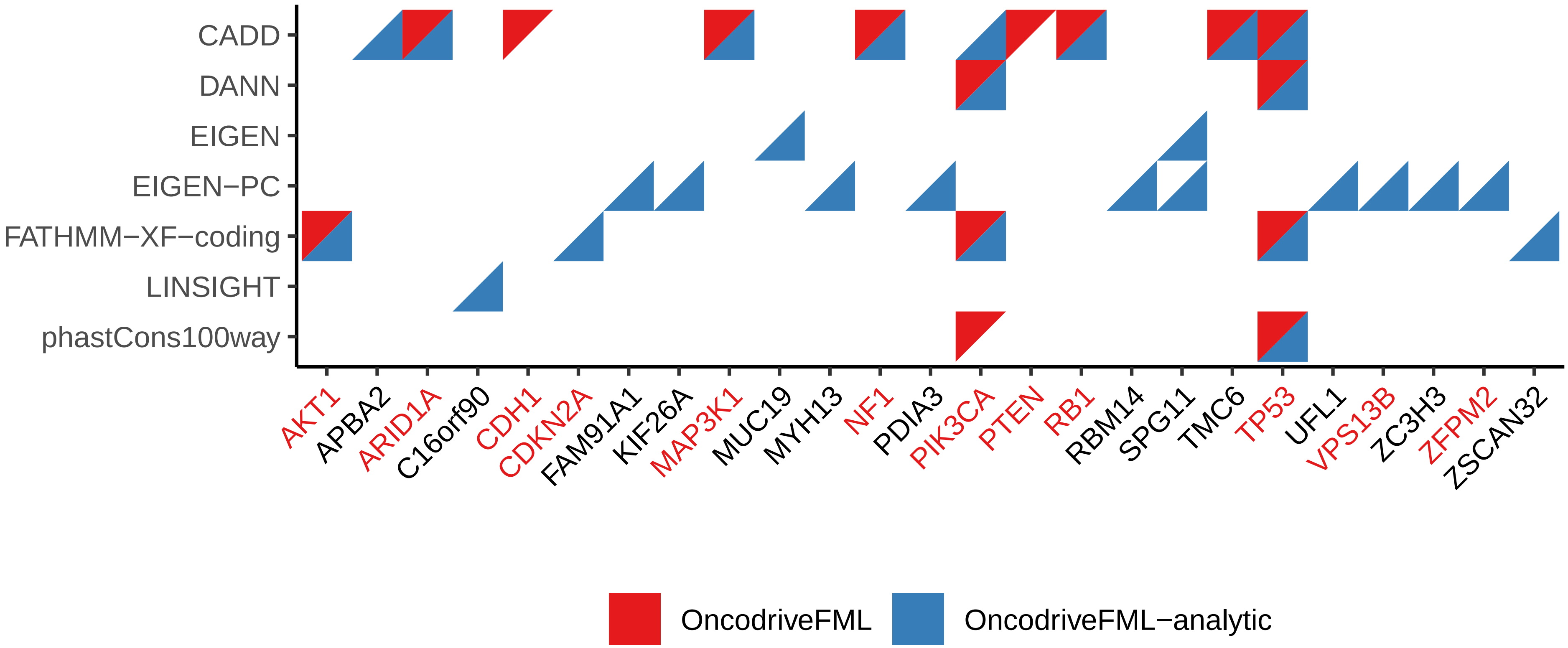

Fig U: Detected genes across scoring approaches in the analysis of coding exonic sequences with mutations from the ICGC breast cancer cohort. Genes (x-axis) showing an enrichment (adjusted-pval ≤ 0.05) of high functional impact mutations across different mutation scoring approaches (y-axis) detected by either OncodriveFML (red triangles) or OncodriveFML-analytic (blue triangles). Gene names in red are listed as cancer genes.

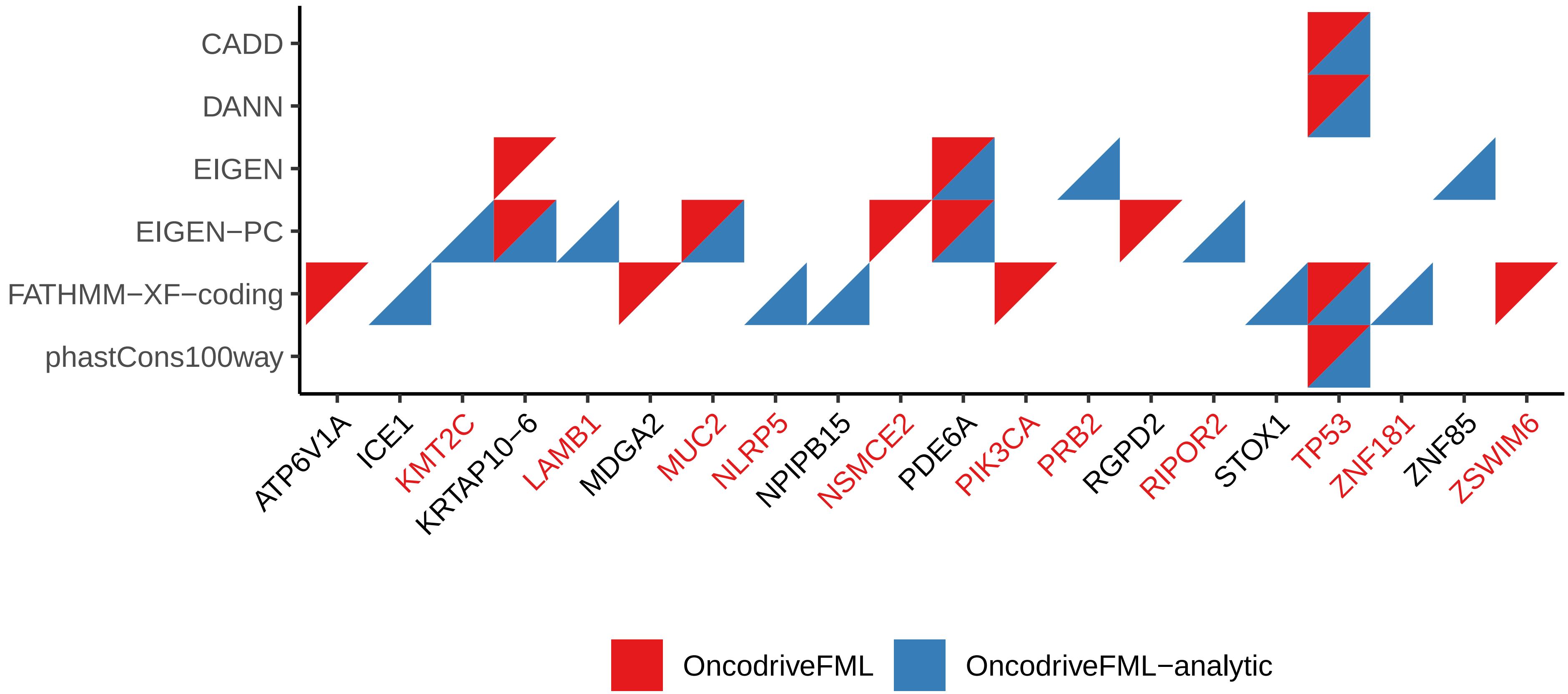

Fig V: Detected genes across scoring approaches in the analysis of coding exonic sequences with mutations from the BRCA-US cohort. Genes (x-axis) showing an enrichment (adjusted-pval $\leq$ 0.05) of high functional impact mutations across different mutation scoring approaches (y-axis) detected by either OncodriveFML (red triangles) or OncodriveFML-analytic (blue triangles). Gene names in red are listed as cancer genes.

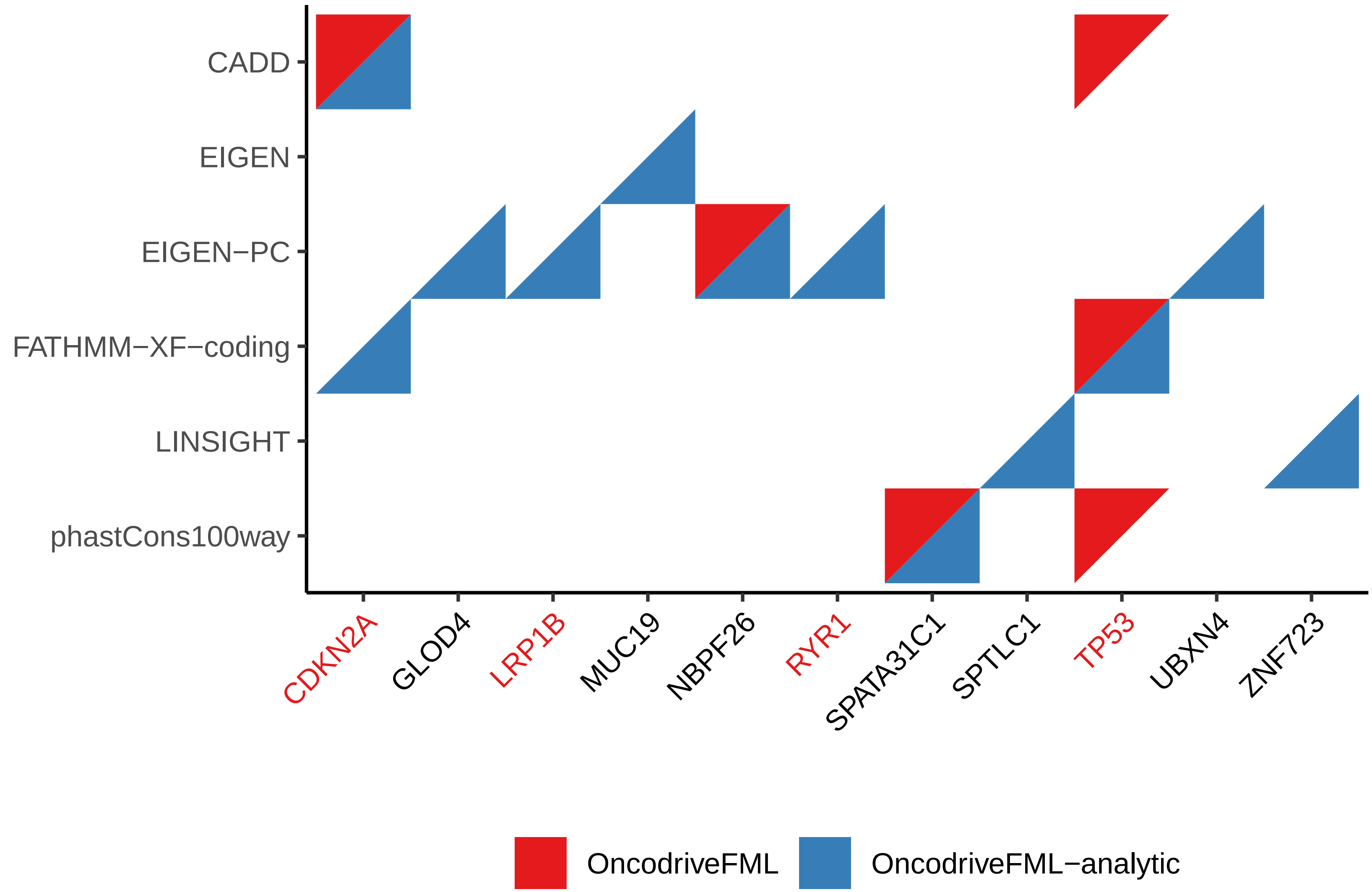

Fig W: Detected genes across scoring approaches in the analysis of coding exonic sequences with mutations from the HNSC-US cohort. Genes (x-axis) showing an enrichment (adjusted-pval $\leq$ 0.05) of high functional impact mutations across different mutation scoring approaches (y-axis) detected by either OncodriveFML (red triangles) or OncodriveFML-analytic (blue triangles). Gene names in red are listed as cancer genes.

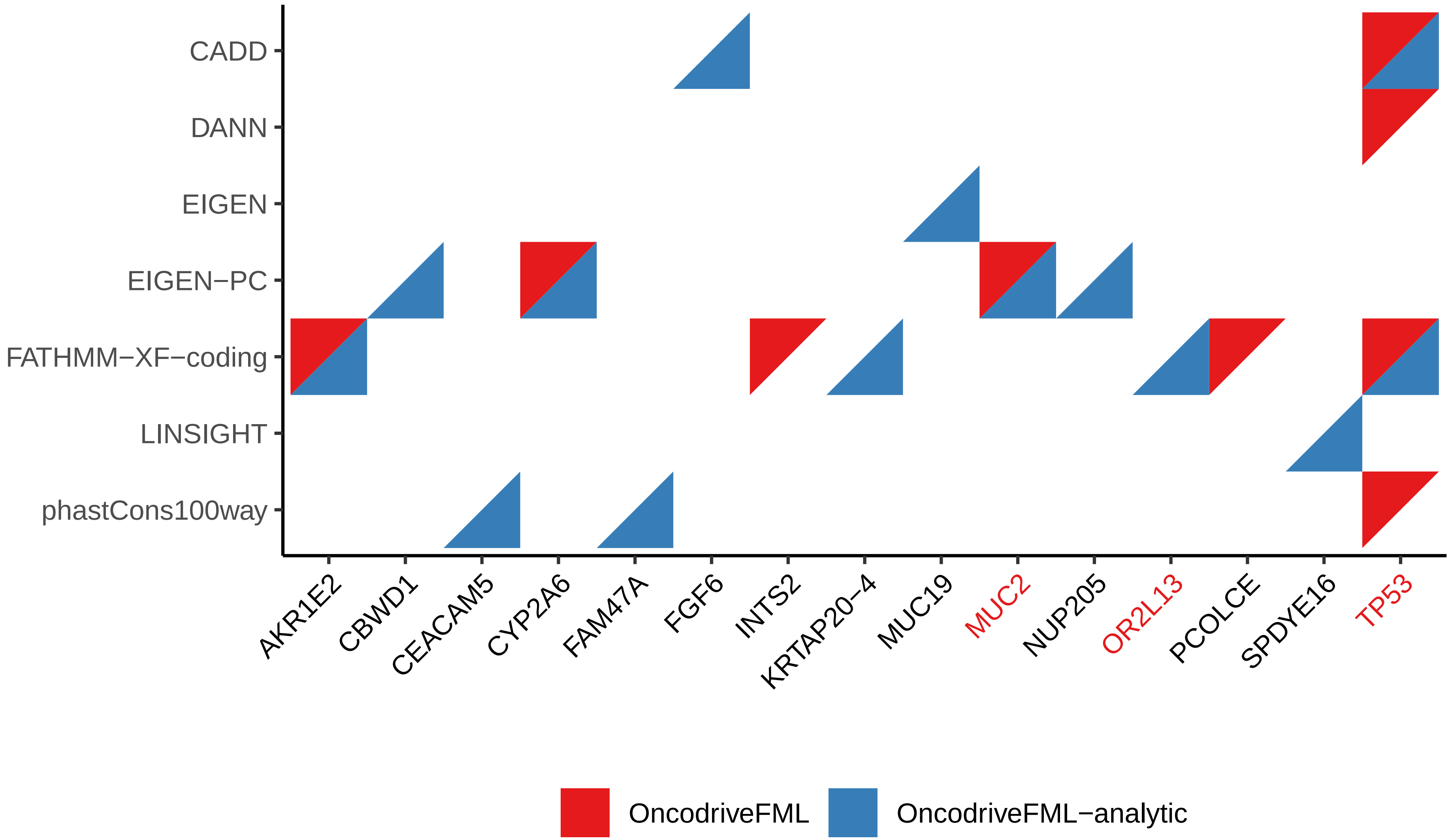

Fig X: Detected genes across scoring approaches in the analysis of coding exonic sequences with mutations from the LIHC-US cohort. Genes (x-axis) showing an enrichment (adjusted-pval $\leq$ 0.05) of high functional impact mutations across different mutation scoring approaches (y-axis) detected by either OncodriveFML (red triangles) or OncodriveFML-analytic (blue triangles). Gene names in red are listed as cancer genes.

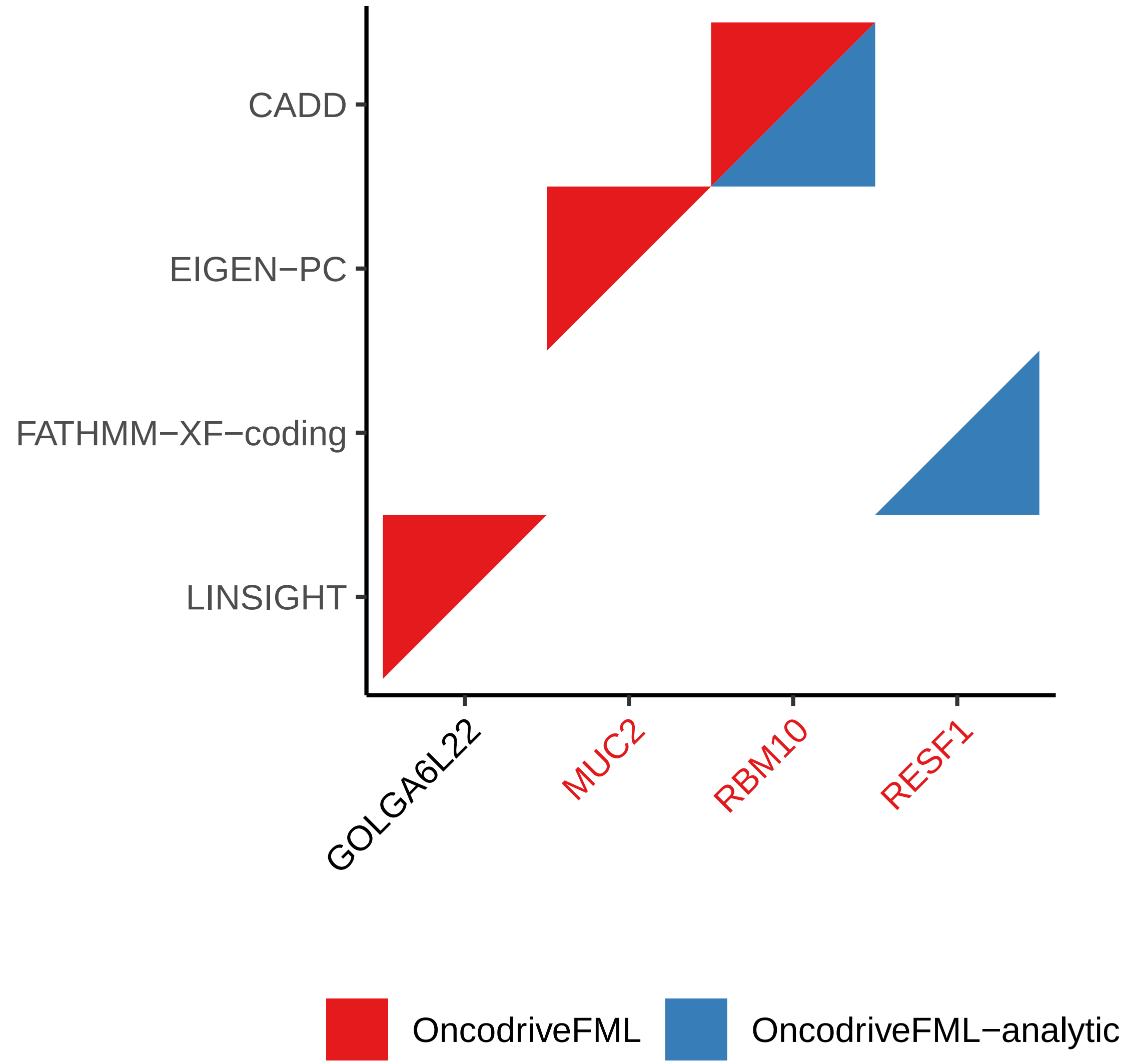

Fig Y: Detected genes across scoring approaches in the analysis of coding exonic sequences with mutations from the LUAD-US cohort. Genes (x-axis) showing an enrichment (adjusted-pval $\leq$ 0.05) of high functional impact mutations across different mutation scoring approaches (y-axis) detected by either OncodriveFML (red triangles) or OncodriveFML-analytic (blue triangles). Gene names in red are listed as cancer genes.

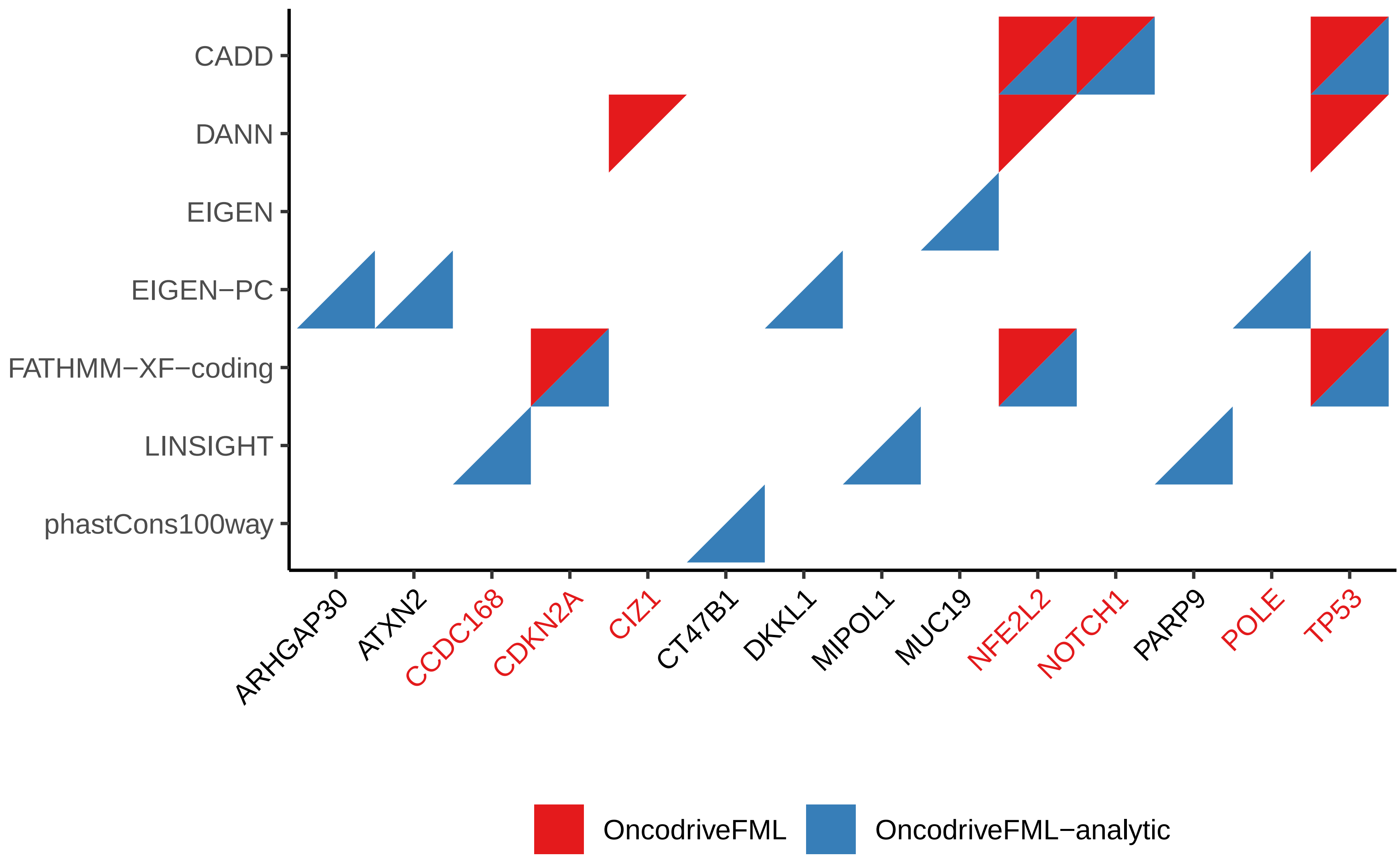

Fig Z: Detected genes across scoring approaches in the analysis of coding exonic sequences with mutations from the LUSC-US cohort. Genes (x-axis) showing an enrichment (adjusted-pval ≤ 0.05) of high functional impact mutations across different mutation scoring approaches (y-axis) detected by either OncodriveFML (red triangles) or OncodriveFML-analytic (blue triangles). Gene names in red are listed as cancer genes.

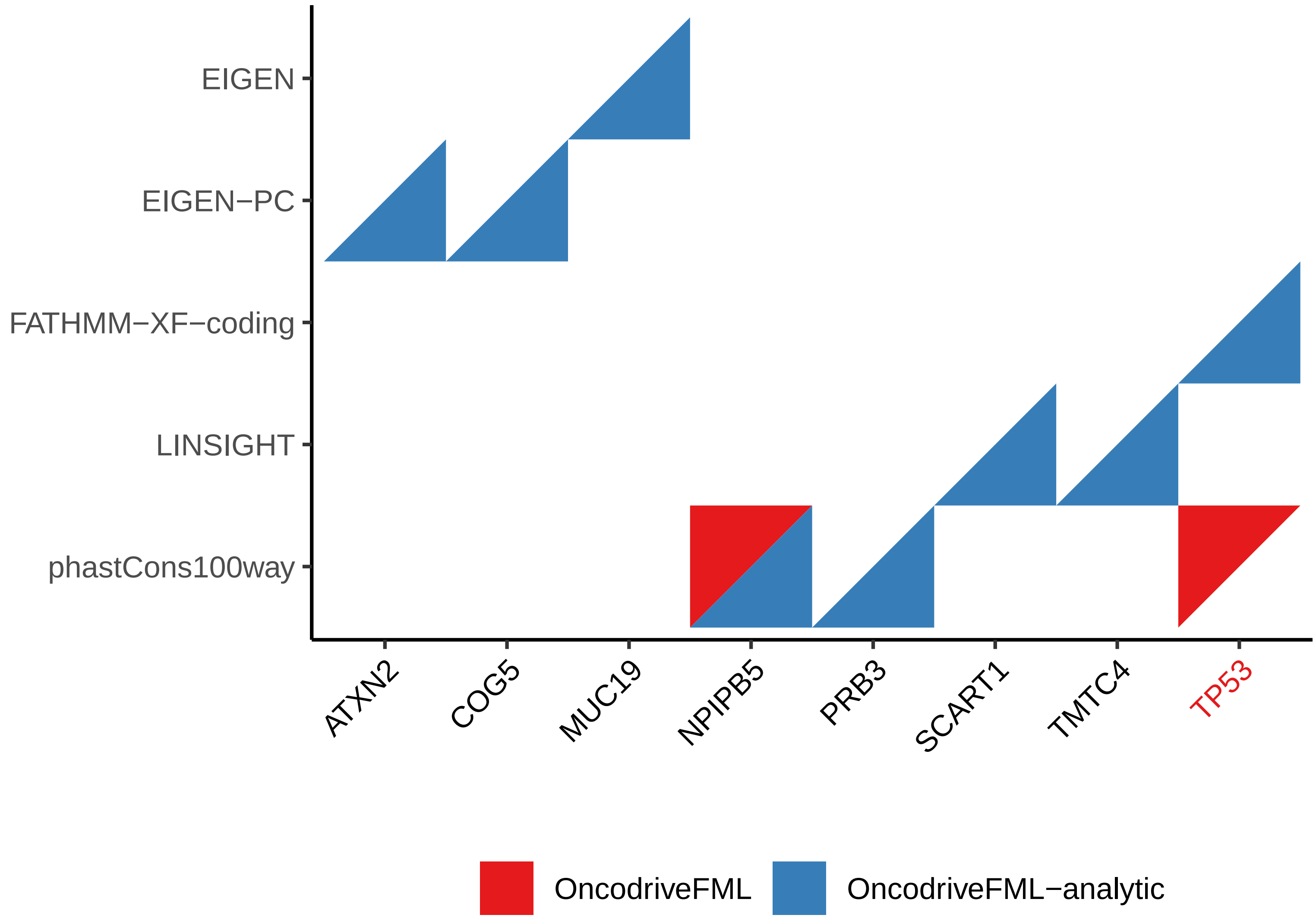

Fig AA: Detected genes across scoring approaches in the analysis of coding exonic sequences with mutations from the STAD-US cohort. Genes (x-axis) showing an enrichment (adjusted-pval $\leq$ 0.05) of high functional impact mutations across different mutation scoring approaches (y-axis) detected by either OncodriveFML (red triangles) or OncodriveFML-analytic (blue triangles). Gene names in red are listed as cancer genes.

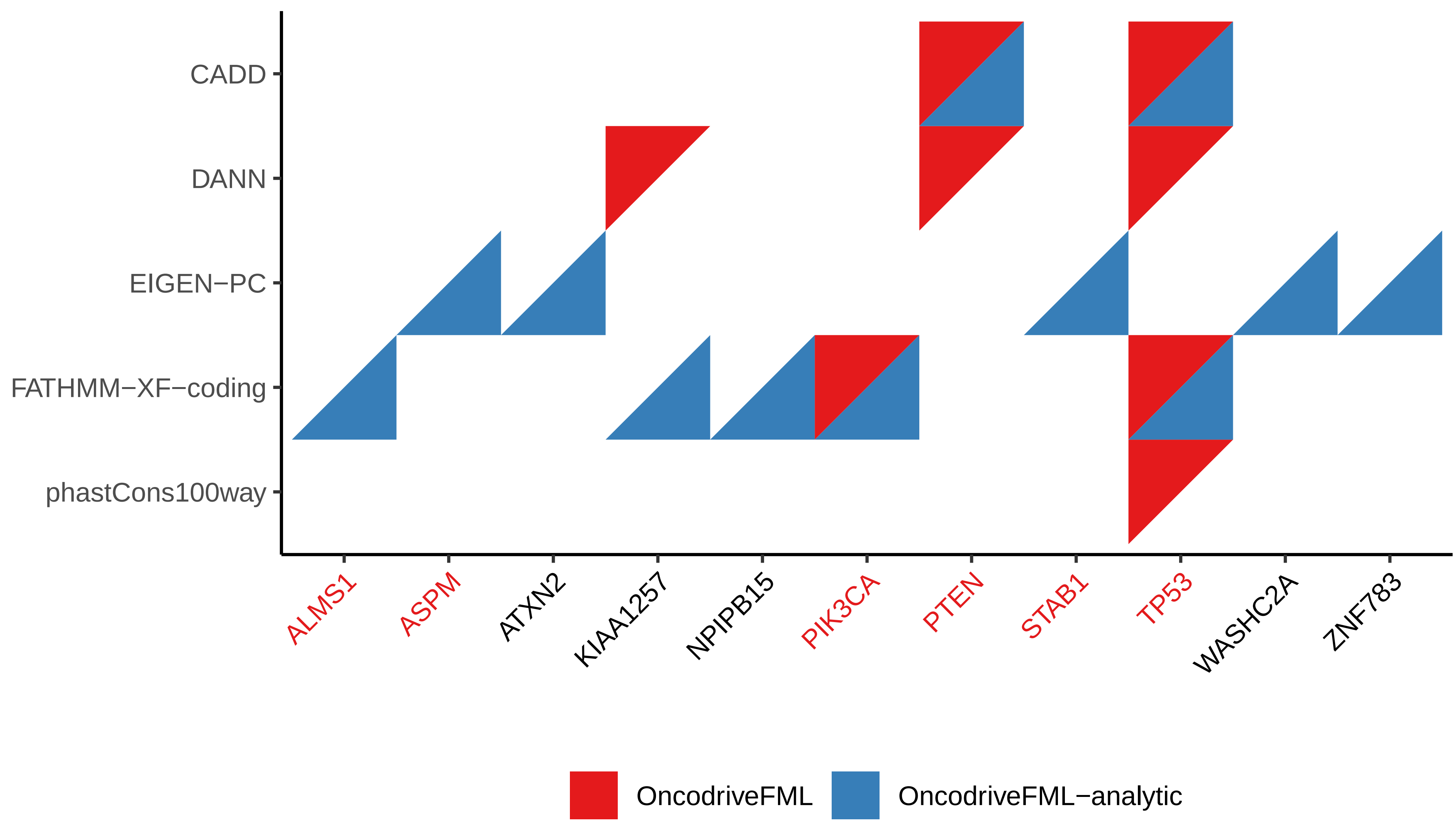

Fig AB: Detected genes across scoring approaches in the analysis of coding exonic sequences with mutations from the UCEC-US cohort. Genes (x-axis) showing an enrichment (adjusted-pval $\leq$ 0.05) of high functional impact mutations across different mutation scoring approaches (y-axis) detected by either OncodriveFML (red triangles) or OncodriveFML-analytic (blue triangles). Gene names in red are listed as cancer genes.

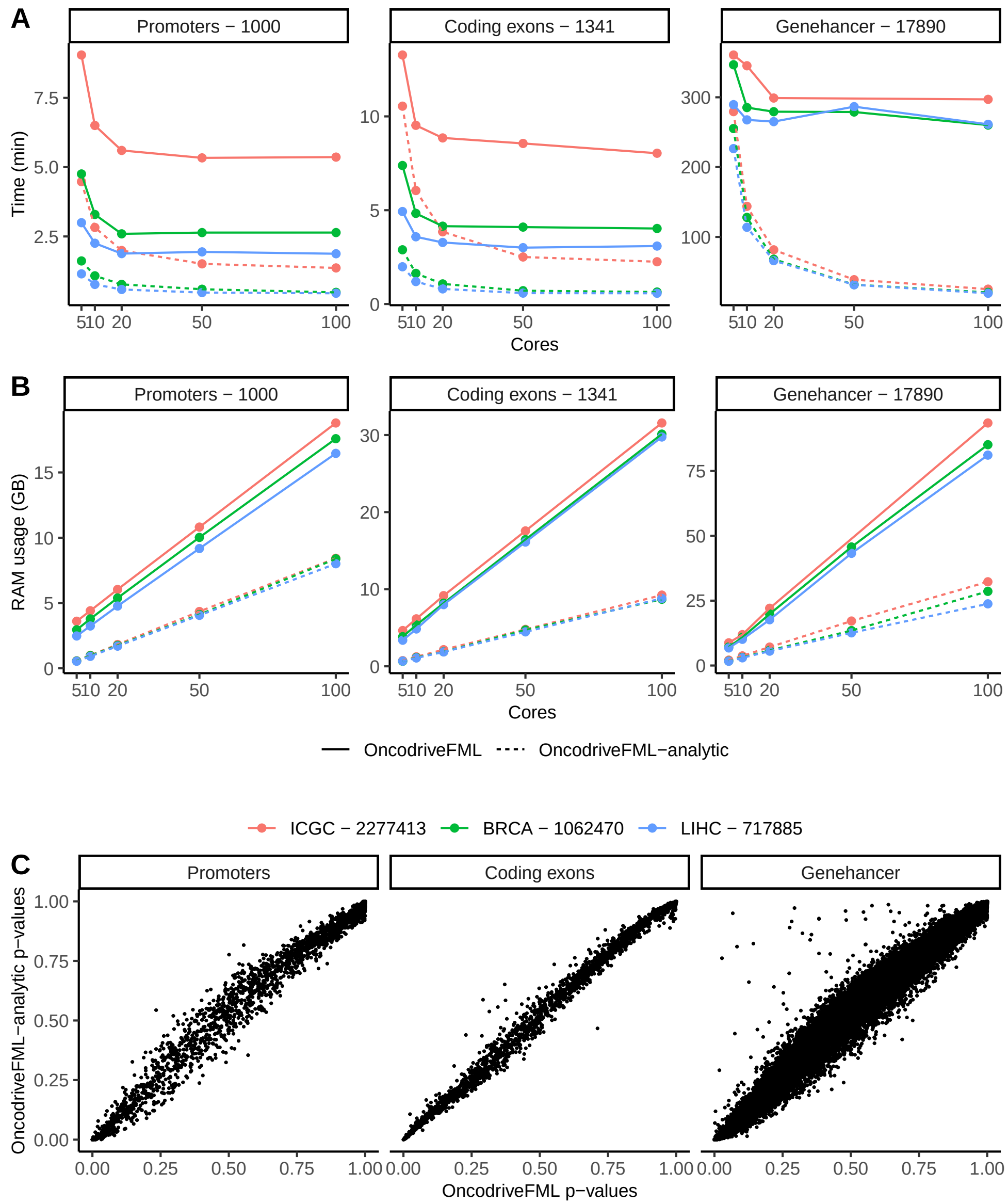

Fig AC: Computational benchmark of OncodriveFML-analytic and OncodriveFML. Elapsed time in minutes **(A)** and RAM usage in GB **(B)** (y-axis) versus numbers of cores (x-axis) used by OncodriveFML-analytic (dashed line) or OncodriveFML (solid line) in regions of increasing sizes. Region median sizes are indicated in the facet titles. Colors indicate the used cohorts in the benchmark together with the total number of observed mutations in each cohort. **(C)** Comparison of each gene's p-values between OncodriveFML (x-axis) and OncodriveFML-analytic (y-axis).

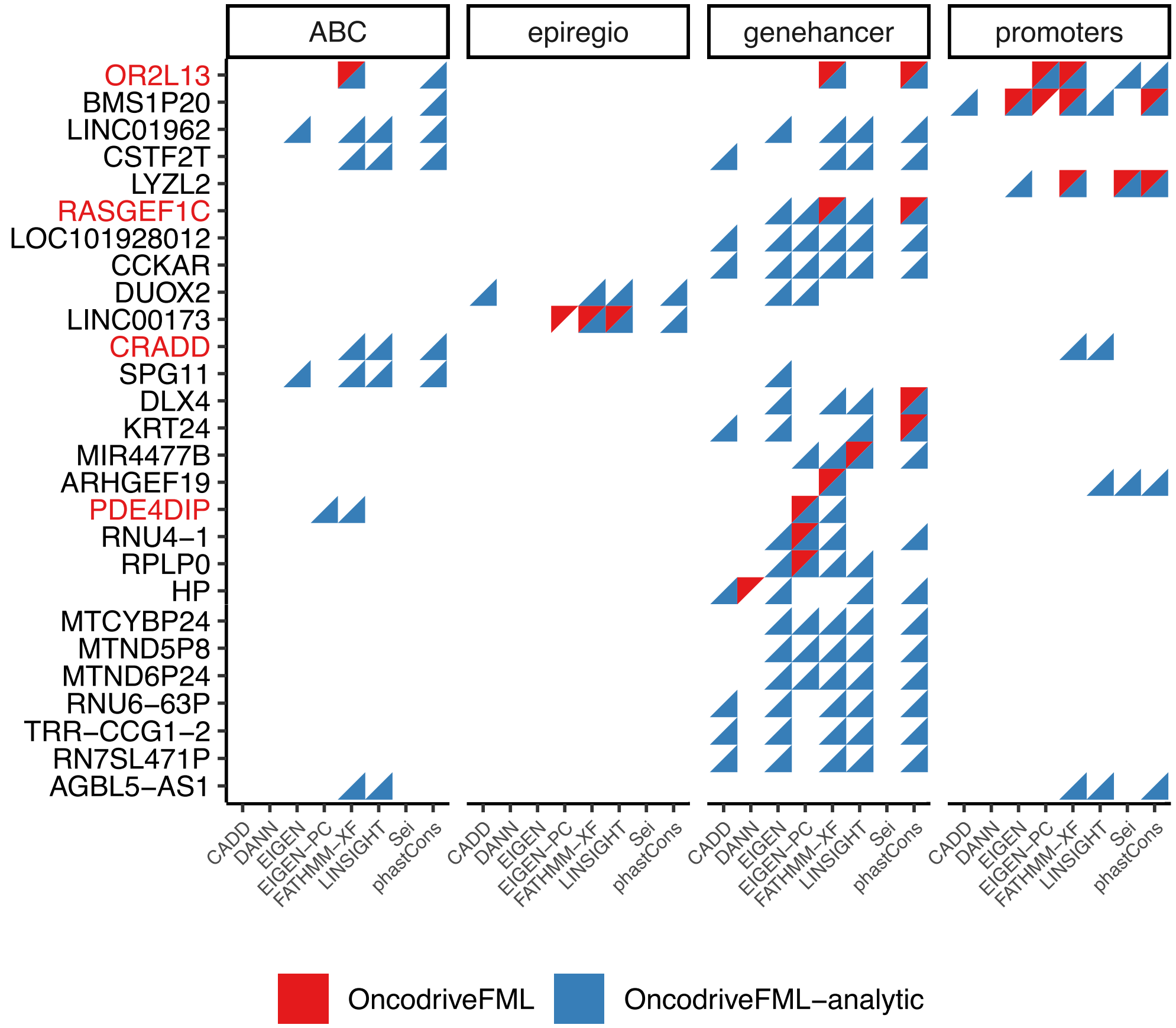

Fig AD: Detected genes across CRR-score combinations in the TCGA BRCA cancer cohort. Genes (y-axis) showing an enrichment (adjusted-pval $\leq$ 0.05) of FI CRVs in different score (x-axis) and association (facet) combinations detected by either OncodriveFML (red triangles) or OncodriveFML-analytic (blue triangles). Only genes detected in at least five or more CRR-score combinations and/or enrichment method are shown. Gene names in red are listed as cancer genes.

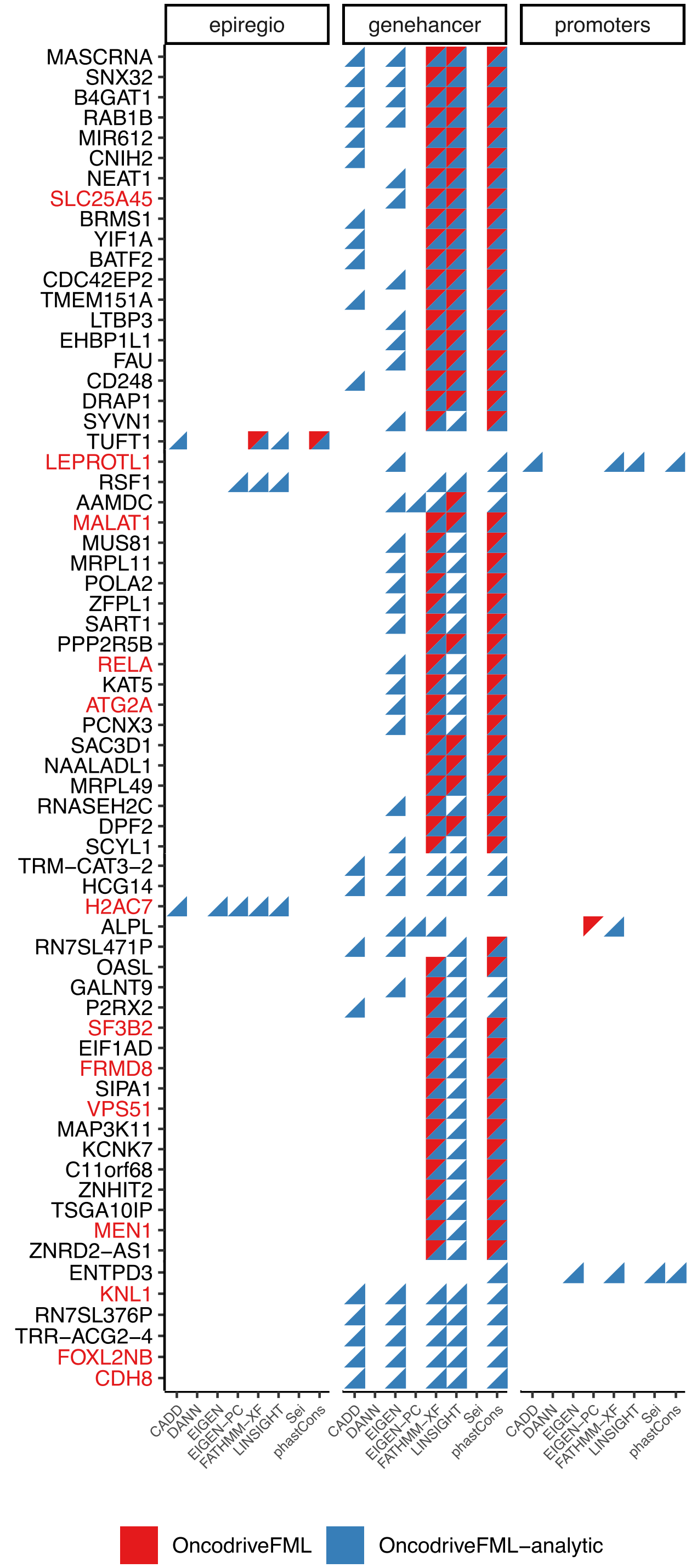

Fig AE: Detected genes across CRR-score combinations in the TCGA HNSC cancer cohort. Genes (y-axis) showing an enrichment (adjusted-pval $\leq$ 0.05) of FI CRVs in different score (x-axis) and association (facet) combinations detected by either OncodriveFML (red triangles) or OncodriveFML-analytic (blue triangles). Only genes detected in at least five or more CRR-score combinations and/or enrichment method are shown. Gene names in red are listed as cancer genes.

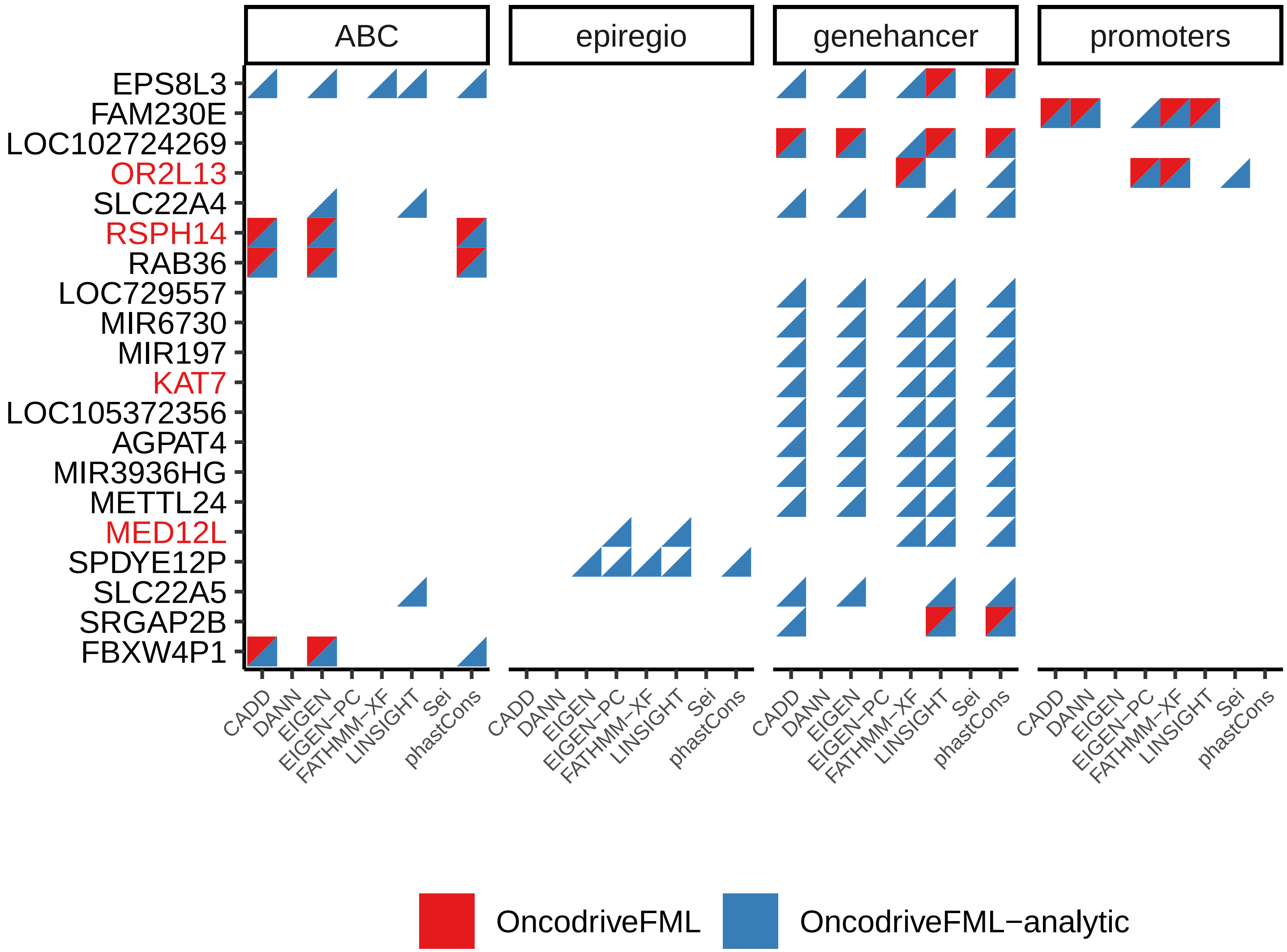

Fig AF: Detected genes across CRR-score combinations in the TCGA LIHC cancer cohort. Genes (y-axis) showing an enrichment (adjusted-pval $\leq$ 0.05) of FI CRVs in different score (x-axis) and association (facet) combinations detected by either OncodriveFML (red triangles) or OncodriveFML-analytic (blue triangles). Only genes detected in at least five or more CRR-score combinations and/or enrichment method are shown. Gene names in red are listed as cancer genes.

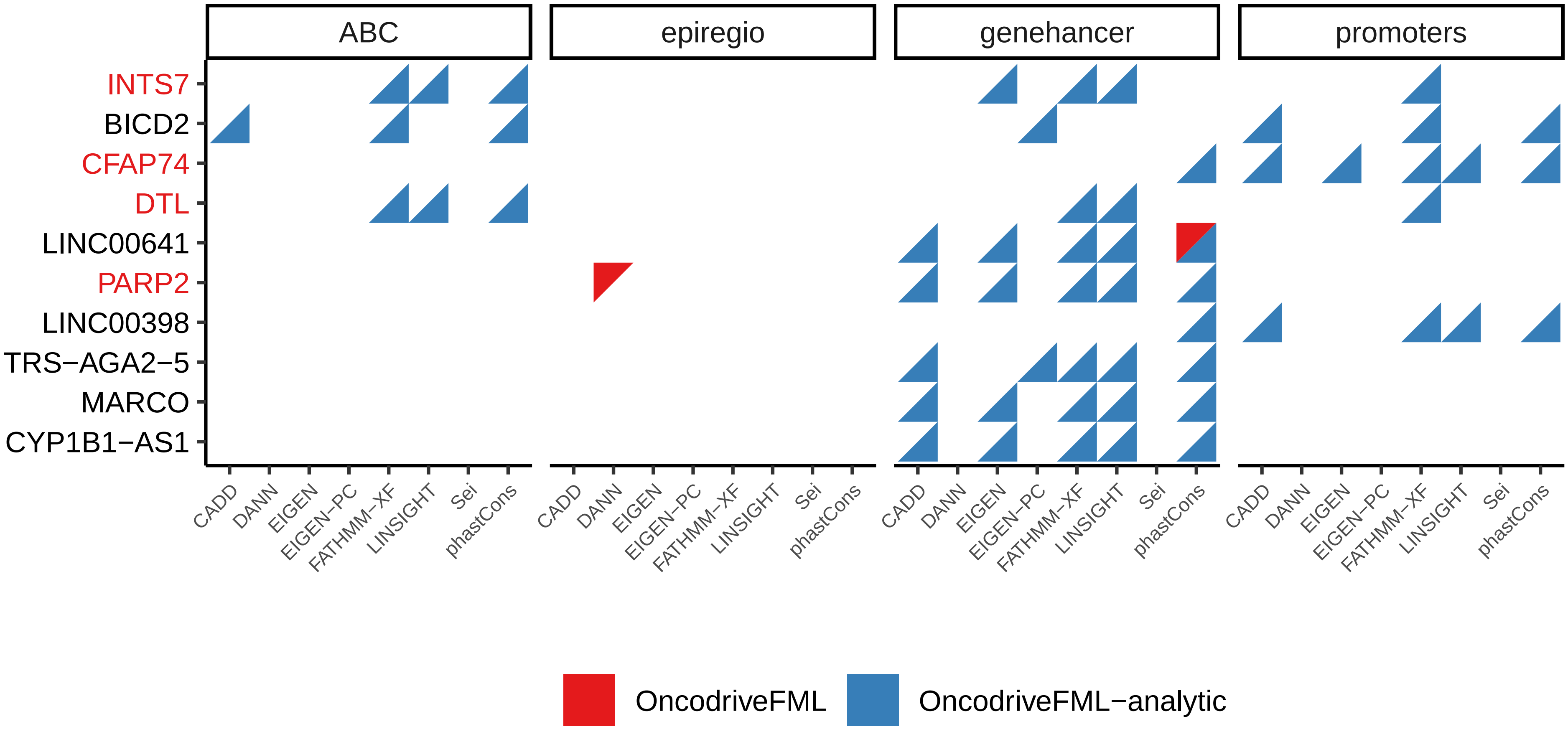

Fig AG: Detected genes across CRR-score combinations in the TCGA LUAD cancer cohort. Genes (y-axis) showing an enrichment (adjusted-pval $\leq$ 0.05) of FI CRVs in different score (x-axis) and association (facet) combinations detected by either OncodriveFML (red triangles) or OncodriveFML-analytic (blue triangles). Only genes detected in at least five or more CRR-score combinations and/or enrichment method are shown. Gene names in red are listed as cancer genes.

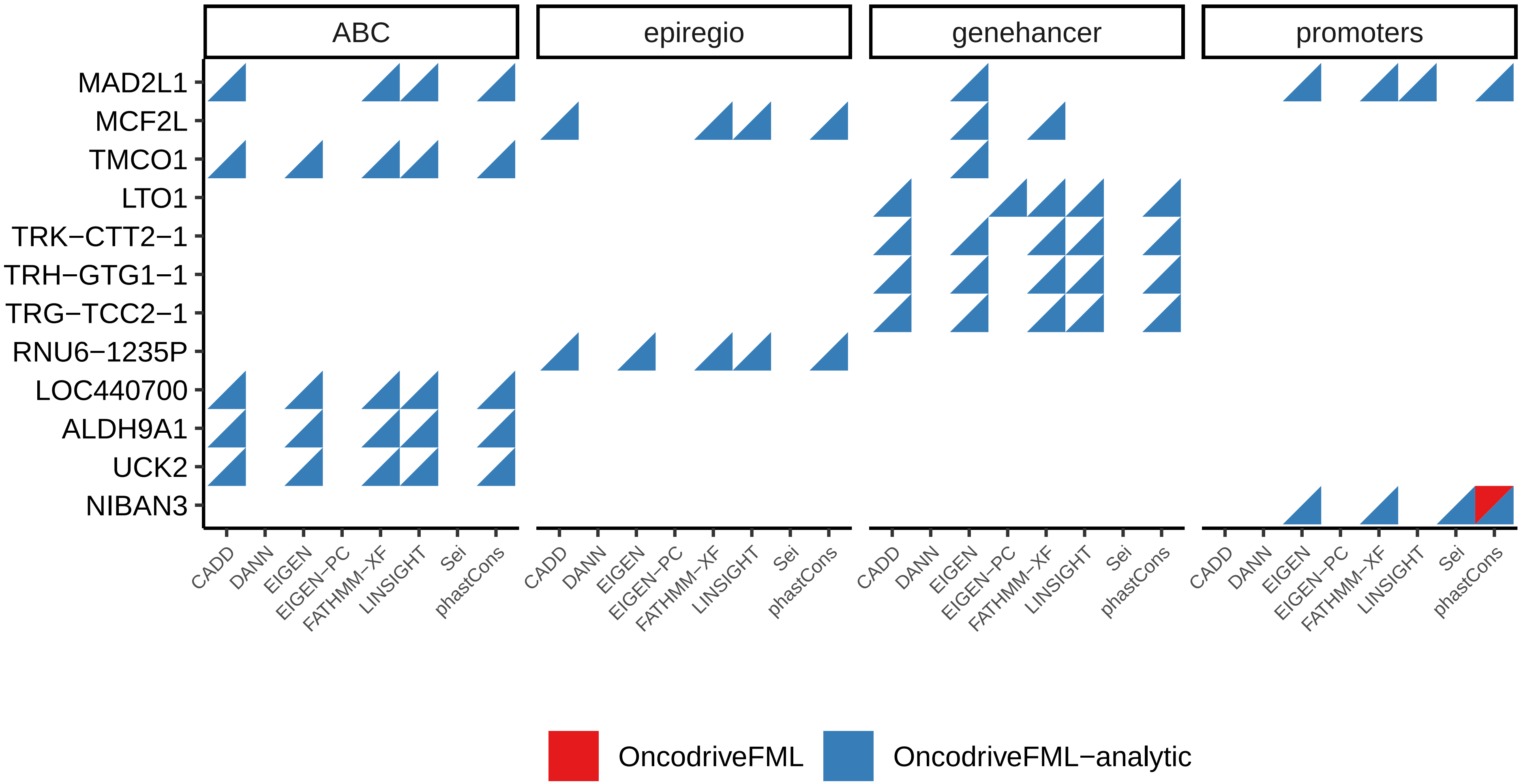

Fig AH: Detected genes across CRR-score combinations in the TCGA LUSC cancer cohort. Genes (y-axis) showing an enrichment (adjusted-pval $\leq$ 0.05) of FI CRVs in different score (x-axis) and association (facet) combinations detected by either OncodriveFML (red triangles) or OncodriveFML-analytic (blue triangles). Only genes detected in at least five or more CRR-score combinations and/or enrichment method are shown. Gene names in red are listed as cancer genes.

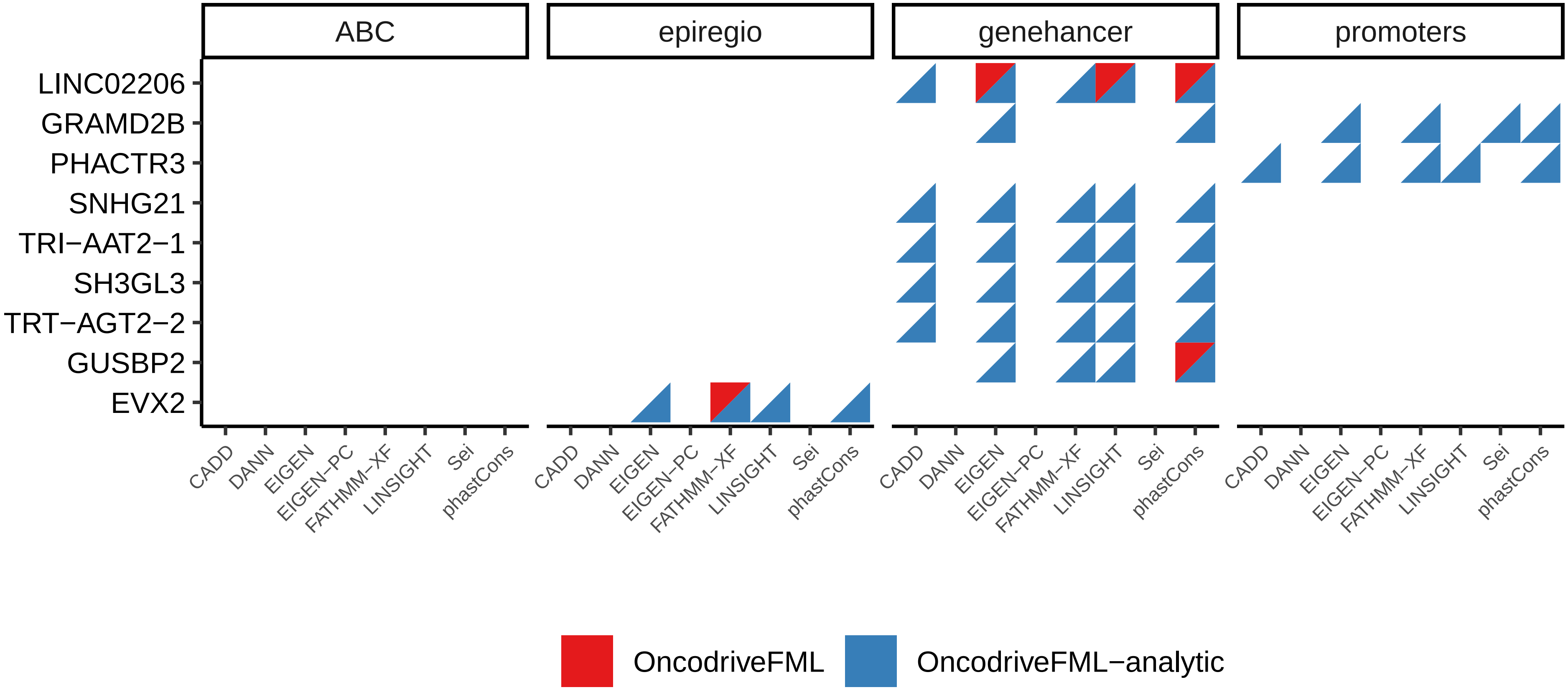

Fig AI: Detected genes across CRR-score combinations in the TCGA STAD cancer cohort. Genes (y-axis) showing an enrichment (adjusted-pval $\leq$ 0.05) of FI CRVs in different score (x-axis) and association (facet) combinations detected by either OncodriveFML (red triangles) or OncodriveFML-analytic (blue triangles). Only genes detected in at least five or more CRR-score combinations and/or enrichment method are shown. Gene names in red are listed as cancer genes.

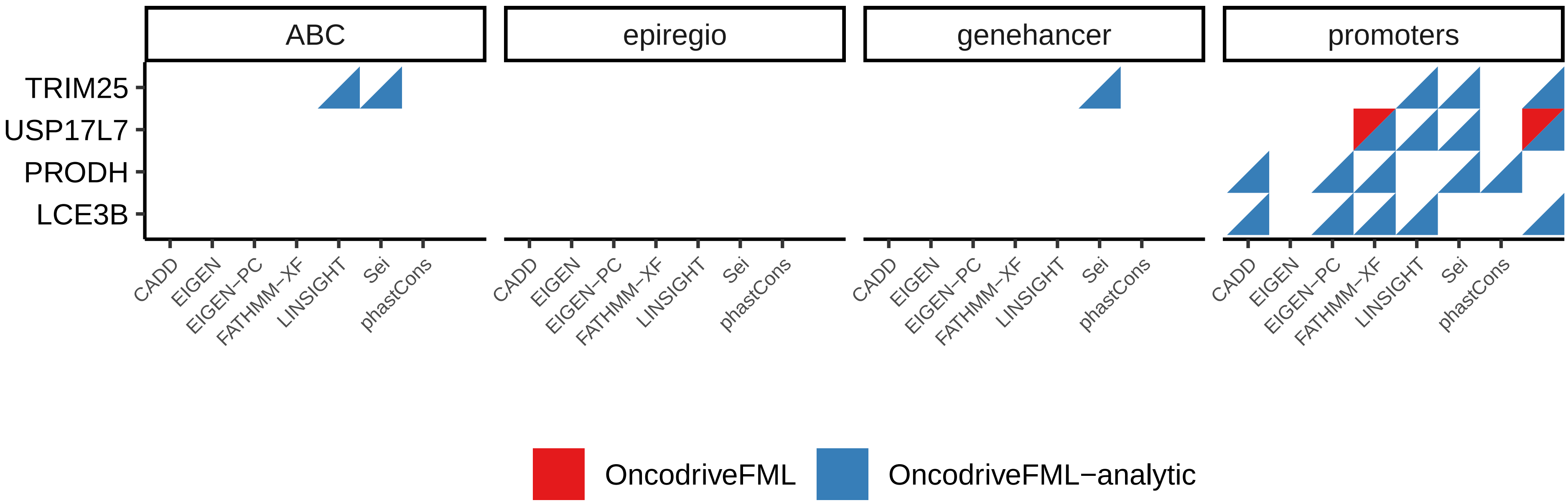

Fig AJ: Detected genes across CRR-score combinations in the TCGA UCEC cancer cohort. Genes (y-axis) showing an enrichment (adjusted-pval $\leq$ 0.05) of FI CRVs in different score (x-axis) and association (facet) combinations detected by either OncodriveFML (red triangles) or OncodriveFML-analytic (blue triangles). Only genes detected in at least five or more CRR-score combinations and/or enrichment method are shown. Gene names in red are listed as cancer genes.

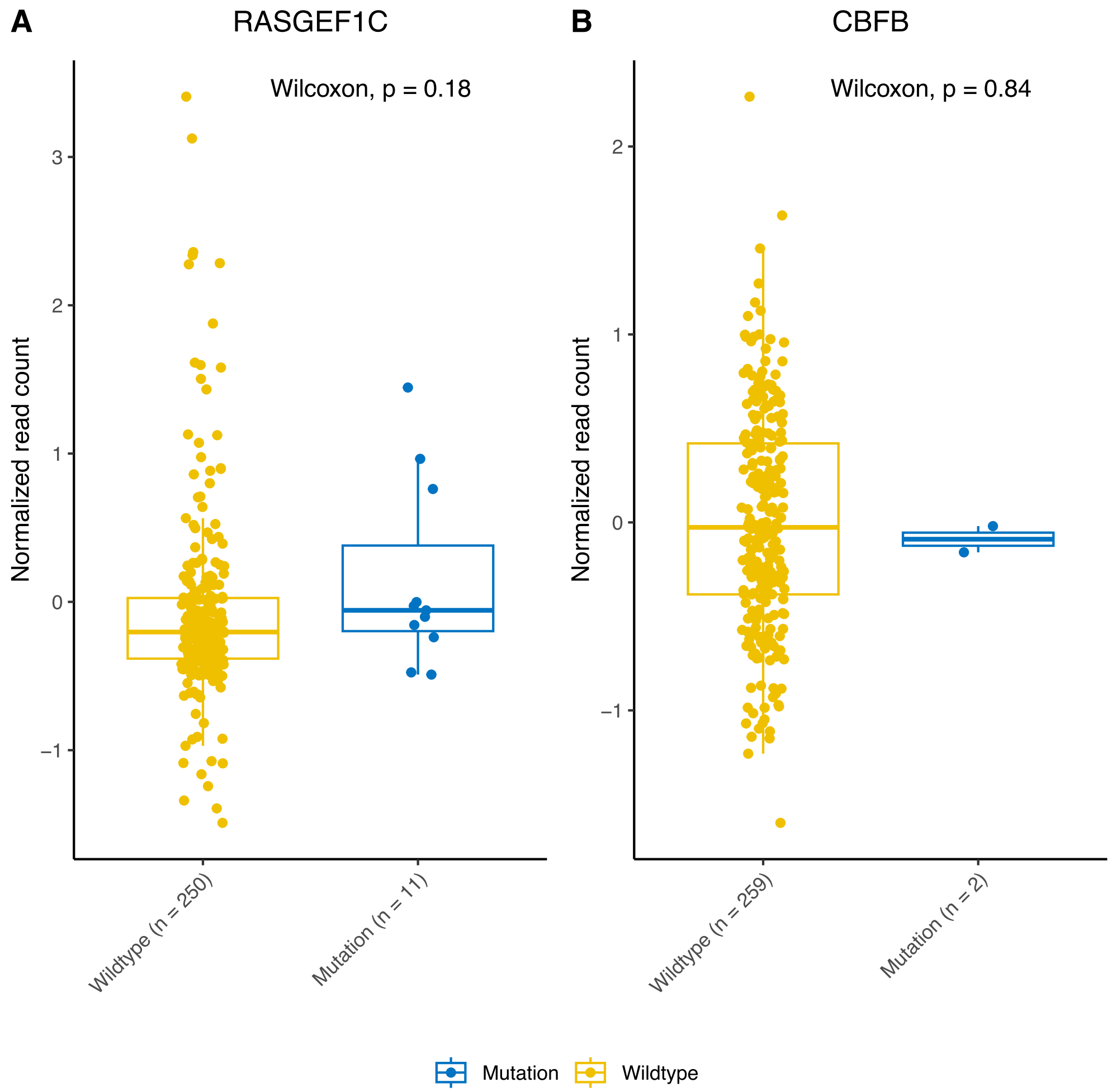

Fig AK: CBFB and RASGEF1C do not show changes in their expression. Boxplots comparing the normalized expression of RASGEF1C (left) and CBFB (right) stratified by patients showing mutations in their CRRs.

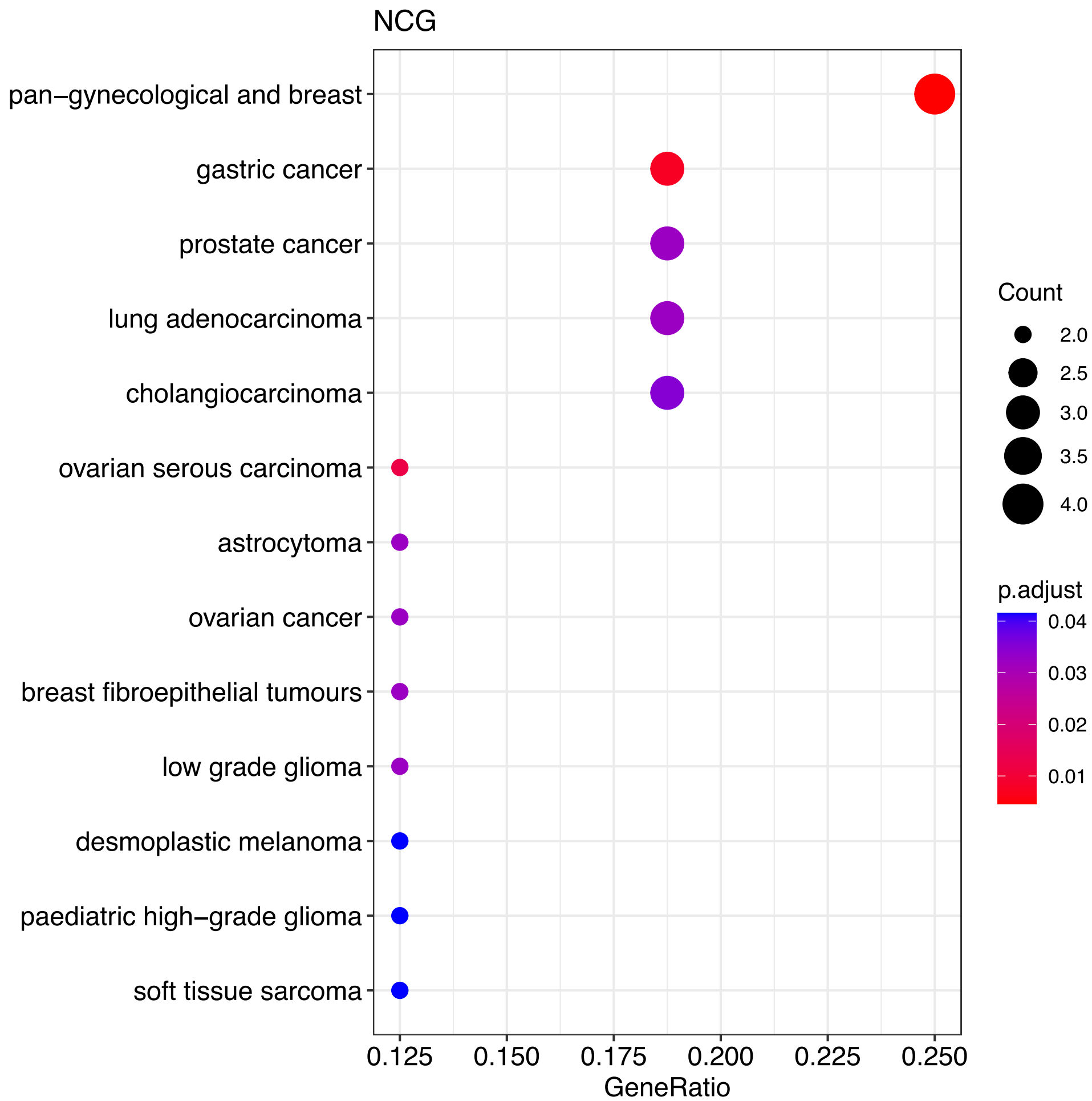

Fig AL: Enrichment of breast-cancer-related genes showing an enrichment of FI SNPs in their CRRs. Gene set enrichment analysis using the Network of Cancer Genes ontology on the genes with an enrichment of FI SNPs in their CRRs.

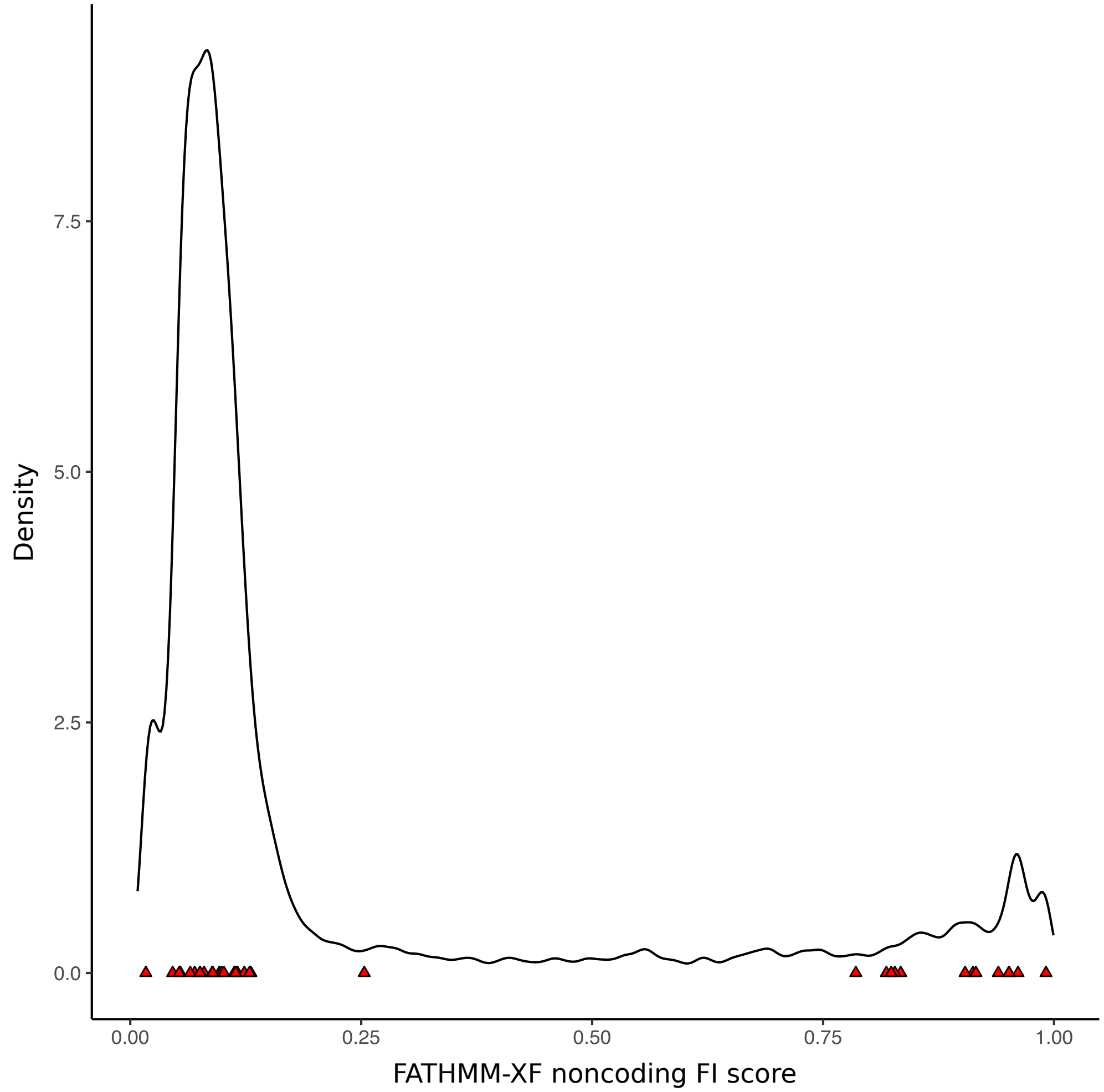

Fig AM: Distribution of functional impact scores in the ABC-derived BRCA1 CRRs. Density plot showing the distribution of possible functional impact scores within the ABC-derived BRCA1 CRRs. Red triangles show the scores of the observed SNPs in the CRRs of BRCA1.

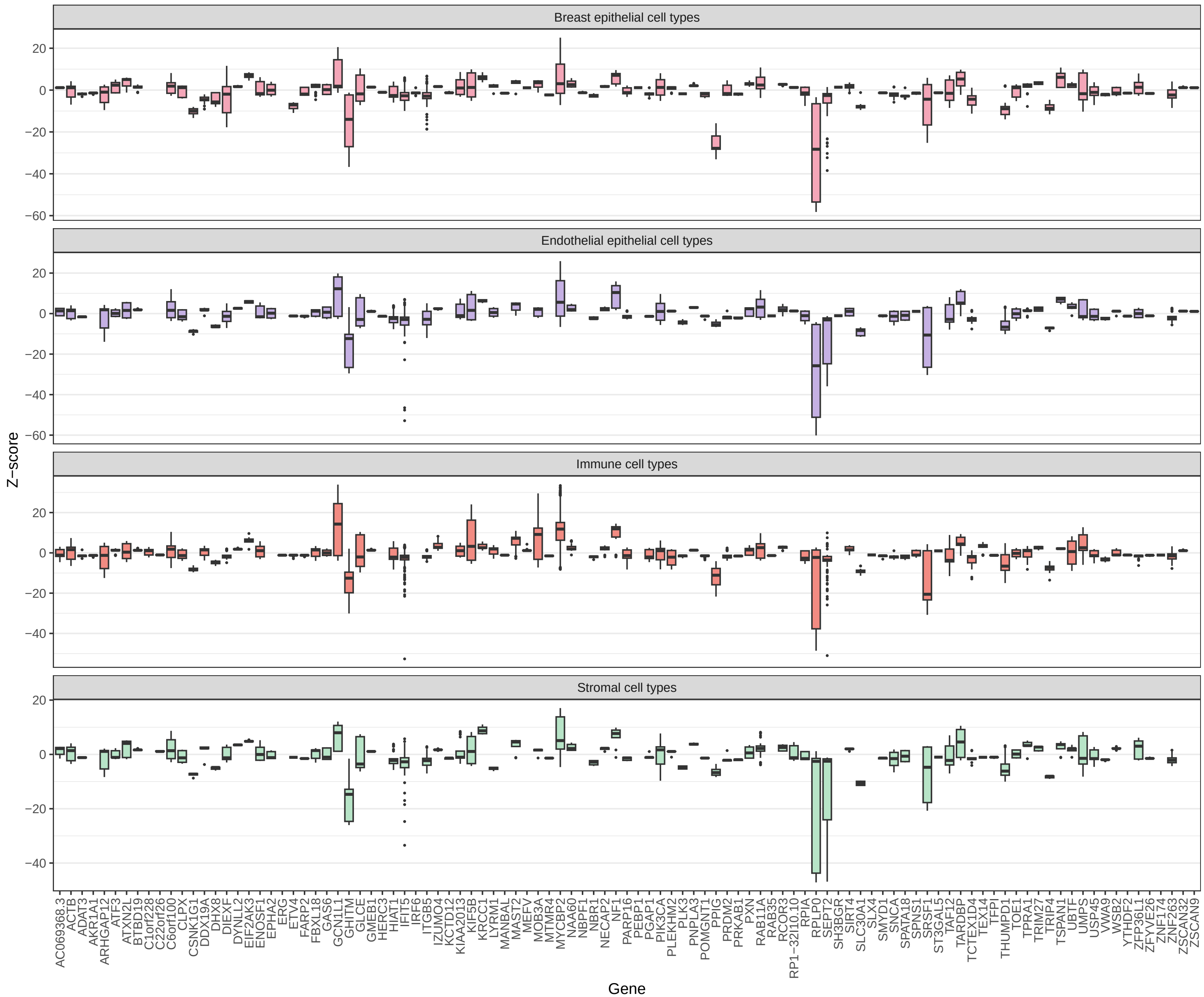

Fig AN: Discribution of ExPectoSC predicted effect of gene expression deregulation across SNPs per gene. ExPectoSC was used to predict the effect of SNPs associated with each gene predicted by HiFIseek (columns) on gene expression in cell types relevant to breast cancer (rows). For each gene and cell type combination, a box plot represents the distribution of ExPectoSC z-scores over the SNPs associated with the gene considered.

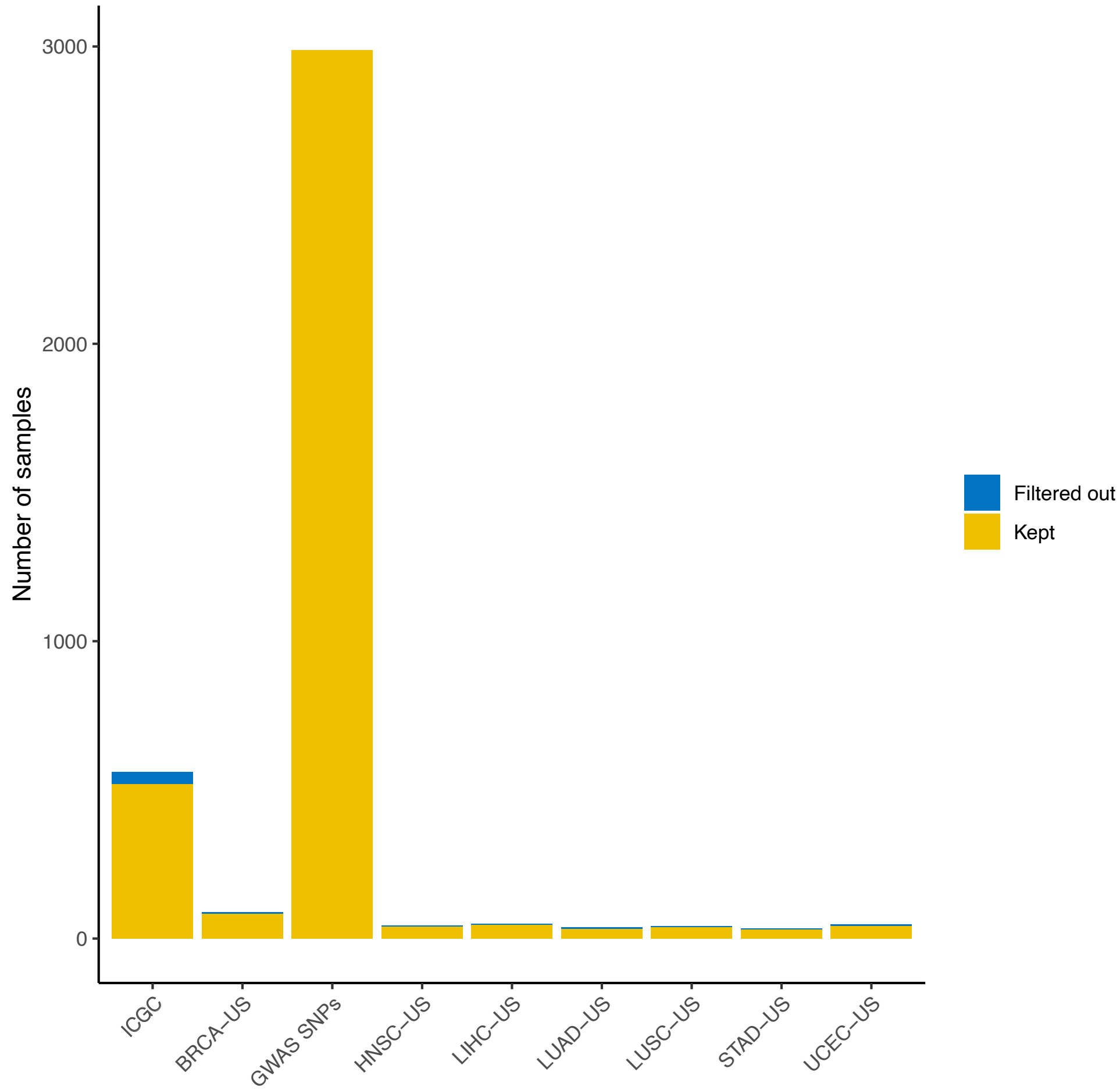

Fig AO: Numbers of samples per cohort before and after filtering. Number of samples (y-axis) by cohort (x-axis). Samples that passed the filtering step are colored in yellow, while filtered out samples are colored in blue.

Table A: Comparison of HiFIseek with existing tools for non-coding cis-regulatory variant (CRV) driver detection. CRR: cis-regulatory region; FI: functional impact.

**Tool (reference)**	**Regions analysed**	**CRR→gene linkage**	**FI scoring**	**Method**	**Multi CRR×FI?**	**Availability**
**HiFIseek** (this work)	User-provided	Multiple user-provided to run in parallel	Multiple user-provided to run in parallel	OncodriveFML + OncodriveFML-analytic (closed-form sum-of-scores, trinucleotide-aware null)	Yes, automated parallel assessments	Snakemake pipeline
**OncodriveFML** (Mularoni et al. 2016)	User-provided	A single user-provided set of regions	A single user-provided	Permutation: mean FI of observed vs. trinucleotide-matched random mutations	Not automated	CLI
**FunSeq2** (Fu et al. 2014; retired)	Variant-level analysis	Built-in enhancer-gene links; no gene-centered burden test	Internal weighted composite score (conservation, PWM-based analysis, functional annotation, network analysis, element reccurence)	Weighted score + reccurrence information across cancers / datasets; no specific background model	No	CLI (retired)
**DriverPower** (Shuai et al. 2020)	User-provided	A single user-provided set of regions	A single user-provided (mean value of CADD, DANN, EIGEN, LINSIGHT by default)	Two-stage: burden (randomized lasso + generalized linear model or gradient boosting machine; with 1373 covariate features) + posterior FI adjustment	No	CLI
**ActiveDriverWGS** (Zhu et al. 2020)	User-provided	A single user-provided set of regions	None (mutation burden is considered)	Poisson generalized linear regression with trinucleotide covariates and local background	No	R package
**DIGDriver** (Sherman et al. 2022)	User-provided	A single user-provided set of regions	None (mutation burden is considered)	Deep neural network learning mutation rates; gamma-Poisson test	No	CLI
