# Supplementary material for: HiFIseek: Gene-specific enrichment of high-impact mutations in associated genomic regions
Source: PLoS Comput Biol. 2026 Oct 6;22(10):e1014842. doi: 10.1371/journal.pcbi.1014842 (PMC13657309; doi:10.1371/journal.pcbi.1014842)
Supplement: Supplementary file 2 [file pcbi.1014842.s003.pdf]

We are thankful for the critical reading of our study and for the insightful comments, which have helped significantly improve the manuscript. We have addressed the suggested revisions and concerns in a revised manuscript with tracked edits and we provide a clean version as well. Overall, we have:

- Rewritten the Abstract and Introduction to better frame the lack of methodological consensus and HiFIseek's combinatorial approach
- Added a comparative table (Supplementary Table 1) positioning HiFIseek against existing driver-detection frameworks
- Clarified the rationale for OncodriveFML-analytic and corrected the earlier outlier claim
- Reported GO enrichment of biological processes when considering all predicted genes across CRR-gene association and FI scores combinations, which highlight cancer-related pathways
- Added multi-layered regulatory overview of the breast cancer risk variants (GTEx and TCGA eQTL analysis, ExPectoSC cell-type-specific transcriptional predictions, VEP regulatory annotation, and splicing predictions)
- Expanded the Discussion on the limitations of current CRR and FI annotations (cell-type specificity, incomplete coverage)
- Improved clarity and grammar
- Corrected the tool-name typo throughout and addressed all journal formatting requirements (Author Summary, Abstract heading, CRediT contributions, .tif figures, SI legends, Financial Disclosure)

Comments by the Editor:

Puig et al manuscript presents HiFIseek, a Snakemake-based computational pipeline developed to systematically detect gene-specific enrichment of high-impact cis-regulatory variants (CRVs) across associated genomic regions in cancer and disease datasets. The study addresses the challenge of identifying non-coding driver mutations that disrupt transcriptional regulation, leveraging multiple gene–cis-regulatory region (CRR) association methods and variant functional impact (FI) scoring systems. By comparing combinations of CRR–FI scoring approaches across eight cancer cohorts from TCGA/ICGC and breast cancer risk SNP datasets, the authors demonstrate that existing CRR and FI scoring methods show little consensus and that cancer genes tend to harbor more high-impact regulatory variants than non-cancer genes. HiFIseek introduces OncodriveFML-analytic, an analytical reformulation of the permutation-based OncodriveFML framework, offering improved computational efficiency while maintaining similar accuracy in enrichment detection. Application of the pipeline identified both known and potentially novel cancer-related genes, including BRCA1, that exhibit enrichment of high-impact non-coding variants
HiFIseek provides a comprehensive and modular framework for evaluating the enrichment of high-impact cis-regulatory mutations across diverse combinations of gene-CRR association and functional impact scoring methods, an area where methodological consensus has been lacking. This exhaustive combination testing offers a new way to assess the robustness of detected non-coding drivers and prioritize candidates supported by multiple independent methods. In addition, the introduction of OncodriveFML-analytic represents a significant technical

advancement: it analytically approximates the permutation-based OncodriveFML model, reducing computational time and resource use without compromising sensitivity.

Suggested revisions

1- The study reports that many enriched genes did not show differential expression. The authors should discuss potential biological explanations (e.g., post-transcriptional regulation, chromatin effects) and consider integrating additional regulatory evidence (e.g., eQTL or chromatin accessibility data) to validate functional relevance.

In the revised manuscript, we performed complementary analyses focusing on risk variants identified by HiFIseek enriched for high-impact non-protein-coding variants associated with known breast cancer-related genes. We specifically examined alternative mechanisms of action, with a specific focus on transcriptional effects. Several complementary datasets and *in silico* tools were used to assess such effects. **(i)** First, variant-eQTL overlap was assessed using GTEx breast tissue data, where 4 variants matched known eQTLs. **(ii)** For broader validation, the TCGA dataset (n=653 individuals of White and non-Hispanic Latino ancestry; excluding metastatic and solid tissue normal samples) was used. At the cis-level, 10 variants acted as eQTLs, while 422 hits were identified at the trans-eQTL level, both after Benjamini-Hochberg adjustment. **(iii)** To capture transcriptional effects beyond conventional eQTL mapping, we applied ExPectoSC, a machine-learning pipeline trained on single-cell RNA-seq data from a breast model (Kumar et al. 2023). ExpectoSC predicted transcriptional effects for 347 of the 1,026 variants (|z-score| >1), affecting the expression of 103 genes across four breast-relevant cell types: epithelial, immune, endothelial, and stromal. Of these, 205 variants had predicted effects across all four cell types; the remainder showed cell-type-specificity (n=27 immune-specific, n=26 breast epithelial-specific, n=10 stromal and n=2 endothelial) or partially shared patterns (n=26 shared between endothelial and breast epithelial, n=40 shared between immune and breast epithelial, n=9 shared between immune and stromal, etc.). **(iv)** Regulatory region annotation was further explored using the Ensembl Variant Effect Predictor (VEP), which implicated 717 variants (69.8%) in at least one regulatory region. Because variants could be assigned to multiple regulatory categories, the counts are not mutually exclusive. Regulatory annotations included enhancers (n=521), promoters (n=193), CTCF binding sites (n=9), and open chromatin regions (n=2). These predictions indicate that these variants may modulate chromatin architecture rather than directly affect transcript levels. **(v)** Finally, the *in silico* splicing tools SpliceAI, MaxEntScan, and dbscSV identified 9 variants with predicted functional impact. Together, the identified prediction results provide multi-layered regulatory evidence supporting the functional relevance of identified variants, even in the absence of direct overlaps between layers.

2- Including a brief comparative table contrasting HiFIseek's capabilities with existing frameworks such as OncodriveFML, FunSeq2, ActiveDriver or ExPecto would highlight its unique advantages and contextualize its contribution to the field.

Thank you for the suggestion to contextualise HiFIseek against existing frameworks. We have surveyed the landscape of non-coding / cis-regulatory variant driver-detection methods

(oncodriveFML, FunSeq2, DriverPower, ActiveDriveWGS, and DIGDriver), which is now summarised in Supplementary Table 1 (referenced in the Results section introducing HiFIseek). No existing tool systematically reports gene-level enrichment results across combinations of CRR-gene and FI methods in an automated way, which is one of HiFIseek's contributions. We did not include ExPecto and ExPectoSC, which you specifically mentioned, as they are sequence-based variant-effect predictors using sequence-to-function modelling rather than cohort-level driver-detection methods; they could serve as FI scoring methods to be plugged into HiFIseek.

3- The discussion should explicitly address the limitations of using existing CRR and FI annotations (e.g., cell-type specificity, incomplete coverage) and how they might influence results or reproducibility across datasets.

As suggested, we have added some information about this aspect to the Discussion, acknowledging that the lack of observed expression changes could be explained by limitations of bulk RNA-seq and the cell-type specificity of CRR annotations. We note that the variants could act through mechanisms not captured by overall expression levels, such as splicing, isoform usage, or chromatin state changes, or may only be functional in cell subpopulations diluted in bulk data. Full integration of ATAC-seq, eQTL, or isoform-level data is beyond the scope of this work but is noted as a priority for follow-up.

4- Some figures (e.g., Figure 2) are dense and could benefit from clearer legends or subpanels.

Thanks for the comment. While we agree that figures like Figure 2 are dense, we believe it is necessary to provide a complete overview of the comparative results. We have revised the legends to better describe the figures.

Journal Requirements:

1) Please ensure that the CRediT author contributions listed for every co-author are completed accurately and in full.
At this stage, the following Authors/Authors require contributions: Rafael Riudavets Puig, Ina Skaara Brorson, Denise G. O'Mahony, Vessela N. Kristensen, and Anthony Mathelier. Please ensure that the full contributions of each author are acknowledged in the "Add/Edit/Remove Authors" section of our submission form.
The list of CRediT author contributions may be found here:
https://journals.plos.org/ploscompbiol/s/authorship#loc-author-contributions

We have added an Author Contributions section following the CRediT taxonomy.

2) We ask that a manuscript source file is provided at Revision. Please upload your manuscript file as a .doc, .docx, .rtf or .tex. If you are providing a .tex file, please upload it under the item type u2018LaTeX Source Fileu2019 and leave your .pdf version as the item type u2018Manuscriptu2019.

A .docx file has been submitted.

3) Please provide an Author Summary. This should appear in your manuscript between the Abstract (if applicable) and the Introduction, and should be 150-200 words long. The aim should be to make your findings accessible to a wide audience that includes both scientists and non-scientists. Sample summaries can be found on our website under Submission Guidelines: https://journals.plos.org/ploscompbiol/s/submission-guidelines#loc-parts-of-a-submission

We have added an Author Summary section.

4) Your manuscript is missing the following section heading: Abstract. Please ensure all required sections are present and in the correct order. Make sure section heading levels are clearly indicated in the manuscript text, and limit sub-sections to 3 heading levels. An outline of the required sections can be consulted in our submission guidelines here: https://journals.plos.org/ploscompbiol/s/submission-guidelines#loc-parts-of-a-submission

We have explicitly included a section heading for the Abstract.

5) Please upload all main figures as separate Figure files in .tif or .eps format. For more information about how to convert and format your figure files please see our guidelines: https://journals.plos.org/ploscompbiol/s/figures

We have now uploaded all figures as .tif files.

6) We have noticed that you have uploaded Supporting Information files, but you have not included a list of legends. Please add a full list of legends for your Supporting Information files after the references list.

We have incorporated this section.

7) Please amend your detailed Financial Disclosure statement. This is published with the article. It must therefore be completed in full sentences and contain the exact wording you wish to be published.
1) State what role the funders took in the study. If the funders had no role in your study, please state: "The funders had no role in study design, data collection and analysis, decision to publish, or preparation of the manuscript."

We have updated this section accordingly.

8) Please ensure that the funders and grant numbers match between the Financial Disclosure field and the Funding Information tab in your submission form. Note that the funders must be provided in the same order in both places as well.

Note: If the reviewer comments include a recommendation to cite specific previously published works, please review and evaluate these publications to determine whether they are relevant and should be cited. There is no requirement to cite these works unless the editor has indicated otherwise.

Reviewers' comments:

Reviewer's Responses to Questions

Reviewer #1: Overall Summary:
The manuscript by Riudavets Puig et al. presents HiFIseek, a computational pipeline designed to systematically identify genes enriched for high functional impact (FI) cis-regulatory variants (CRVs) by exploring multiple combinations of gene-region association and FI scoring methods. The authors first convincingly demonstrate a critical, yet underappreciated, challenge in the field: the profound lack of consensus across different gene-CRR association and FI scoring methods. The development of HiFIseek and the complementary analytical method, OncodriveFML-analytic, represents a valuable and pragmatic approach to this problem. The application to both cancer cohorts and breast cancer risk SNPs yields interesting, if complex, results. While the tool is well-motivated and the analysis is comprehensive, several major points regarding the biological interpretation and validation of the findings need to be addressed to strengthen the manuscript's impact.

1- The abstract should be revised to immediately frame the study around this critical problem: the high variability in results depending on the choice of gene-CRR and FI scoring method, and the consequent need for a systematic, combinatorial approach. The introduction should more forcefully present HiFIseek as a direct solution to this problem of methodological consensus, rather than just another enrichment tool. Explicitly state that the key novelty is not just the pipeline itself, but the philosophy of agnostically combining all available methods to find robust signals.

We thank the Reviewer for this framing suggestion and agree that the original Abstract and Introduction underemphasised the methodological problem motivating HiFIseek. We have rewritten both sections accordingly.

2- There is a consistent typo in the name of the tool. It is introduced as "HiFIseek" in the abstract and title but is often written as "HiFiseek" or "HiFJseek" in the body of the manuscript (e.g., pages 6, 7, 8, 9). This must be corrected to "HiFIseek" throughout for professionalism and clarity.

Thanks for pointing that out. We have made sure that the revision only contains the following spelling HiFIseek.

3- The Results section (page 11) should include a more intuitive explanation of the key difference—using the sum of scores versus the mean—and why this makes the method less sensitive to outliers and computationally more efficient. A sentence or two summarizing the mathematical rationale would be helpful for a broad computational biology audience.

Thank you for raising this as it allows us to correct a mistake in the original submission. Indeed, the sum versus the mean does not make the method less sensitive to outliers but reflects the potential accumulation of high-impact variants in CRRs. Moreover, the distribution of a sum of independent scores is well approximated by a normal distribution, which allows the p-values to be computed in closed form from the per-variant score distribution with the cohort's mutational signature, removing the need for permutations. We have updated the text in the Results section to provide this information.

4- The description of the new OncodriveFML-analytic method, while detailed in the methods, could be more accessible in the main text.

Following the previous comment, we have updated the text describing the OncodriveFML-analytic method. We hope this high-level description provides a more accessible information in the Results section with explicit details in the Methods section.

5- The gene set enrichment analysis on genes identified in cancer cohorts did not yield cancer-related terms. This is a surprising result that somewhat undermines the premise that these enriched CRVs are driving cancer. The authors should investigate this further. Are the 210 genes from the cancer cohorts enriched for any other biological processes? If they are not enriched for cancer pathways, what biological processes are they involved in? This analysis is crucial for interpreting the biological relevance of the genes prioritized by HiFIseek in the somatic mutation context.

Thanks for the comment. We further investigated this and found that the lack of enrichment was linked to the arbitrary stringent criterion of considering only genes found in at least five combinations of score, region, and/or enrichment method. When considering all predicted genes across the different combinations, we found GO biological processes recurrently predicted to be enriched across the cohorts and that are known to be associated with cancer initiation or progression, such as signaling, apoptotic process, cell cycle and cell proliferation-related terms. We have now incorporated these results into the Results section.

6- The authors should provide a deeper discussion of potential reasons for this discrepancy. Are the variants acting in a cell-type or condition-specific manner not captured by the bulk RNA-seq? Could they be affecting other aspects of gene regulation (e.g., splicing, isoform usage, chromatin state) rather than overall expression levels? Including additional analyses, such as looking at isoform-level expression or correlating variant presence with chromatin accessibility data (e.g., ATAC-seq) where available, would significantly strengthen the biological claims.

As outlined in our response to the first comment of the editor, the results of the risk SNPs exploration have highlighted variant transcriptional effects at the bulk and cell-type-specific level. Specifically, using the predictions from ExPectoSC, we show that although 205 variants had transcriptional effects across all four cell type analyses (breast epithelial, immune, endothelial and stromal), the remainder showed cell-type-specific or partially shared patterns (e.g., n=27 immune-specific, n=26 breast epithelial-specific, n=26 shared between endothelial and breast epithelial, n=40 shared between immune and breast epithelial). Additionally, SpliceAI identified n=9 variants with predicted splicing effects, suggesting that a subset may act through isoform usage rather than overall expression levels. Chromatin annotation via VEP further implicated most variants in regulatory elements, including enhancers and promoters, consistent with chromatin-mediated mechanisms. Isoform-level expression analysis was not performed in the current study and is an avenue for future investigation.
