# Supplementary material for: HiFIseek: Gene-specific enrichment of high-impact mutations in associated genomic regions
Source: PLoS Comput Biol. 2026 Oct 6;22(10):e1014842. doi: 10.1371/journal.pcbi.1014842 (PMC13657309; doi:10.1371/journal.pcbi.1014842)
Supplement: Peer review history

# Peer review: Decision Letter 0

**DOI:** 10.1371/journal.pcbi.1014842.r001

**Authors:** Ilya Ioshikhes, Mohamed Helmy

24 Nov 2025

PCOMPBIOL-D-25-01228

HiFIseek: gene-specific enrichment of high-impact mutations in associated genomic regions

PLOS Computational Biology

Dear Dr. Mathelier,

Thank you for submitting your manuscript to PLOS Computational Biology. After careful consideration, we feel that it has merit but does not fully meet PLOS Computational Biology's publication criteria as it currently stands. Therefore, we invite you to submit a revised version of the manuscript that addresses the points raised during the review process.

Please submit your revised manuscript by Jan 24 2026 11:59PM. If you will need more time than this to complete your revisions, please reply to this message or contact the journal office at ploscompbiol@plos.org. When you're ready to submit your revision, log on to https://www.editorialmanager.com/pcompbiol/ and select the 'Submissions Needing Revision' folder to locate your manuscript file.

Please include the following items when submitting your revised manuscript:

* A rebuttal letter that responds to each point raised by the editor and reviewer(s). You should upload this letter as a separate file labeled 'Response to Reviewers'. This file does not need to include responses to formatting updates and technical items listed in the 'Journal Requirements' section below.

* A marked-up copy of your manuscript that highlights changes made to the original version. You should upload this as a separate file labeled 'Revised Manuscript with Track Changes'.

* An unmarked version of your revised paper without tracked changes. You should upload this as a separate file labeled 'Manuscript'.

If you would like to make changes to your financial disclosure, competing interests statement, or data availability statement, please make these updates within the submission form at the time of resubmission. Guidelines for resubmitting your figure files are available below the reviewer comments at the end of this letter

We look forward to receiving your revised manuscript.

Kind regards,

Mohamed Helmy, PhD

Academic Editor

PLOS Computational Biology

Ilya Ioshikhes

Section Editor

PLOS Computational Biology

**Additional Editor Comments :**

Comments by the Editor:

Puig et al manuscript presents HiFIseek, a Snakemake-based computational pipeline developed to systematically detect gene-specific enrichment of high-impact cis-regulatory variants (CRVs) across associated genomic regions in cancer and disease datasets. The study addresses the challenge of identifying non-coding driver mutations that disrupt transcriptional regulation, leveraging multiple gene–cis-regulatory region (CRR) association methods and variant functional impact (FI) scoring systems. By comparing combinations of CRR–FI scoring approaches across eight cancer cohorts from TCGA/ICGC and breast cancer risk SNP datasets, the authors demonstrate that existing CRR and FI scoring methods show little consensus and that cancer genes tend to harbor more high-impact regulatory variants than non-cancer genes. HiFIseek introduces OncodriveFML-analytic, an analytical reformulation of the permutation-based OncodriveFML framework, offering improved computational efficiency while maintaining similar accuracy in enrichment detection. Application of the pipeline identified both known and potentially novel cancer-related genes, including BRCA1, that exhibit enrichment of high-impact non-coding variants

HiFIseek provides a comprehensive and modular framework for evaluating the enrichment of high-impact cis-regulatory mutations across diverse combinations of gene-CRR association and functional impact scoring methods, an area where methodological consensus has been lacking. This exhaustive combination testing offers a new way to assess the robustness of detected non-coding drivers and prioritize candidates supported by multiple independent methods. In addition, the introduction of OncodriveFML-analytic represents a significant technical advancement: it analytically approximates the permutation-based OncodriveFML model, reducing computational time and resource use without compromising sensitivity.

Suggested revisions

1- The study reports that many enriched genes did not show differential expression. The authors should discuss potential biological explanations (e.g., post-transcriptional regulation, chromatin effects) and consider integrating additional regulatory evidence (e.g., eQTL or chromatin accessibility data) to validate functional relevance.

2- Including a brief comparative table contrasting HiFIseek’s capabilities with existing frameworks such as OncodriveFML, FunSeq2, ActiveDriver or ExPecto would highlight its unique advantages and contextualize its contribution to the field

3- The discussion should explicitly address the limitations of using existing CRR and FI annotations (e.g., cell-type specificity, incomplete coverage) and how they might influence results or reproducibility across datasets.

4- Some figures (e.g., Figure 2) are dense and could benefit from clearer legends or subpanels.

**Journal Requirements:**

1) Please ensure that the CRediT author contributions listed for every co-author are completed accurately and in full.

At this stage, the following Authors/Authors require contributions: Rafael Riudavets Puig, Ina Skaara Brorson, Denise G. O'Mahony, Vessela N. Kristensen, and Anthony Mathelier. Please ensure that the full contributions of each author are acknowledged in the "Add/Edit/Remove Authors" section of our submission form.

The list of CRediT author contributions may be found here: https://journals.plos.org/ploscompbiol/s/authorship#loc-author-contributions

2) We ask that a manuscript source file is provided at Revision. Please upload your manuscript file as a .doc, .docx, .rtf or .tex. If you are providing a .tex file, please upload it under the item type u2018LaTeX Source Fileu2019 and leave your .pdf version as the item type u2018Manuscriptu2019.

3) Please provide an Author Summary. This should appear in your manuscript between the Abstract (if applicable) and the Introduction, and should be 150-200 words long. The aim should be to make your findings accessible to a wide audience that includes both scientists and non-scientists. Sample summaries can be found on our website under Submission Guidelines:

https://journals.plos.org/ploscompbiol/s/submission-guidelines#loc-parts-of-a-submission

4) Your manuscript is missing the following section heading: Abstract.  Please ensure all required sections are present and in the correct order. Make sure section heading levels are clearly indicated in the manuscript text, and limit sub-sections to 3 heading levels. An outline of the required sections can be consulted in our submission guidelines here:

https://journals.plos.org/ploscompbiol/s/submission-guidelines#loc-parts-of-a-submission

5) Please upload all main figures as separate Figure files in .tif or .eps format. For more information about how to convert and format your figure files please see our guidelines:

https://journals.plos.org/ploscompbiol/s/figures

6) We have noticed that you have uploaded Supporting Information files, but you have not included a list of legends. Please add a full list of legends for your Supporting Information files after the references list.

7) Please amend your detailed Financial Disclosure statement. This is published with the article. It must therefore be completed in full sentences and contain the exact wording you wish to be published.

1) State what role the funders took in the study. If the funders had no role in your study, please state: "The funders had no role in study design, data collection and analysis, decision to publish, or preparation of the manuscript."

8) Please ensure that the funders and grant numbers match between the Financial Disclosure field and the Funding Information tab in your submission form. Note that the funders must be provided in the same order in both places as well.

Note: If the reviewer comments include a recommendation to cite specific previously published works, please review and evaluate these publications to determine whether they are relevant and should be cited. There is no requirement to cite these works unless the editor has indicated otherwise.

**Reviewers' comments:**

Reviewer's Responses to Questions

Reviewer #1: Overall Summary:

The manuscript by Riudavets Puig et al. presents HiFIseek, a computational pipeline designed to systematically identify genes enriched for high functional impact (FI) cis-regulatory variants (CRVs) by exploring multiple combinations of gene-region association and FI scoring methods. The authors first convincingly demonstrate a critical, yet underappreciated, challenge in the field: the profound lack of consensus across different gene-CRR association and FI scoring methods. The development of HiFIseek and the complementary analytical method, OncodriveFML-analytic, represents a valuable and pragmatic approach to this problem. The application to both cancer cohorts and breast cancer risk SNPs yields interesting, if complex, results. While the tool is well-motivated and the analysis is comprehensive, several major points regarding the biological interpretation and validation of the findings need to be addressed to strengthen the manuscript's impact.

1- The abstract should be revised to immediately frame the study around this critical problem: the high variability in results depending on the choice of gene-CRR and FI scoring method, and the consequent need for a systematic, combinatorial approach. The introduction should more forcefully present HiFIseek as a direct solution to this problem of methodological consensus, rather than just another enrichment tool. Explicitly state that the key novelty is not just the pipeline itself, but the philosophy of agnostically combining all available methods to find robust signals.

2- There is a consistent typo in the name of the tool. It is introduced as "HiFIseek" in the abstract and title but is often written as "HiFiseek" or "HiFJseek" in the body of the manuscript (e.g., pages 6, 7, 8, 9). This must be corrected to "HiFIseek" throughout for professionalism and clarity.

3- The Results section (page 11) should include a more intuitive explanation of the key difference—using the sum of scores versus the mean—and why this makes the method less sensitive to outliers and computationally more efficient. A sentence or two summarizing the mathematical rationale would be helpful for a broad computational biology audience.

4- The description of the new OncodriveFML-analytic method, while detailed in the methods, could be more accessible in the main text.

5- The gene set enrichment analysis on genes identified in cancer cohorts did not yield cancer-related terms. This is a surprising result that somewhat undermines the premise that these enriched CRVs are driving cancer. The authors should investigate this further. Are the 210 genes from the cancer cohorts enriched for any other biological processes? If they are not enriched for cancer pathways, what biological processes are they involved in? This analysis is crucial for interpreting the biological relevance of the genes prioritized by HiFIseek in the somatic mutation context.

6- The authors should provide a deeper discussion of potential reasons for this discrepancy. Are the variants acting in a cell-type or condition-specific manner not captured by the bulk RNA-seq? Could they be affecting other aspects of gene regulation (e.g., splicing, isoform usage, chromatin state) rather than overall expression levels? Including additional analyses, such as looking at isoform-level expression or correlating variant presence with chromatin accessibility data (e.g., ATAC-seq) where available, would significantly strengthen the biological claims.

**********

**Have the authors made all data and (if applicable) computational code underlying the findings in their manuscript fully available?**

The PLOS Data policy requires authors to make all data and code underlying the findings described in their manuscript fully available without restriction, with rare exception (please refer to the Data Availability Statement in the manuscript PDF file). The data and code should be provided as part of the manuscript or its supporting information, or deposited to a public repository. For example, in addition to summary statistics, the data points behind means, medians and variance measures should be available. If there are restrictions on publicly sharing data or code —e.g. participant privacy or use of data from a third party—those must be specified.

Reviewer #1: Yes

**********

PLOS authors have the option to publish the peer review history of their article (what does this mean?). If published, this will include your full peer review and any attached files.

If you choose “no”, your identity will remain anonymous but your review may still be made public.

**Do you want your identity to be public for this peer review?**  For information about this choice, including consent withdrawal, please see our Privacy Policy.

Reviewer #1: No

[NOTE: If reviewer comments were submitted as an attachment file, they will be attached to this email and accessible via the submission site. Please log into your account, locate the manuscript record, and check for the action link "View Attachments". If this link does not appear, there are no attachment files.]

**Figure resubmission:**

While revising your submission, we strongly recommend that you use PLOS’s NAAS tool (https://ngplosjournals.pagemajik.ai/artanalysis) to test your figure files. NAAS can convert your figure files to the TIFF file type and meet basic requirements (such as print size, resolution), or provide you with a report on issues that do not meet our requirements and that NAAS cannot fix.

After uploading your figures to PLOS’s NAAS tool - https://ngplosjournals.pagemajik.ai/artanalysis, NAAS will process the files provided and display the results in the "Uploaded Files" section of the page as the processing is complete. If the uploaded figures meet our requirements (or NAAS is able to fix the files to meet our requirements), the figure will be marked as "fixed" above. If NAAS is unable to fix the files, a red "failed" label will appear above. When NAAS has confirmed that the figure files meet our requirements, please download the file via the download option, and include these NAAS processed figure files when submitting your revised manuscript.

**Reproducibility:**

To enhance the reproducibility of your results, we recommend that authors of applicable studies deposit laboratory protocols in protocols.io, where a protocol can be assigned its own identifier (DOI) such that it can be cited independently in the future. Additionally, PLOS ONE offers an option to publish peer-reviewed clinical study protocols. Read more information on sharing protocols at https://plos.org/protocols?utm_medium=editorial-email&utm_source=authorletters&utm_campaign=protocols

# Author comment: Author response to Decision Letter 1

**DOI:** 10.1371/journal.pcbi.1014842.r002

6 Jul 2026

Attachment Submitted filename: HiFIseek_rebuttal_PLOS_CB.pdf

# Peer review: Decision Letter 1

**DOI:** 10.1371/journal.pcbi.1014842.r003

**Authors:** Ilya Ioshikhes

22 Sep 2026

Dear Dr. Mathelier,

We are pleased to inform you that your manuscript 'HiFIseek: gene-specific enrichment of high-impact mutations in associated genomic regions' has been provisionally accepted for publication in PLOS Computational Biology.

Before your manuscript can be formally accepted you will need to complete some formatting changes, which you will receive in a follow up email. A member of our team will be in touch with a set of requests.

Please note that your manuscript will not be scheduled for publication until you have made the required changes, so a swift response is appreciated.

IMPORTANT: The editorial review process is now complete. PLOS will only permit corrections to spelling, formatting or significant scientific errors from this point onwards. Requests for major changes, or any which affect the scientific understanding of your work, will cause delays to the publication date of your manuscript.

Should you, your institution's press office or the journal office choose to press release your paper, you will automatically be opted out of early publication. We ask that you notify us now if you or your institution is planning to press release the article. All press must be co-ordinated with PLOS.

Thank you again for supporting Open Access publishing; we are looking forward to publishing your work in PLOS Computational Biology.

Best regards,

Ilya Ioshikhes

Section Editor

PLOS Computational Biology

Ilya Ioshikhes

Section Editor

PLOS Computational Biology

***********************************************************

Reviewer's Responses to Questions

**Comments to the Authors:**

**Please note here if the review is uploaded as an attachment.**

Reviewer #1: Accept

**********

**Have the authors made all data and (if applicable) computational code underlying the findings in their manuscript fully available?**

The PLOS Data policy requires authors to make all data and code underlying the findings described in their manuscript fully available without restriction, with rare exception (please refer to the Data Availability Statement in the manuscript PDF file). The data and code should be provided as part of the manuscript or its supporting information, or deposited to a public repository. For example, in addition to summary statistics, the data points behind means, medians and variance measures should be available. If there are restrictions on publicly sharing data or code —e.g. participant privacy or use of data from a third party—those must be specified.

Reviewer #1: None

**********

PLOS authors have the option to publish the peer review history of their article (what does this mean?). If published, this will include your full peer review and any attached files.

If you choose “no”, your identity will remain anonymous but your review may still be made public.

**Do you want your identity to be public for this peer review?**  For information about this choice, including consent withdrawal, please see our Privacy Policy.

Reviewer #1: No

# Editor report: Acceptance letter

**DOI:** 10.1371/journal.pcbi.1014842.r004

**Authors:** Ilya Ioshikhes

PCOMPBIOL-D-25-01228R1

HiFIseek: gene-specific enrichment of high-impact mutations in associated genomic regions

Dear Dr Mathelier,

I am pleased to inform you that your manuscript has been formally accepted for publication in PLOS Computational Biology. Your manuscript is now with our production department and you will be notified of the publication date in due course.

The corresponding author will soon be receiving a typeset proof for review, to ensure errors have not been introduced during production. Please review the PDF proof of your manuscript carefully, as this is the last chance to correct any errors. Please note that major changes, or those which affect the scientific understanding of the work, will likely cause delays to the publication date of your manuscript.

Soon after your final files are uploaded, unless you have opted out, the early version of your manuscript will be published online. The date of the early version will be your article's publication date. The final article will be published to the same URL, and all versions of the paper will be accessible to readers.

For Research, Software, and Methods articles, you will receive an invoice from PLOS for your publication fee after your manuscript has reached the completed accept phase. If you receive an email requesting payment before acceptance or for any other service, this may be a phishing scheme. Learn how to identify phishing emails and protect your accounts at https://explore.plos.org/phishing.

Thank you again for supporting PLOS Computational Biology and open-access publishing. We are looking forward to publishing your work!

With kind regards,

Sharmila Kamatchi

PLOS Computational Biology | Carlyle House, Carlyle Road, Cambridge CB4 3DN | United Kingdom ploscompbiol@plos.org | Phone +44 (0) 1223-442824 | ploscompbiol.org | @PLOSCompBiol
